# Global, regional, and national burden of early-onset and late-onset colorectal cancer attributable to high body-mass index from 1990 to 2021: a trend analysis and forecasts up to 2040 based on the global burden of disease study 2021

**DOI:** 10.1186/s12876-025-04432-7

**Published:** 2025-11-19

**Authors:** Bin Yue, Zhongqiao Lu, Deshan Zong, Yingxia Hu, Zhongde Yang

**Affiliations:** 1 Department of Gastroenterology, The People’s Hospital of Wenshan Prefecture; Affiliated Wenshan Hospital, KunMing University of Science and Technology, No. 31, Tenglong North Road, Wenshan, Yunnan Province 663000 China; 2 Department of Cardiovascular Diseases, The People’s Hospital of Wenshan Prefecture, Affiliated Wenshan Hospital, KunMing University of Science and Technology, Wenshan, Yunnan Province 663000 China

**Keywords:** Early-onset colorectal cancer, Late-onset colorectal cancer, Disability-adjusted life years, High body-mass index, Age-standardized rate, Disease burden

## Abstract

**Background:**

The global burden of colorectal cancer (CRC) has been steadily rising. However, a key knowledge gap persists regarding the HBMI-attributable burden in early-onset (EOCRC) versus late-onset (LOCRC) cases. The temporal patterns, geographic heterogeneity, and comparative trends remain poorly characterized, as prior studies often lack stratification by age of onset and detailed analysis across socio-demographic strata.

**Objective:**

This investigation seeks to systematically quantify the burden of EOCRC and LOCRC attributable to HBMI at global, regional, and national levels from 1990 to 2021 and provides projections of the burden through 2040.

**Methods:**

Epidemiological data, including number and rate for EOCRC and LOCRC were obtained from the Global Burden of Disease Study (GBD) 2021. Direct standardization method was employed to standardize rates across age-specific groups. The estimated annual percentage change (EAPC) model was utilized to analyze temporal trends from 1990 to 2021 across various countries, genders, SDI and GBD regions. Additionally, Nordpred and Bayesian age-period-cohort (BAPC) models were employed to forecast the burden of EOCRC and LOCRC owing to HBMI through 2040, both in terms of numbers and age-standardized rate (ASR).

**Results:**

In 2021, HBMI was responsible for 359,538 disability-adjusted life years (DALYs) and 7,255 deaths in EOCRC, as well as 2,005,125 DALYs and 92,013 deaths in LOCRC. From 1990 to 2021, there was an increase of 130.97% in DALYs and 130.24% in deaths associated with EOCRC, while LOCRC saw rises of 133.32% and 139.71%, respectively. The global age-standardized DALYs rate (ASDR) and age-standardized mortality rate (ASMR) both exhibited an upward trend in EOCRC, regardless of gender. Conversely, in LOCRC, the global ASDR remained stable, while the ASMR exhibited a slight decline, a trend that was more evident in females. A declining trend was noted specifically among females in high-middle socio-demographic index (SDI) regions for EOCRC, and across both genders in high SDI regions combine females in high-middle SDI regions for LOCRC, the remaining SDI regions demonstrated varying degrees of upward trends in both ASDR and ASMR. Among GBD regions, High-income North America had the highest ASDR and ASMR for EOCRC, while Central Europe recorded the highest ASDR and ASMR for LOCRC. In contrast, East Asia reported the highest number of DALYs and deaths for both EOCRC and LOCRC. Furthermore, a nonlinear relationship was observed between the ASR and SDI in GBD regions for both EOCRC and LOCRC attributable to HBMI. Projections based on Nordpred and BAPC models revealed a consistent global increase in DALYs, deaths, ASDR, and ASMR for both EOCRC and LOCRC.

**Conclusion:**

From 1990 to 2021, the global burden of LOCRC attributable to HBMI remains significantly high and consistently exceeds that of EOCRC, but the rapid growth of EOCRC should be emphasized. The burden of CRC is closely associated with the SDI. While LOCRC has begun to decline in high-SDI regions, EOCRC continues to increase across all SDI regions. Over the next 20 years, the burden caused by EOCRC and LOCRC is expected to continue increasing.

**Supplementary Information:**

The online version contains supplementary material available at 10.1186/s12876-025-04432-7.

## Background

Cancer represents a significant public health and socioeconomic challenge in the current era, accounting for approximately one-quarter of all deaths due to non-communicable diseases globally. Colorectal carcinoma represents the most prevalent malignancy in the digestive system, both in terms of incidence and mortality [1]. According to statistics from 2022, colorectal carcinoma ranked as the third most prevalent malignancy globally, accounting for 1,926,118 incident cases, which represented 9.6% of total cancer diagnoses. In terms of mortality, this neoplasm constituted the second most frequent cause of cancer-associated fatalities, with 903,859 deaths corresponding to 9.3% of total cancer mortality [2]. CRC is typically categorized into EOCRC for individuals under 50 years of age and LOCRC for those 50 years or older. This classification reflects differences in tumor pathology, patient outcomes, and colorectal screening age recommendations across countries [3, 4]. While global CRC incidence has generally declined, the incidence of EOCRC is increasing rapidly. However, the distribution and trends of EOCRC across regions and countries, particularly in comparison to LOCRC, remain poorly understood [4, 5].

In recent years, the prevalence of HBMI has risen significantly worldwide. Since 1980, the global prevalence of overweight and obesity has nearly doubled, now affecting approximately 30% of the population. This increase is particularly pronounced in developed countries, while developing nations are also experiencing rapid growth in obesity rates [6]. HBMI is strongly linked to various non-neoplastic conditions, including metabolic disorders, cardiovascular and cerebrovascular pathologies and chronic kidney disease [7]. Additionally, it is widely recognized as a risk factor for multiple cancers, such as breast, endometrial, and colorectal cancer [8]. HBMI promotes cancer development through various mechanisms, encompassing chronic low-grade inflammation, insulin resistance accompanied by hyperinsulinemia, and pro-inflammatory factors secreted by adipose tissue [9, 10]. The effects of HBMI have been observed in both EOCRC and LOCRC. Evidence indicates that HBMI may represent a significant modifiable risk determinant for EOCRC, with its influence may occur through prolonged exposure and interactions with metabolic syndrome [11]. In comparison, the association between HBMI and LOCRC is more evident, with a linear correlation between increased BMI and CRC risk, especially in men and colon cancer patients [12]. These findings imply that HBMI may involve both common and distinct pathological mechanisms in EOCRC and LOCRC.

Prior research on the CRC burden attributable to HBMI and the unique epidemiology of EOCRC is limited in two key aspects. Much of the work does not stratify by age of onset, failing to capture the divergent trends and risk factor profiles distinguishing EOCRC from LOCRC [13]. Moreover, studies focusing on EOCRC frequently omit a granular analysis of how the burden attributable to specific risk factors like HBMI varies across socio-demographic groups [14]. Consequently, the development of precisely targeted, age-specific prevention and control strategies remains hindered.

The GBD 2021 included 371 diseases and injuries across 204 countries and territories, providing an opportunity to assess the incidence, prevalence, mortality, DALYs, and trends of EOCRC and LOCRC due to HBMI [15]. This investigation aims to leverage the GBD 2021 dataset to conduct a systematic assessment of mortality burden and DALYs attributable to EOCRC and LOCRC associated with HBMI across multiple geographical scales, encompassing global, regional, and national levels. Additionally, we evaluated the correlation between EOCRC or LOCRC attributable to HBMI and the SDI, calculated the EAPC to quantify the long-term trends in ASR, and the Nordpred and BAPC models were employed to project the disease burden up to the year 2040. Ultimately, the findings of this investigation are to generate robust epidemiological evidence that can inform evidence-based strategies for early detection, preventive interventions, and health policy formulation pertaining to both EOCRC and LOCRC attributable to HBMI.

## Materials and methods

### Data source and collection

The GBD project is a large-scale multinational research initiative led by the Institute for Health Metrics and Evaluation (IHME). It provides a detailed stratification of population health metrics based on demographic factors such as age, sex, and geographic distribution. The GBD 2021 study offers detailed insights into age- and sex-specific estimates of key health metrics, including incidence, prevalence, deaths, years of life lost (YLLs), years lived with disability (YLDs), and DALYs [16]. DALYs serve as a composite metric that integrates YLDs and YLLs. YLDs are calculated by multiplying the prevalence estimates of disease sequelae, which are stratified by age, sex, geographic region, and time, by their corresponding disability weight coefficients. Similarly, YLLs are determined by multiplying cause-specific mortality data, adjusted for demographic and temporal factors, by the reference standard life expectancy at the age of death [17]. Epidemiologic data on deaths and DALYs for EOCRC and LOCRC attributable to HBMI from 1990 to 2021 were obtained from the Global Health Data Exchange (GHDx) query tool (available at: https://vizhub.healthdata.org/gbd-results/). The data encompassed global estimates, five SDI quintiles, 21 GBD regions, and 204 countries and territories. The searched terms included “Risk factor”, “High body-mass index”, “Colon and rectum cancer”, “Deaths” and “DALYs”, in addition to the years “1990–2021” as well as the metrics “Number,” and “Rate”. SDI values for each country and projected population to 2100 were also obtained from the GBD database.

### SDI definition

The SDI functions as a comprehensive metric that captures the fundamental social and economic factors influencing health outcomes in particular regions. It is primarily calculated using the geometric mean of various indices, each ranging from 0 to 1. These indices include the total fertility rate (TFR) for those under 25 years of age (TFU25), average educational attainment for individuals aged 15 and older (EDU15+), and the lag-distributed income (LDI) calculated on a per capita basis. With the SDI values, the GBD 2021 framework categorizes a total of 204 countries and territories into five specific classifications: low (below 0.45), low-middle (from 0.45 to 0.61), middle (from 0.61 to 0.69), high-middle (from 0.69 to 0.80), and high (0.80 or higher) [14].

### Case identification

CRC are classified as EOCRC or LOCRC based on a diagnostic age threshold of 50 years. This distinction aligns with the recommended starting age for population-based screening programs in many national healthcare systems. It is further supported by differences in clinicopathological characteristics and treatment responses observed between patients diagnosed before and after the age of 50 [4, 18]. Following the coding guidelines outlined in the 9th and 10th editions of the International Classification of Diseases (ICD), colorectal cancer is identified using the following classification codes: ICD-9: 153–154.9, 209.1, 209.5, 211.3–211.4, 230.3–230.6, and 569.0; ICD-10: C18–C21.9, D01.0–D01.3, D12–D12.9, and D37.3–D37.5 [19]. According to GBD 2021 study, HBMI refers to a body mass index of 25 or higher, determined by dividing a person’s weight in kilograms by the square of their height in meters [15].

### Statistical analysis

The ASR of DALYs, along with the EAPC model, were employed to assess the burden and temporal variations of EOCRC and LOCRC attributable to HBMI [20]. The process of standardization is essential when analyzing multiple populations that exhibit diverse age distributions or when examining a single population over time, particularly when its age composition evolves.

The direct standardization method was employed to calculate the ASRs for specific age groups [21]. The ASR per 100,000 population was computed using the following formula:

$$ASR=\frac{\sum_{i=1}^A\left(a_i\times w_i\right)}{\sum_{i=1}^Aw_i}\times100,000$$

Where:

- *a*_*i*_ is the age-specific rate in the *i*^*th*^ age group.
- *w*_*i*_ is the number of persons in the corresponding age group of the selected reference standard population (i.e., the GBD World Standard Population).
- *A* is the number of age groups.
- ∑ represents the sum over all age groups.

This procedure entailed multiplying the rate for each age group by the corresponding proportion of the standard population and subsequently aggregating the results [14]. Importantly, the trends in ASR can serve as dependable markers for the changing patterns of diseases within a specific population, while also offering valuable insights into the evolving risk factors [22].

The EAPC represents a comprehensive and commonly employed metric for assessing the ASR trend across a designated time period [23]. To quantify the temporal trend, a linear regression model was fitted to the natural logarithm of the ASRs. The model is expressed by the equation:

$$\:\text{ln}\left(ASR\right)=\:\alpha\:+\beta\:x+\epsilon\:$$

Where:

- ln(*ASR*) is the natural logarithm of the age-standardized rate.
- *x* is the calendar year.
- *α* is the intercept.
- *β* is the slope coefficient, which represents the average annual change in ln(*ASR*).
- *ε* is the error term.

The EAPC was then derived from the slope coefficient *β* using the formula:

$$\:EAPC=100\:\times\:({e}^{\beta\:}-1)$$

The 95% confidence interval (CI) of the EAPC was calculated from the linear regression model. An upward trend in the ASR was identified when both the EAPC estimate and the lower boundary of its 95% CI were greater than zero. Conversely, a downward trend was determined when both the EAPC estimate and the upper boundary of its 95% CI were below zero. In all other scenarios, the ASR was classified as remaining stable over time.

Additionally, Nordpred and BAPC models [24] were used to predict global DALYs and deaths for EOCRC and LOCRC in 2040. All statistical analyses were conducted utilizing the R programming language (Version 4.3.3). A P-value of less than 0.05 was deemed statistically significant.

## Results

### Global EOCRC and LOCRC burden attributable to HBMI

In 2021, the global burden of EOCRC and LOCRC attributable to HBMI was substantial. The estimated number of DALYs and deaths due to EOCRC were 359,538 (95% UI: 151,562, 567,371) and 7,255 (95% UI: 3,060, 11,453), respectively. For LOCRC, the corresponding number of DALYs and deaths were 2,005,125 (95% UI: 858,739, 3,189,676) and 92,013 (95% UI: 39,256–147,267). Between 1990 and 2021, the burden of EOCRC attributable to HBMI increased significantly. The number of DALYs rose by 130.97%, from 155,666 (95% UI: 61,655, 251,599) to 359,538 (95% UI: 151,562, 567,371). Similarly, the number of deaths more than doubled, increasing by 130.24%, from 3,151 (95% UI: 1,251, 5,096) to 7,255 (95% UI: 3,060, 11,453). A similar upward trend was observed for LOCRC. DALYs increased by 133.32%, rising from 859,377 (95% UI: 363,038, 1,385,148) in 1990 to 2,005,125 (95% UI: 858,739, 3,189,676) in 2021. The number of deaths also grew markedly, with a 139.71% increase from 38,385 (95% UI: 16,175, 62,103) to 92,013 (95% UI: 39,256, 147,267) (Table 1, Additional file 1: Table S1).

**Table 1:** The DALYs and change trends of EOCRC and LOCRC attributable to HBMI from 1990 to 2021

Characteristics	EOCRC					LOCRC				
	1990		2021		1990–2021	1990		2021		1990–2021
	DALYs	ASDR per 100k population	DALYs	ASDR per 100k population	EAPC (%)	DALYs	ASDR per 100k population	DALYs	ASDR per 100k population	EAPC (%)
	NO. (95%UI)	NO. (95%UI)	NO. (95%UI)	NO. (95%UI)	NO. (95%CI)	NO. (95%CI)	NO. (95%UI)	NO. (95%UI)	NO. (95%UI)	NO. (95%CI)
Global										
Both	155,665 (61,654, 251,599)	4.25 (1.69, 6.87)	359,538 (151,562, 567,371)	5.53 (2.33, 8.72)	0.76 (0.69, 0.82)	859,376 (363,038, 1,385,148)	101.81 (42.92, 164.31)	2,005,125 (858,739, 3,189,676)	105.45 (45.12, 167.88)	0.01 (−0.02, 0.05)
Female	74,799 (29,893, 120,964)	4.17 (1.67, 6.73)	155,893 (66,184, 245,091)	4.83 (2.05, 7.59)	0.33 (0.25, 0.41)	450,977 (191,277, 727,987)	98.43 (41.67, 159.01)	939,880 (405,095, 1,487,569)	92.51 (39.87, 146.37)	−0.35 (−0.41, −0.30)
Male	80,866 (31,438, 131,897)	4.33 (1.69, 7.07)	203,646 (86,913, 326,112)	6.22 (2.65, 7.59)	1.12 (1.07, 1.18)	408,399 (168,987, 661,825)	105.57 (43.59, 171.33)	1,065,245 (457,333, 1,699,404)	120.22 (51.57, 191.99)	0.37 (0.34, 0.41)
Socio-demographic index										
High SDI	51,961 (21,682, 83,280)	6.89 (2.87, 11.04)	76,796 (33,564, 119,158)	8.02 (3.5, 12.45)	0.53 (0.48, 0.58)	438,222 (185,894, 707,607)	181.32 (76.89, 292.7)	699,013 (300,250, 1,110,307)	154.59 (66.71, 244.87)	−0.64 (−0.69, −0.59)
High-middle SDI	51,131 (20,342, 83,091)	6.44 (2.57, 10.45)	95,442 (40,243, 154,648)	7.81 (3.29, 12.66)	0.40 (0.26, 0.54)	295,099 (125,414, 475,751)	133.71 (56.71, 215.71)	673,848 (290,683, 1,080,377)	151.81 (65.43, 243.54)	0.29 (0.21, 0.37)
Middle SDI	37,766 (13,767, 62,266)	3.24 (1.19, 5.33)	125,447 (53,282, 199,058)	5.80 (2.46, 9.2)	1.87 (1.79, 1.95)	87,961 (32,871, 143,973)	38.97 (14.53, 63.80)	459,064 (195,170, 736,565)	75.52 (32.09, 121.31)	2.17 (2.14, 2.20)
Low-middle SDI	11,265 (4,208, 18,264)	1.60 (0.60, 2.60)	47,848 (19,743, 76,520)	3.24 (1.34, 5.18)	2.38 (2.31, 2.46)	26,316 (9,985, 42,575)	19.56 (7.39, 31.68)	136,600 (57,045, 216,732)	42.80 (17.86, 68.04)	2.78 (2.71, 2.85)
Low SDI	3,299 (1,162, 5,638)	1.25 (0.44, 2.12)	13,640 (5,417, 21,896)	2.05 (0.82, 3.28)	1.58 (1.45, 1.7)	10,101 (3,679, 17,015)	19.72 (7.10, 33.16)	33,407 (12,953, 54,095)	30.12 (11.6, 48.83)	1.28 (1.16, 1.39)
Regions										
Andean Latin America	941 (377, 1,585)	4.04 (1.63, 6.80)	3,463 (1,534, 5,784)	6.43 (2.85, 10.74)	1.49 (1.34, 1.64)	2,733 (1,103, 4,589)	62.67 (25.21, 105.24)	13,485 (5,806, 22,938)	105.30 (45.28, 179.13)	1.71 (1.59, 1.83)
Australasia	1,566 (632, 2,549)	9.05 (3.66, 14.71)	2,395 (1,047, 3,794)	9.15 (4.00, 14.51)	0.08 (−0.11, 0.27)	11,858 (4,893, 19,046)	234.19 (96.54, 375.97)	21,170 (9,047, 33,915)	181.03 (77.45, 289.19)	−1.09 (−1.18, −1.00)
Caribbean	1,335 (559, 2,156)	5.55 (2.33, 8.96)	3,119 (1,327, 5,180)	8.05 (3.42, 13.36)	1.34 (1.24, 1.44)	4,973 (2,047, 7,961)	89.25 (36.71, 142.93)	17,267 (7,285, 28,346)	145.05 (61.17, 238.11)	1.73 (1.67, 1.79)
Central Asia	2,808 (1,143, 4,570)	7.11 (2.91, 11.56)	4,110 (1,682, 6,731)	5.22 (2.14, 8.54)	−1.00 (−1.15, −0.86)	11,203 (4,641, 18,085)	104.54 (43.25, 168.99)	17,284 (7,404, 27,603)	93.44 (39.98, 149.30)	−0.05 (−0.15, 0.06)
Central Europe	9,598 (4,048, 15,658)	9.41 (3.97, 15.35)	9,607 (4,166, 15,627)	8.60 (3.72, 13.99)	−0.39 (−0.54, −0.24)	77,853 (33,710, 125,780)	231.09 (100.09, 373.42)	139,131 (61,320, 223,352)	285.18 (125.83, 457.35)	0.59 (0.45, 0.72)
Central Latin America	4,056 (1,686, 6,519)	4.04 (1.68, 6.48)	19,522 (8,689, 31,043)	9.20 (4.10, 14.63)	2.75 (2.65, 2.85)	10,418 (4,385, 16,804)	59.45 (24.95, 95.99)	65,014 (28,798, 104,243)	117.32 (51.90, 188.28)	2.21 (2.14, 2.28)
Central Sub-Saharan Africa	290 (0,101, 515)	1.07 (0.38, 1.89)	1,861 (677, 3,325)	2.36 (0.86, 4.21)	2.61 (2.44, 2.79)	1,204 (430, 2,116)	23.52 (8.26, 41.44)	5,777 (2,161, 10,247)	47.41 (17.57, 84.61)	2.30 (2.15, 2.45)
East Asia	34,857 (11,325, 59,382)	3.80 (1.24, 6.46)	98,475 (40,186, 167,252)	7.35 (3.00, 12.45)	2.04 (1.79, 2.28)	79,021 (27,215, 134,247)	41.05 (14.03, 69.79)	430,877 (176,744, 723,153)	85.61 (35.05, 143.60)	2.39 (2.33, 2.46)
Eastern Europe	15,409 (6,436, 24,330)	9.01 (3.77, 14.23)	17,560 (7,342, 27,984)	8.94 (3.73, 14.25)	−0.33 (−0.48, −0.19)	122,501 (52,575, 195,302)	190.53 (81.76, 303.90)	191,164 (82,795, 303,643)	242.98 (105.17, 385.50)	0.57 (0.43, 0.7)
Eastern Sub-Saharan Africa	1,555 (0,532, 2,630)	1.67 (0.58, 2.82)	6,431 (2,351, 10,945)	2.58 (0.95, 4.38)	1.19 (1.09, 1.29)	4,804 (1,692, 8,102)	28.49 (9.90, 48.06)	16,595 (6,253, 27,601)	45.62 (17.04, 75.86)	1.36 (1.26, 1.46)
High-income Asia Pacific	5,997 (2,184, 9,951)	3.73 (1.36, 6.20)	5,841 (2,276, 9,371)	3.40 (1.32, 5.46)	−0.41 (−0.50, −0.32)	30,689 (11,332, 50,175)	68.74 (25.33, 112.44)	74,761 (28,709, 121,776)	76.39 (29.55, 123.91)	0.23 (0.18, 0.28)
High-income North America	21,310 (9,112, 33,872)	8.86 (3.79, 14.07)	36,107 (16,541, 55,175)	12.15 (5.56, 18.57)	1.20 (1.11, 1.29)	163,886 (70,708, 261,967)	217.99 (94.23, 347.69)	260,712 (117,129, 403,462)	183.44 (82.53, 283.25)	−0.72 (−0.82, −0.62)
North Africa and Middle East	11,233 (4,512, 19,001)	5.78 (2.32, 9.76)	38,647 (16,307, 61,619)	7.17 (3.03, 11.44)	0.81 (0.66, 0.97)	28,334 (11,756, 46,090)	76.68 (31.75, 124.81)	117,836 (50,526, 186,688)	120.19 (51.56, 191.02)	1.61 (1.47, 1.75)
Oceania	149 (60, 255)	3.74 (1.51, 6.41)	405 (167, 684)	4.02 (1.66, 6.78)	0.18 (0.12, 0.24)	322 (131, 0,547)	48.93 (19.76, 83.43)	957 (406, 1,554)	56.58 (23.91, 91.95)	0.52 (0.44, 0.59)
South Asia	5,738 (1,962, 9,514)	0.84 (0.29, 1.39)	25,421 (9,675, 41,127)	1.72 (0.66, 2.78)	2.32 (2.26, 2.38)	10,956 (3,467, 18,669)	8.32 (2.61, 14.19)	66,079 (25,648, 106,634)	19.81 (7.68, 32.04)	2.88 (2.82, 2.93)
Southeast Asia	7,164 (2,599, 11,831)	2.42 (0.88, 3.99)	30,291 (12,228, 49,574)	4.93 (1.99, 8.06)	2.29 (2.12, 2.46)	13,789 (4,808, 22,676)	23.68 (8.13, 39.07)	89,050 (36,237, 147,423)	58.63 (23.70, 97.15)	3.03 (2.91, 3.15)
Southern Latin America	2,832 (1,185, 4,721)	7.74 (3.24, 12.90)	6,254 (2,758, 10,203)	10.70 (4.71, 17.46)	1.40 (1.26, 1.53)	19,240 (8,167, 31,718)	189.89 (80.58, 313.22)	43,915 (19,213, 72,271)	230.52 (100.97, 379.22)	0.88 (0.72, 1.05)
Southern Sub-Saharan Africa	1,700 (690, 2,692)	5.44 (2.22, 8.60)	5,106 (2,077, 8,179)	7.93 (3.23, 12.70)	1.69 (1.34, 2.05)	3,788 (1,556, 6,137)	66.60 (27.26, 108.32)	16,809 (7,106, 26,646)	135.34 (57.04, 214.83)	2.52 (2.22, 2.81)
Tropical Latin America	4,837 (1,950, 7,895)	4.69 (1.90, 7.65)	19,040 (8,140, 29,987)	9.25 (3.95, 14.56)	1.95 (1.82, 2.08)	14,504 (5,925, 23,583)	73.67 (29.93, 119.84)	75,264 (32,045, 120,431)	132.06 (56.18, 211.42)	1.88 (1.77, 1.98)
Western Europe	21,144 (8,643, 34,803)	6.55 (2.68, 10.78)	20,437 (8,819, 33,388)	5.48 (2.36, 8.94)	−0.58 (−0.65, −0.51)	243,136 (102,253, 393,869)	191.07 (80.32, 309.71)	324,539 (136,878, 531,803)	159.48 (67.51, 260.69)	−0.68 (−0.75, −0.61)
Western Sub-Saharan Africa	1,147 (446, 1,879)	1.13 (0.44, 1.85)	5,447 (2,045, 9,215)	1.99 (0.75, 3.36)	1.91 (1.86, 1.95)	4,164 (1,610, 6,830)	22.10 (8.51, 36.24)	17,438 (6,906, 28,575)	42.33 (16.72, 69.29)	2.25 (2.19, 2.31)

*DALYs *Disability-adjusted life years, *EOCRC* Early-onset colorectal cancer, *LOCRC *Late-onset colorectal cancer, *BMI* Body mass index, *ASD*R Age-standardized disability-adjusted life years rate, *EAPC* Estimated annual percentage change

**Fig. 1:**
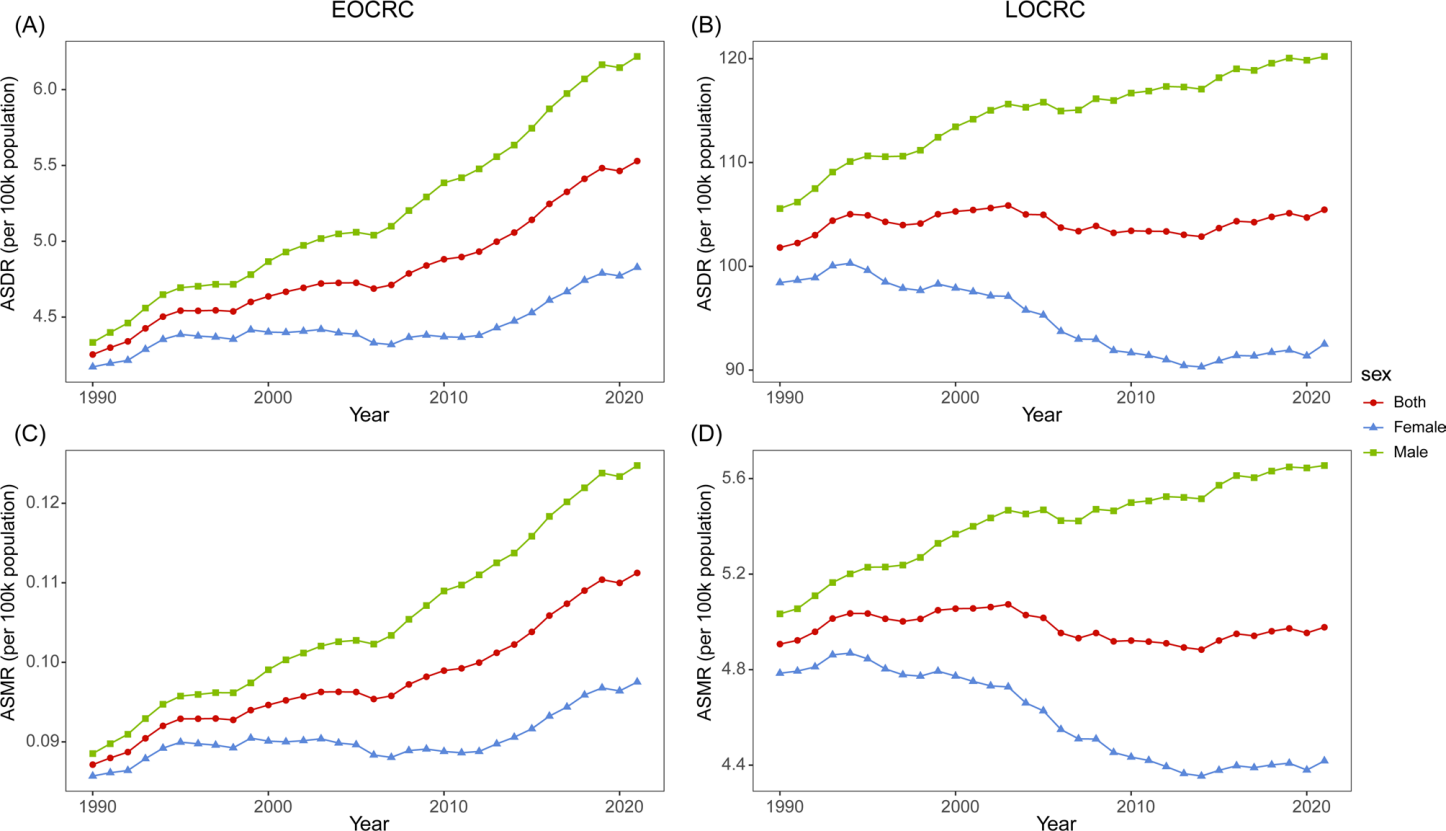
Global changes in the ASDR and ASMR in EOCRC (**A**, **C**) and LOCRC (**B**, **D**) attributable HBMI. ASDR, age-standardized disability-adjusted life years rate; ASMR, age-standardized mortality rate; EOCRC, early-onset colorectal cancer; LOCRC late-onset colorectal cancer; BMI, body mass index

The ASDR of EOCRC and LOCRC attributable to HBMI was 5.53 (95% UI: 2.33, 8.72) and 105.45 (95% UI: 45.12, 167.88) per 100k population, respectively. The corresponding ASMR were 0.11 (95% UI: 0.04, 0.18) and 4.98 (95% UI: 2.12, 7.98) per 100k population (Table 1, Additional file 1: Table S1). Between 1990 and 2021, the global ASDR and ASMR of EOCRC attributable to HBMI exhibited an increasing trend in both males and females. The EAPC for ASDR was 0.76 (0.69, 0.82), while for ASMR, it was 0.70 (95% CI: 0.63, 0.76) (Fig. 1A, C). In contrast, for LOCRC, the global ASDR remained relatively stable, whereas the ASMR demonstrated a slight decline, with EAPCs of 0.01 (95% CI: −0.02, 0.05) and − 0.05 (95% CI: −0.09, −0.01), respectively. Subgroup analysis by gender revealed divergent trends. Among females, both ASDR and ASMR showed a decreasing pattern, with EAPCs of −0.35 (95% CI: −0.41, −0.30) and − 0.41 (95% CI: −0.46, −0.36), respectively. In contrast, males exhibited a persistent upward trend, with EAPCs of 0.37 (95% CI: 0.34, 0.41) for ASDR and 0.34 (95% CI: 0.31, 0.38) for ASMR (Fig. 1B, D).

### EOCRC and LOCRC burden attributable to HBMI among SDI regions

**Fig. 2:**
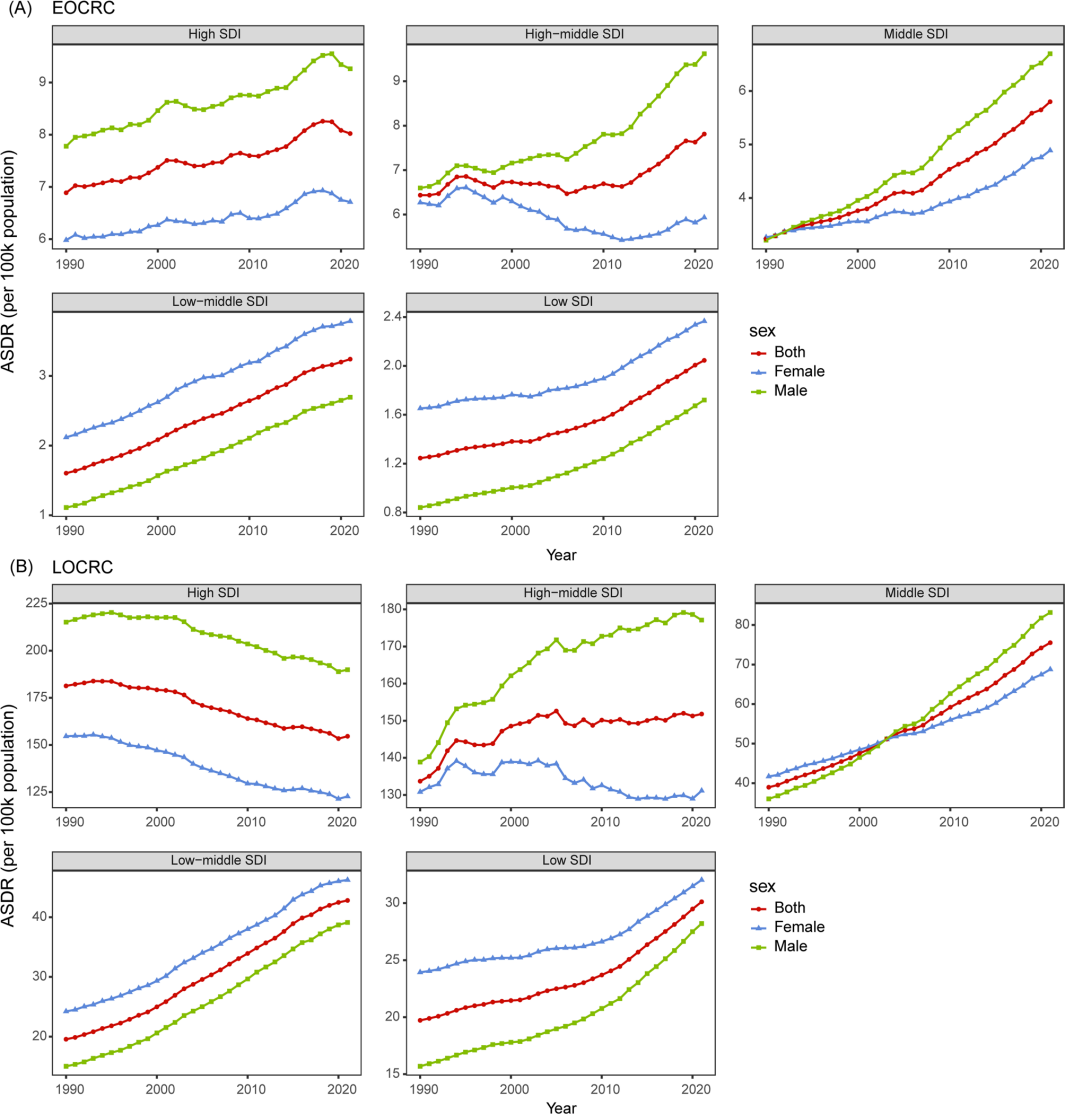
The trends of ASDR in EOCRC (**A**) and LOCRC (**B**) attributable HBMI in SDI regions. ASDR, age-standardized disability-adjusted life years rate; EOCRC, early-onset colorectal cancer; LOCRC, late-onset colorectal cancer; BMI, body mass index; SDI, sociodemographic index

In 2021, the highest number of DALYs for EOCRC and LOCRC attributable to HBMI was observed in middle and high SDI regions, respectively. Middle SDI regions recorded 125,447 DALYs (95% UI: 53,282, 199,058) for EOCRC, while high SDI regions had 699,013 DALYs (95% UI: 300,250, 1,110,307) for LOCRC. In contrast, the lowest number of DALYs was reported in low SDI regions, with 13,640 (95% UI: 5,417, 21,896) for EOCRC and 33,407 (95% UI: 12,953, 54,095) for LOCRC (Table 1). Furthermore, the distribution of mortality for EOCRC and LOCRC mirrored the regional patterns of DALYs across SDI categories (Additional file 1: Table S1). Age-standardized results indicate that high SDI regions had the highest ASDR and ASMR for both EOCRC and LOCRC attributable to HBMI, while low SDI regions had the lowest rates in 2021 (Table 1, Additional file 1: Table S1). From 1990 to 2021, ASDR and ASMR generally increased across most SDI regions for both EOCRC and LOCRC. However, an exception was observed in high SDI regions for LOCRC, where both ASDR and ASMR declined, with EAPCs of −0.64 (95% CI: −0.69, −0.59) and − 0.72 (95% CI: −0.77, −0.66), respectively. Sex-specific analysis in high SDI regions showed a consistent decline in ASDR and ASMR for LOCRC attributable to HBMI in both males and females, with EAPCs of −0.51 (95% CI: −0.57, −0.45) and − 0.57 (95% CI: −0.63, −0.52) for ASDR, and − 0.89 (95% CI: −0.95, −0.84) and − 0.97 (95% CI: −1.03, −0.90) for ASMR. Additionally, the declining trends in ASDR and ASMR among females in high-middle SDI regions were independent of tumor onset time (Fig. 2, Additional file 2: Figure S1-S2).

### EOCRC and LOCRC burden attributable to HBMI among regions

Geographically, in 2021, East Asia recorded the highest number of DALYs and deaths in both EOCRC [98,475 (95% UI: 40,186, 167,252) and 430,877 (95% UI: 176,744–723,153)] and LOCRC [1,965 (95% UI: 803, 3,342) and 18,406 (95% UI: 7,530, 30,739)] attributable to HBMI. In EOCRC, the highest ASDR was observed in High-income North America, and the highest ASMR was found in North Africa and the Middle East, with values of [12.15 (95% UI: 5.56, 18.57) and 0.24 (95% UI: 0.11, 0.37) per 100k population], respectively. In LOCRC, Central Europe recorded the highest ASDR and ASMR, reaching [285.18 (95% UI: 125.83, 457.35) and 13.24 (95% UI: 5.80, 21.30) per 100k population], followed by Eastern Europe and Southern Latin America. South Asia exhibited the lowest ASDR and ASMR in both EOCRC and LOCRC (Table 1, Additional file 1: Table S1).

**Fig. 3:**
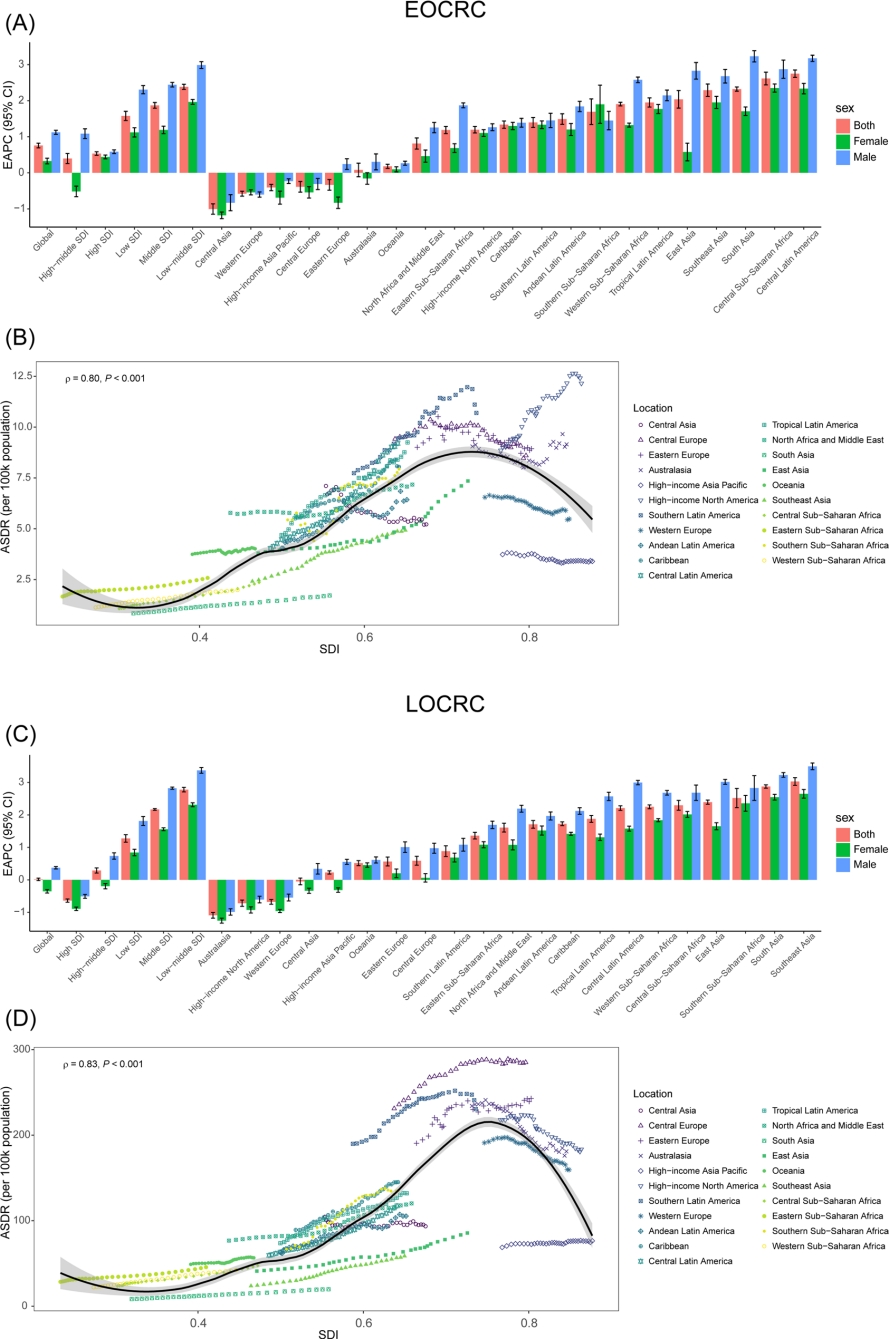
EAPC of ASDR (**A**, **C**) and correlation between ASDR and SDI (**B**, **D**) in 21 GBD regions between 1990 and 2021 in EOCRC and LOCRC attributable HBMI. ASDR, age-standardized disability-adjusted life years rate; EAPC, estimated annual percentage change; EOCRC, early-onset colorectal cancer; BMI, body mass index; SDI, sociodemographic index; GBD, global burden disease

Between 1990 and 2021, Central Latin America had the largest increases and Central Asia had the largest decreases in ASDR [EAPC: 2.75 (95% CI: 2.65, 2.85) vs. −1.00 (95% CI: −1.15, −0.86)), respectively] (Table 1; Fig. 3A) and ASMR [EAPC: 2.71 (95% CI: 2.61, 2.81) vs. −1.03 (95% CI: −1.17, 0.89)), respectively] (Additional file 1: Table S1, Additional file 2: Figure S3) in EOCRC attributable to HBMI. Southeast Asia had the largest increases and Australasia had the largest decreases in ASDR [EAPC: 3.03 (95% CI: 2.91, 3.15) vs. −1.09 (95% CI: −1.18, −1.00), respectively] (Table 1; Fig. 3C) and ASMR [EAPC: 3.17 (95% CI: 3.06, 3.27) vs. −0.83 (95% CI: −0.91, −0.75), respectively] (Additional file 1: Table S1, Additional file 2: Figure S4) in LOCRC attributable to HBMI. Overall. a nonlinear short-tailed “S”-shaped relationship was observed between the ASDR or ASMR and the SDI for both EOCRC and LOCRC attributable to HBMI. This association followed a variable pattern, initially decreasing, then increasing, before ultimately declining again.

### EOCRC and LOCRC burden attribute to HBMI by countries

In 2021, China recorded the largest number of DALYs for both EOCRC and LOCRC attributable to HBMI, with 95,334 (95% UI: 38,733, 162,578) and 411,982 (95% UI: 169,014, 692,218), respectively. The United States followed, reporting 33,332 (95% UI: 15,301, 50,773) DALYs for EOCRC and 234,963 (95% UI: 105,817, 362,953) for LOCRC. Brazil ranked third in EOCRC with 18,692 (95% UI: 7,987–29,424) DALYs, while the Russian Federation had the third-highest burden for LOCRC, recording 135,067 (95% UI: 57,678–213,076) DALYs. In terms of mortality, the distribution of deaths caused by LOCRC was consistent with DALYs, while for EOCRC, India replaced Russia as the third highest country (Additional file 1: Table S2-S3).

**Fig. 4:**
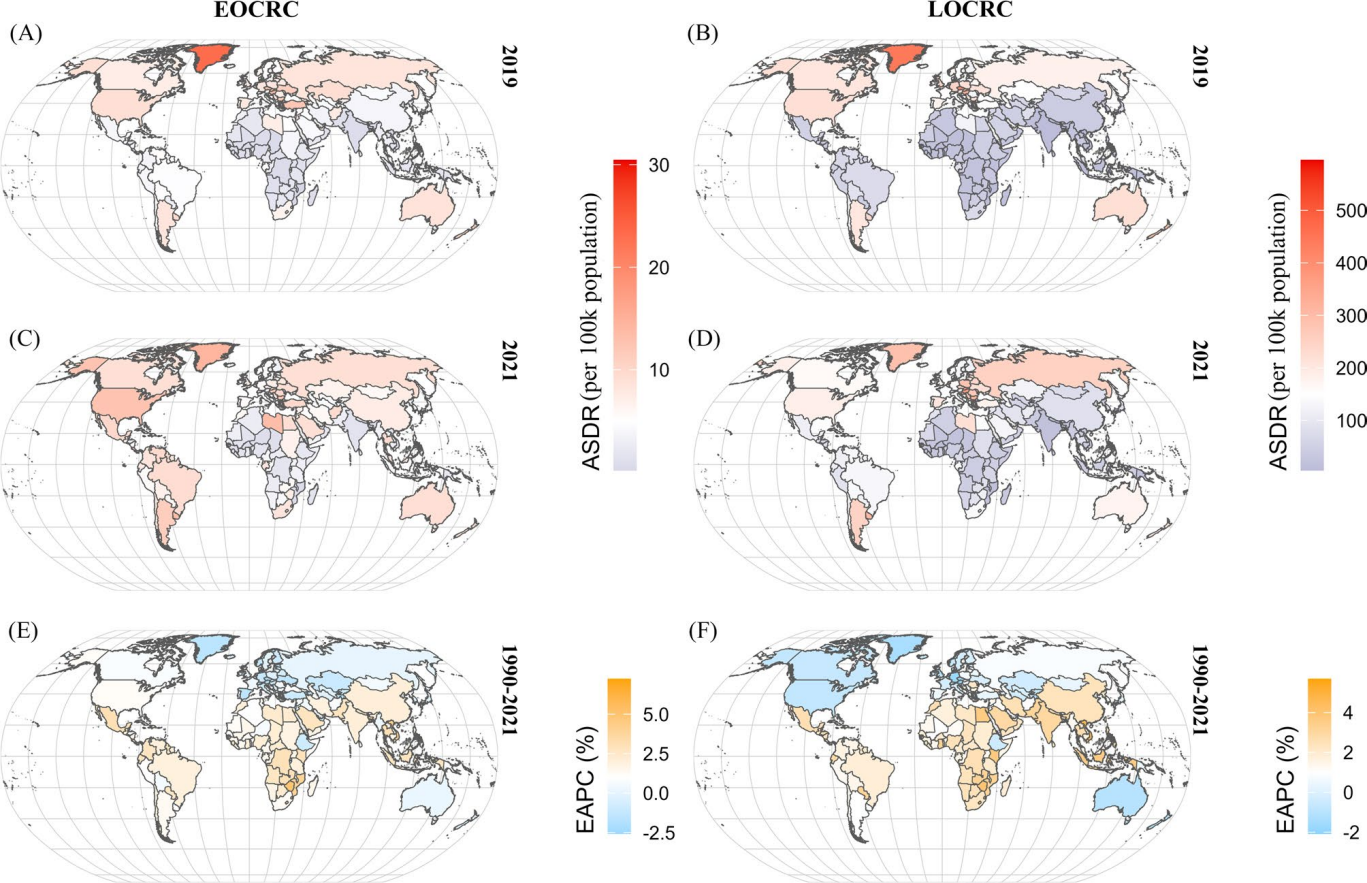
Spatial distribution of the ASDR and EAPC between 2019 and 2021 in EOCRC (**A**, **C**, **E**) and LOCRC (**B**, **D**, **F**) attributable to HBMI. ASDR, age-standardized disability-adjusted life years rate; EAPC, estimated annual percentage change; EOCRC, early-onset colorectal cancer; LOCRC, late-onset colorectal cancer; BMI, body mass index

Similarly, in 2021, the country with the highest ASDR for EOCRC was Nauru [26.52 (95% UI: 9.86, 48.11)], followed by American Samoa and the Bahamas [21.11 (95% UI: 9.53, 35.81) and 17.32 (95% UI: 6.93, 28.99), respectively]. The highest ASDR for LOCRC was observed in Hungary [377.55 (95% UI: 168.71, 614.92)], followed by Slovakia and Bulgaria [344.13 (95% UI: 149.73, 578.30) and 321.59 (95% UI: 136.19, 544.81), respectively] (Figs. 4C, D, Additional file 1: Table S2). Furthermore, the spatial distribution of deaths and ASMR for both EOCRC (Additional file 2: Figures S7) and LOCRC (Additional file 2: Figures S8) attributable to HBMI was similar to that of DALYs and ASDR (Additional file 1: Table S3).

Lesotho, Zimbabwe, and Viet Nam had the highest increases in ASDR in EOCRC attributable to HBMI, with EAPCs of 5.99 (95% CI: 5.28, 6.70), 4.91 (95% CI: 3.89, 5.94), and 4.39 (95% CI: 4.10, 4.68), respectively. Similarly, Viet Nam, Lesotho, and Cabo Verde had the highest increases in ASDR in LOCRC attributable to HBMI, with EAPCs of 4.81 (95% CI: 4.62, 5.01), 4.50 (95% CI: 3.97, 5.04), and 4.21 (95% CI: 3.83, 4.60), respectively. Meanwhile, Luxembourg and Austria experienced the fastest decline in ASDR in EOCRC and LOCRC attributable to HBMI, with EAPCs of −2.38 (95% CI: −2.61, −2.16) and − 1.89 (95% CI: −1.95, −1.82), respectively (Figs. 4E, F, Additional file 1: Table S2). Furthermore, the changes in ASMR mirrored the trends observed in ASDR for both EOCRC (Additional file 2: Figures S9) and LOCRC (Additional file 2: Figures S10) attributable to HBMI (Additional file 1: Table S3).

### Prediction of global burden

**Fig. 5:**
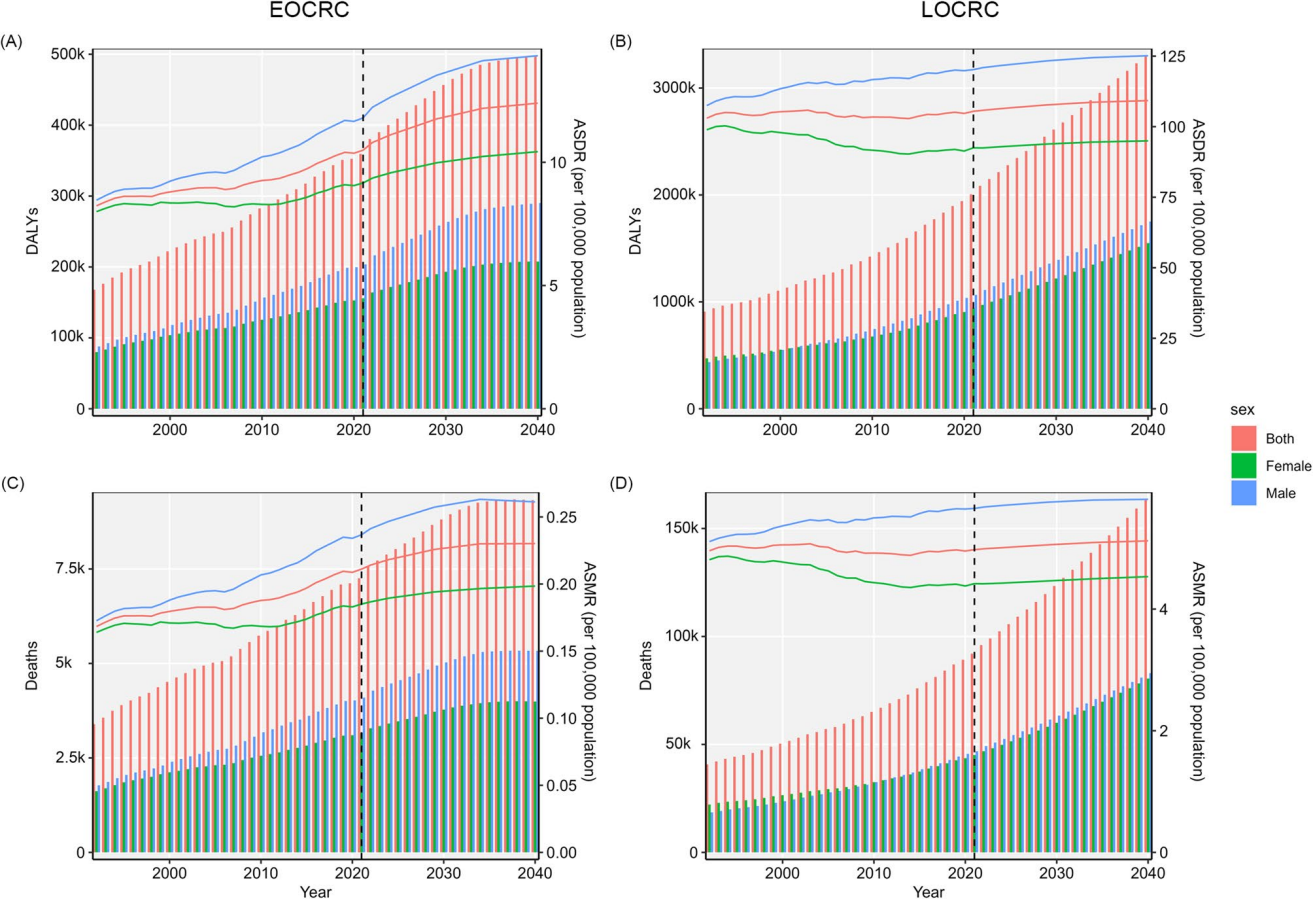
The predicted DALYs, deaths, ASDR and ASMR in EOCRC (**A**, **C**) and LOCRC (**B**, **D**) attributable to HBMI from 2021 to 2040 based on Nordpred model. DALYs, disability-adjusted life years; BMI, body mass index; ASDR, age-standardized DALYs rate; ASMR, age-standardized mortality rate; EOCRC, early-onset colorectal cancer; LOCRC, late-onset colorectal cancer

To investigate the DALYs and deaths attributable to ECRC and LOCRC through 2040 and support public health prevention strategies, the Nordpred and BAPC models were employed. Based on the Nordpred analysis, DALYs increased 1.38-fold in EOCRC and 1.65-fold in LOCRC from 2021 to 2040 (359,539 to 497,919, 2,005,125 to 3,302,310, respectively). Number of deaths increased 2.75-fold in EOCRC and 4.02-fold in LOCRC from 2021 to 2040 (3,392 to 9,325, 40,704 to 163,487, respectively). The ASDR increased from 10.49 to 105.45 in 2021 to 12.40 and 109.24 in 2040; the ASMR increased from 0.21 to 4.98 in 2021 to 0.23 and 5.12 in 2040 for EOCRC and LOCRC attributable to HBMI, respectively. And the ASDR and ASMR among men being consistently higher than that among women both EOCRC and LOCRC attributable to HBMI (Fig. 5; Additional file 1: Table S4-S5).

**Fig. 6:**
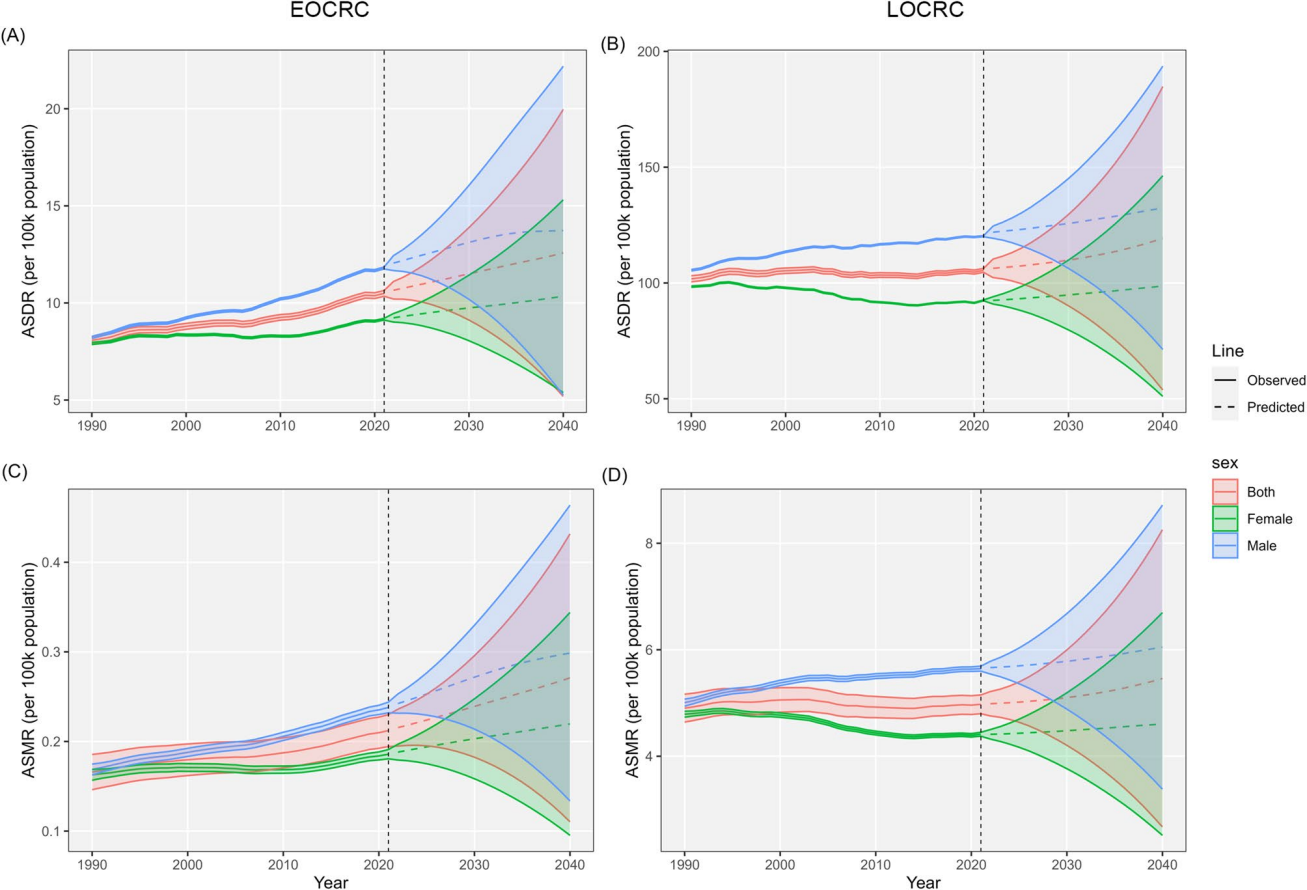
The predicted DALYs, deaths, ASDR and ASMR in EOCRC (**A**, **B**) and LOCRC (**C**, **D**) attributable to HBMI from 2021 to 2040 based on BAPC model. DALYs, disability-adjusted life years; BMI, body mass index; ASDR, age-standardized DALYs rate; ASMR, age-standardized mortality rate; EOCRC, early-onset colorectal cancer; LOCRC, late-onset colorectal cancer

The prediction model based on BAPC also yielded similar results, with the number of DALYs increasing from 350,815 and 1,897,093 in 2021 to 496,593 and 3,377,296 in 2040 for EOCRC and LOCRC, respectively, representing an estimated increase of 41.55% and 78.02%. The number of deaths increased from 7,053 to 86,990 in 2021 to 10,721 and 162,306 in 2040, with estimated increases of 52.01% and 86.58%, respectively. After age standardization, the ASDR increased from 10.40 to 105.11 in 2021 to 12.34 and 116.78 in 2040, while the ASMR increased from 0.21 to 4.97 in 2021 to 0.26 and 5.36 for EOCRC and LOCRC attributable to HBMI, respectively. Analysis of subgroup predictions by sex indicated an upward trend in the burden for both genders. Furthermore, males consistently exhibited greater counts of DALYs, mortality, and ASR than females (Fig. 6; Additional file 1: Table S6-S7).

## Discussion

This investigation employed the most recent GBD 2021 data to elucidate the epidemiological features of the worldwide burden of EOCRC and LOCRC attributable to HBMI. The study results indicate that, over the course of the last three decades, the absolute numbers of global deaths and DALYs associated with HBMI-related EOCRC and LOCRC have more than doubled. The ASMR and ASDR of global EOCRC have shown rapid growth; in contrast, the ASMR and ASDR of LOCRC have remained relatively stable, with a declining trend observed in females. The rising trend of global HBMI-related EOCRC and LOCRC burden can be partially explained by the increasing prevalence of HBMI, alongside the growth and aging of the global population [25, 26]. Despite considerable progress in comprehending the pathophysiology of obesity and its related health challenges, HBMI has continued to increase globally in recent years [6, 7, 27].

Our analysis reveals a critical divergence: while the burden of LOCRC attributable to HBMI has begun to decline in high-SDI regions, the burden of EOCRC continues to rise across all SDI strata. This disparity suggests distinct underlying mechanisms and drivers beyond mere HBMI exposure duration. For LOCRC in high-SDI regions, the observed decline is likely multifactorial, attributable to the successful implementation of population-based screening programs after the age of 50, improved access to advanced treatments, and greater public awareness of CRC risks and healthy lifestyles [28]. Conversely, the universal increase in EOCRC highlights a significant gap in current preventive strategies. Younger individuals fall outside the age range of routine screening, and the aggressive biology of EOCRC often leads to delayed diagnosis and poorer outcomes [29]. The rising HBMI prevalence among younger global populations may be a key driver, but it likely interacts with other early-life exposures that are not yet fully captured or mitigated by public health policies targeting older adults [30]. This underscores an urgent need for targeted research into the unique etiology of EOCRC and the development of age-specific prevention protocols.

Furthermore, the global incidence and mortality of CRC attributable to HBMI have exhibited a marked increase, with significant disparities observed across age groups, genders, and sociodemographic index regions [31]. This is consistent with our analysis, which shows that the overall DALYs and mortality of EOCRC have increased across all SDI regions, whereas gender-based subgroup analysis reveals a declining burden among females in High-middle SDI regions. For LOCRC, the overall burden in High SDI districts has shown a gradual decline, with subgroup analysis indicating a decreasing trend in both genders in High SDI districts and among females in High Middle SDI districts, while other SDI regions and genders continue to experience an increasing trend. The annual increase in CRC mortality and DALYs in low-to-middle SDI territories, contrasted with a decreasing trend in high SDI territories, may be attributed to the more comprehensive screening programs and better healthcare resources available in high SDI regions compared to lower SDI regions [32, 33].

The observed gender differences may be attributed to greater health awareness, the widespread implementation of early screening programs, and the protective role of hormone-related factors in females. Moreover, women may respond more effectively to interventions such as dietary changes and weight management, especially in middle-to-high SDI regions [34, 35]. In low-to-middle SDI regions, males exhibit a higher burden of HBMI-related CRC, this may be explained by a combination of behavioral, biological, and healthcare-access factors. Men tend to have a higher prevalence of visceral adiposity, which is more strongly associated with insulin resistance and pro-inflammatory states than subcutaneous fat [36]. Furthermore, lifestyle factors such as higher rates of smoking and alcohol consumption, coupled with lower health-seeking behavior and participation in screening programs, likely contribute to the increased burden observed in males [37]. Genetic and molecular studies have also shown that male CRC patients have a higher N-ras gene mutation rate and other unfavorable molecular features, potentially worsening disease severity [38, 39].

At the GBD regionals, East Asia exhibits the highest DALYs and mortality for both EOCRC and LOCRC, which may be influenced by various factors. First, with the improvement in living standards coupled with shifts in dietary patterns has resulted in a gradual increase in obesity rates throughout East Asia; this trend may significantly contribute to the escalating burden of CRC attributable to elevated BMI. Moreover, research indicates that the correlation between HBMI and CRC risk is more pronounced in Asian populations, which may be attributed to differences in body composition, fat distribution, and muscle mass, as Asians tend to have a higher body fat percentage at the same BMI level [40–42]. Since the dawn of the 21 st century, the rapid economic advancement and industrialization within Asia have instigated lifestyle alterations, including the adoption of Western dietary practices characterized by increased consumption of fats and red meats, alongside decreased physical activity and rising obesity prevalence [43, 44]. Furthermore, genetic predisposition might also play a role in this phenomenon. Although the precise mechanisms are not yet fully understood, research indicates that certain genetic variants in specific populations, such as East Asians, may increase their susceptibility to HBMI-related colorectal cancer [45]. The highest incidence of EOCRC in High-income North America and LOCRC in Central Europe may be attributed to factors including lifestyle, dietary patterns, healthcare resource distribution, and screening coverage in these regions [46, 47]. The minimal CRC burden in South Asia may be attributed to the traditional South Asian diet, which mainly consists of plant-based foods, has relatively low fat content, and includes a high proportion of fiber-rich foods, potentially lowering colorectal cancer risk [48]; Among low-income populations, the prevalence of obesity tends to be lower, which consequently diminishes the impact of BMI on CRC risk [49].

Our research further revealed that the DALY rate and mortality of both EOCRC and LOCRC attributable to HBMI show a nonlinear S-shaped trend with SDI variations, characterized by an initial decline, followed by an increase, and a subsequent decline again. This trend reflects the complex interactions of social, economic, and health behavior changes. In regions with low SDI, the burden of CRC attributable to HBMI is substantial due to the lack of healthcare facilities, shortage of medical personnel, low health awareness, and inadequate preventive healthcare measures [50–52]. As SDI improves, advancements in public health conditions and better healthcare services contribute to a modest decline in disease burden. With further increases in SDI, the Westernization of lifestyles, including changes in dietary patterns with higher caloric intake and reduced physical activity, exacerbates obesity, thereby elevating the risk of CRC attributable to HBMI, which results in rising DALYs and mortality [47]. In high SDI regions, a stronger focus on health management, proactive preventive interventions, and superior treatment strategies likely contribute to the reduction of CRC burden attributable to HBMI, resulting in a final decline in DALYs and mortality [53, 54]. More in-depth studies targeting specific populations and regions are needed to better understand this phenomenon and uncover its underlying causes.

At the national scale, China, recognized as the most populous nation globally [55], experiences the highest incidence of mortality and DALYs related to EOCRC and LOCRC associated with HBMI. Multiple studies have shown that changes in dietary patterns and lifestyle habits, characterized by increased consumption of animal-derived foods, processed grains, highly processed items, and a growing prevalence of sedentary behaviors, have contributed to this phenomenon. This has resulted in a persistent rise in the rates of HBMI among Chinese adolescents and adults [56–58], consequently escalating the burden of CRC attributable to HBMI [59]. Colonoscopy is widely acknowledged as the gold standard for intestinal tumor screening, Nevertheless, the absence of a nationwide screening program and limited healthcare resources have prevented the implementation of population-based CRC screening in China [60].

From a clinical standpoint, our findings underscore the urgent need for tailored screening strategies that account for both age and BMI. Given the rising burden of EOCRC attributable to high BMI, particularly in younger populations who fall outside conventional screening guidelines, clinicians should consider advocating for earlier colorectal cancer screening in individuals with elevated BMI—especially those with additional risk factors such as family history, metabolic syndrome, or sedentary lifestyle. While current guidelines typically recommend initiating screening at age 45–50 for average-risk individuals [61], our data suggest that obesity may warrant a more aggressive approach, such as initiating colonoscopy at an earlier age or using non-invasive tests and AI-assisted algorithms as a first-step risk stratification and diagnosis [62]. Further research is needed to establish evidence-based, BMI-stratified screening protocols that balance benefits, harms, and resource allocation, particularly in regions with high obesity prevalence.

Notwithstanding its comprehensive scope, the present study is subject to several limitations inherent in the GBD framework and our analytical approach. First, our findings rely on GBD 2021 modeled estimates rather than raw registry data. Although considerable efforts have been made to enhance data quality and comparability across regions, residual inaccuracies are inevitable, particularly for EOCRC in data-sparse settings. Additionally, The 2040 burden projections presented in the main text lack UIs, which may impart a misleading impression of precision. It is important to note that these UIs were not available in the underlying GBD modeling output for such long-term projections, and consequently, could not be included in this study or its supplementary materials. We urge readers to interpret these projected estimates with caution and regard them as indicative of potential trends rather than precise numerical predictions. Second, the GBD dataset aggregates colon and rectal cancers, preventing subtype-specific attribution of burden to HBMI. This is a significant constraint given emerging evidence of potential differences in their etiology and risk factor profiles. Third, the use of a uniform BMI threshold (≥ 25 kg/m²) to define HBMI presents conceptual limitations. This metric does not capture variations in body composition, fat distribution, or ethnicity-specific risk—such as the elevated health risks observed in Asian populations at lower BMI levels. Moreover, BMI likely interacts with other genetic, metabolic, and environmental factors in the multifactorial process of colorectal carcinogenesis, which cannot be fully disentangled in this analysis. Fourth, as a secondary analysis of existing GBD data, our study is bound by the imputation models and methodological assumptions of the IHME, which cannot be modified or independently validated. This includes the handling of “garbage codes”—implausible or insufficiently specific cause-of-death assignments—through IHME’s redistribution algorithms [63]. Although these methods aim to enhance comparability across different vital registration systems, the reassignment of garbage-coded deaths may influence the accuracy of cause-specific mortality estimates, including those for colorectal cancer. We encourage readers to consider how these adjustments might affect the estimated burden attributed to HBMI, particularly in regions with lower-quality cause-of-death data. In light of these considerations, we emphasize that the findings of this study should be interpreted as broad, model-driven estimates that provide a systematic—yet inevitably incomplete—perspective on the future burden of colorectal cancer attributable to high BMI.

In summary, the increasing global burden of EOCRC and LOCRC attributable to HBMI highlights the substantial strain on healthcare systems worldwide. HBMI is disproportionately prevalent in economically developed nations while also increasing across various population groups. This rising trend is particularly concerning in resource-limited settings, where access to adequate oncological care remains insufficient. Despite robust evidence indicating a correlation between regular physical exercise, weight loss, and a diminished risk of disease, along with improved survival rates across multiple cancer forms, the implementation of effective weight management strategies aimed at cancer prevention remains limited in clinical settings. Consequently, a collaborative effort from government entities, civic organization healthcare professionals, and individuals is needed to enhance public awareness of the health risks associated with HBMI, promote balanced nutrition and regular physical activity, and improve access to medical care for HBMI populations, ultimately aiming to alleviate the CRC burden associated with HBMI.

## Conclusion

Over the past three decades (1990–2021), the burden of LOCRC attributable to HBMI remains significantly high and consistently exceeds that of EOCRC. However, the rapid increase in EOCRC warrants particular attention. The disease burden of both EOCRC and LOCRC due to HBMI is closely associated with the SDI. As SDI increases, the burden of disease has exhibited a fluctuating pattern—initially declining, then rising, and subsequently decreasing again. In high-SDI regions, the burden of LOCRC has begun to decline, whereas EOCRC continues to increase regardless of SDI levels. Over the next two decades, the burden of both EOCRC and LOCRC attributable to HBMI is projected to continue rising. To address this growing public health challenge, increased economic investment and the implementation of targeted health policies are crucial in mitigating the substantial and escalating global burden of colorectal cancer attributable to HBMI.

## Supplementary Information

Supplementary Material 1

Supplementary Material 2

## Data Availability

The datasets presented in this study can be found in the article/Supplementary Material. Further inquiries can be directed to the corresponding author.

## Abbreviations

CRC: Colorectal cancer
EOCRC: Early-onset CRC
LOCRC: Late-onset CRC
HBMI: High body mass index
GBD: Global Burden of Disease Study
EAPC: Estimated annual percentage change
ASR: Age-standardized rate
DALYs: Disability-adjusted life years
ASDR: Age-standardized DALYs rate
ASMR: Age-standardized mortality rate
SDI: Socio-demographic index
IHME: Institute for Health Metrics and Evaluation
YLLs: Years of life lost
YLDs: Years lived with disability
GHDx: Global Health Data Exchange
TFR: Total fertility rate
EDU15+: Educational attainment for individuals aged 15 and older
LDI: Lag-distributed income
ICD: International Classification of Diseases
UI: Uncertainty interval
CI: Confidence interval
