# Supplementary material for: Global, regional, and national burden of early-onset and late-onset colorectal cancer attributable to high body-mass index from 1990 to 2021: a trend analysis and forecasts up to 2040 based on the global burden of disease study 2021
Source: BMC Gastroenterol. 2025 Nov 19;25:816. doi: 10.1186/s12876-025-04432-7 (PMC12629028; doi:10.1186/s12876-025-04432-7)
Supplement: Supplementary file 1 — Supplementary Material 1 [file 12876_2025_4432_MOESM1_ESM.docx]

**Supplementary Tables**

**Global, regional, and national burden of early-onset and late-onset colorectal cancer attributable to high body-mass index from 1990 to 2021: A trend analysis and forecasts up to 2040 based on the Global Burden of Disease Study 2021**

Bin Yue ^1^ *, Zhongqiao Lu ^2^, Desan Zong ^1^,Yingxia Hu ^1^, Zhongde Yang ^1^

^1^ Department of Gastroenterology, The People’s Hospital of Wenshan Prefecture; Affiliated Wenshan Hospital, KunMing University of Science and Technology, Wenshan 663000, Yunnan Province, China

^2^ Department of Cardiac and Vascular diseases, The People’s Hospital of Wenshan Prefecture; Affiliated Wenshan Hospital, KunMing University of Science and Technology, Wenshan 663000, Yunnan Province, China

*** Corresponding:**

Bin Yue

Department of Gastroenterology, The People’s Hospital of Wenshan Prefecture; Affiliated Wenshan Hospital, KunMing University of Science and Technology, Wenshan 663000, Yunnan Province, China.

Address: No. 31, Tenglong North Road, Wenshan, 663000, Yunnan, China.

Email: helloybin@163.com

Tel: +86 15393954526

**Contents**

[Table S1. The number of deaths and change trends of EOCRC and LOCRC attributable to high BMI from 1990 to 2021 1](#_Toc190435999)

[Table S2 DALYs, ASDR and EAPC in 204 countries in EOCRC and LOCRC attributable to HBMI in 2021 4](#_Toc190436000)

[Table S3 Deaths, ASMR and EAPC in 204 countries in EOCRC and LOCRC attributable to HBMI in 2021 15](#_Toc190436001)

[Table S4 the predicted DALYs and ASDR of EOCRC and LOCRC attributable to high BMI from 2021 to 2040 based on Nordpred model 25](#_Toc190436002)

[Table S5 the predicted Deaths and ASMR of EOCRC and LOCRC attributable to HBMI from 2021 to 2040 based on Nordpred model 27](#_Toc190436003)

[Table S6 the predicted DALYs and ASDR of EOCRC and LOCRC attributable to high BMI from 2021 to 2040 based on BAPC model 29](#_Toc190436004)

[Table S7 the predicted Deaths and ASMR of EOCRC and LOCRC attributable to HBMI from 2021 to 2040 based on BAPC model 31](#_Toc190436005)

# Table S1. The number of deaths and change trends of EOCRC and LOCRC attributable to high BMI from 1990 to 2021

| **Characteristics** | **EOCRC** | | | | | **EOCRC** | | | | |
| --- | --- | --- | --- | --- | --- | --- | --- | --- | --- | --- |
|  | 1990 | | 2021 | | 1990-2021 | 1990 | | 2021 | | 1990-2021 |
|  | Deaths | ASMR per 100k population | Deaths | ASMR per 100k population | EAPC | Deaths | ASMR per 100k population | Deaths | ASMR per 100k population | EAPC |
|  | NO. (95%UI) | NO. (95%UI) | NO. (95%UI) | NO. (95%UI) | NO. (95%UI) | NO. (95%CI) | NO. (95%UI) | NO. (95%UI) | NO. (95%UI) | NO. (95%CI) |
| **Global** | | |  |  |  |  |  |  |  |  |
| Both | 3,151  (1,251-5,096) | 0.09  (0.03-0.14) | 7,255  (3,060-11,453) | 0.11  (0.04-0.18) | 0.70%  (0.63-0.76) | 38,385  (16,175-62,103) | 4.91  (2.06-7.96) | 92,013  (39,256-147,267) | 4.98  (2.12-7.98) | -0.05%  (-0.09- -0.01) |
| Female | 1,518  (606-2454) | 0.09  (0.03-0.14) | 3,159  (1,347-4,967) | 0.10  (0.04-0.15) | 0.27%  (0.19-0.34) | 21,068  (8,885-34,134) | 4.78  (2.01-7.76) | 45,133  (19,230-72,025) | 4.42  (1.88-7.05) | -0.41%  (-0.46- -0.36) |
| Male | 1,633  (636-2,664) | 0.09  (0.08-0.14) | 4,096  (1,746-6,563) | 0.12  (0.05-0.20) | 1.07%  (1.01-1.12) | 17,317  (7,165-28,144) | 5.03  (2.07-8.20) | 46,880  (20,092-74,977) | 5.65  (2.42-9.06) | 0.34%  (0.31- 0.38) |
| **socio-demographic index** | | |  |  |  |  |  |  |  |  |
| High SDI | 1,058 (441-1,698) | 0.14 (0.06-0.23) | 1,541 (672-2,384) | 0.16 (0.07-0.25) | 0.44  (0.39-0.49) | 20,794 (8,753-33,711) | 8.49 (3.57-13.77) | 34,989 (14,842-56,010) | 7.11 (3.04-11.35) | -0.72  (-0.77- -0.66) |
| High-middle SDI | 1,040 (415-1,689) | 0.13 (0.05-0.22) | 1,930 (817-3,137) | 0.16 (0.07-0.25) | 0.29  (0.16-0.43) | 12,640 (5,375-20,405) | 6.10 (2.59-9.87) | 31,037 (13,310-50,091) | 7.10 (3.04-11.47) | 0.41  (0.32-0.5) |
| Middle SDI | 752 (275-1,240) | 0.07 (0.02-0.11) | 2,531 (1,075-4,011) | 0.12 (0.05-0.18) | 1.84  (1.76-1.92) | 3,485 (1,295-5,706) | 1.73 (0.64-2.84) | 19,123 (8,143-30,803) | 3.36 (1.43-5.42) | 2.17  (2.15-2.2) |
| Low-middle SDI | 228 (85-369) | 0.03 (0.01-0.05) | 969 (399-1,551) | 0.07 (0.03-0.11) | 2.36  (2.28-2.43) | 1,016 (383-1,645) | 0.84 (0.31-1.36) | 5,422 (2,256-8,636) | 1.83 (0.76-2.92) | 2.77  (2.7-2.85) |
| Low SDI | 68 (24-116) | 0.03 (0.01-0.04) | 277 (110-444) | 0.04 (0.02-0.07) | 1.53  (1.4-1.67) | 375 (134-629) | 0.81 (0.28-1.35) | 1,291 (495-2,039) | 1.30 (0.49-2.11) | 1.5  (1.38-1.62) |
| **Region** |  |  |  |  |  |  |  |  |  |  |
| Andean Latin America | 19 (8-32) | 0.08 (0.03-0.14) | 69 (31-116) | 0.08 (0.03-0.14) | 1.44 (1.3-1.59) | 116 (46-194) | 2.79 (1.11-4.69) | 603 (256-1,025) | 4.82 (2.04-8.19) | 1.85 (1.73-1.97) |
| Australasia | 32 (13-52) | 0.19 (0.08-0.3) | 47 (20-75) | 0.1 (0.04-0.16) | -0.07 (-0.27-0.12) | 529 (217-853) | 10.37 (4.25-16.74) | 1,076 (455-1,739) | 8.57 (3.63-13.81) | -0.83 (-0.91--0.75) |
| Caribbean | 27 (11-43) | 0.11 (0.05-0.18) | 63 (27-105) | 0.14 (0.06-0.24) | 1.32 (1.22-1.42) | 217 (89-348) | 4.02 (1.64-6.45) | 778 (325-1,282) | 6.55 (2.73-10.78) | 1.71 (1.65-1.77) |
| Central Asia | 56 (23-91) | 0.15 (0.06-0.24) | 83 (34-136) | 0.11 (0.04-0.17) | -1.03 (-1.17--0.89) | 432 (178-698) | 4.26 (1.76-6.9) | 686 (293-1,096) | 4.04 (1.73-6.46) | 0.22 (0.09-0.35) |
| Central Europe | 200 (84-326) | 0.2 (0.08-0.32) | 201 (87-327) | 0.18 (0.08-0.28) | -0.44 (-0.59--0.28) | 3428 (1481-5548) | 10.57 (4.56-17.11) | 6740 (2,947-10,855) | 13.24 (5.8-21.3) | 0.62 (0.48-0.75) |
| Central Latin America | 80 (33-129) | 0.08 (0.03-0.13) | 392 (174-622) | 0.18 (0.08-0.29) | 2.71 (2.61-2.81) | 449 (187-727) | 2.76 (1.14-4.47) | 2757 (1,211-4,443) | 5.14 (2.25-8.29) | 2.04 (1.97-2.11) |
| Central Sub-Saharan Africa | 6 (2-11) | 0.02 (0.01-0.04) | 38 (14-68) | 0.18 (0.08-0.29) | 2.59 (2.41-2.76) | 44 (16-78) | 0.99 (0.34-1.75) | 216 (80-385) | 2.05 (0.75-3.71) | 2.39 (2.23-2.55) |
| East Asia | 691 (225-1176) | 0.08 (0.03-0.13) | 1,965 (803-3,342) | 0.07 (0.03-0.11) | 1.95 (1.72-2.18) | 3082 (1054-5226) | 1.81 (0.61-3.07) | 18,406 (7,530-30,739) | 3.88 (1.58-6.47) | 2.47 (2.42-2.53) |
| Eastern Europe | 319 (133-502) | 0.19 (0.08-0.3) | 363 (152-579) | 0.11 (0.05-0.18) | -0.4 (-0.55--0.25) | 5062 (2175-8080) | 8.14 (3.49-12.99) | 8,689 (3,730-13,841) | 10.96 (4.7-17.43) | 0.8 (0.68-0.92) |
| Eastern Sub-Saharan Africa | 32 (11-53) | 0.03 (0.01-0.06) | 129 (47-220) | 0.22 (0.1-0.35) | 1.14 (1.04-1.24) | 177 (61-298) | 1.15 (0.39-1.95) | 644 (240-1,072) | 2 (0.73-3.33) | 1.67 (1.57-1.77) |
| High-income Asia Pacific | 123 (45-204) | 0.08 (0.03-0.13) | 119 (46-190) | 0.13 (0.06-0.22) | -0.5 (-0.6--0.41) | 1302 (477-2134) | 3.05 (1.11-5.01) | 4,121 (1,559-6,758) | 3.55 (1.35-5.78) | 0.38 (0.33-0.42) |
| High-income North America | 428 (183-679) | 0.18 (0.08-0.28) | 722 (331-1,106) | 0.18 (0.08-0.29) | 1.14 (1.05-1.22) | 7656 (3270-12354) | 9.77 (4.18-15.74) | 12,047 (5,330-18,866) | 8.06 (3.58-12.59) | -0.81 (-0.92--0.7) |
| North Africa and Middle East | 225 (90-380) | 0.12 (0.05-0.2) | 774 (327-1,237) | 0.24 (0.11-0.37) | 0.75 (0.6-0.9) | 1116 (462-1817) | 3.38 (1.39-5.51) | 4,863 (2,085-7,769) | 5.5 (2.36-8.83) | 1.8 (1.64-1.96) |
| Oceania | 3 (1-5) | 0.08 (0.03-0.13) | 8 (3-14) | 0.16 (0.07-0.27) | 0.17 (0.12-0.23) | 12 (5-20) | 2.07 (0.83-3.55) | 35 (15-57) | 2.42 (1.02-3.94) | 0.55 (0.45-0.65) |
| South Asia | 118 (41-196) | 0.02 (0.01-0.03) | 524 (200-844) | 0.19 (0.08-0.3) | 2.29 (2.24-2.35) | 398 (125-680) | 0.33 (0.1-0.57) | 2,519 (972-4,082) | 0.8 (0.31-1.3) | 2.9 (2.84-2.95) |
| Southeast Asia | 143 (52-236) | 0.05 (0.02-0.08) | 616 (249-1,008) | 0.14 (0.06-0.23) | 2.28 (2.12-2.45) | 509 (174-842) | 0.96 (0.32-1.59) | 3,441 (1,380-5,708) | 2.47 (0.98-4.1) | 3.17 (3.06-3.27) |
| Southern Latin America | 58 (25-97) | 0.16 (0.07-0.27) | 127 (56-207) | 0.04 (0.01-0.06) | 1.32 (1.18-1.45) | 874 (371-1444) | 8.91 (3.78-14.72) | 2,108 (913-3,483) | 10.79 (4.68-17.83) | 0.87 (0.69-1.06) |
| Southern Sub-Saharan Africa | 34 (14-53) | 0.11 (0.04-0.17) | 104 (42-166) | 0.05 (0.02-0.09) | 1.77 (1.44-2.1) | 163 (67-267) | 3.14 (1.27-5.15) | 704 (296-1,118) | 6.26 (2.62-9.96) | 2.38 (2.1-2.67) |
| Tropical Latin America | 97 (39-158) | 0.1 (0.04-0.16) | 388 (166-611) | 0.05 (0.02-0.09) | 1.93 (1.79-2.06) | 602 (244-980) | 3.32 (1.33-5.4) | 3,242 (1,370-5,203) | 5.83 (2.46-9.37) | 1.86 (1.75-1.97) |
| Western Europe | 436 (178-720) | 0.13 (0.06-0.22) | 412 (178-671) | 0.04 (0.02-0.07) | -0.7 (-0.76--0.63) | 12045 (5038-19530) | 9.18 (3.84-14.9) | 17,614 (7,368-29,042) | 7.71 (3.25-12.68) | -0.65 (-0.71--0.59) |
| Western Sub-Saharan Africa | 23 (9-38) | 0.02 (0.01-0.04) | 110 (41-187) | 0.16 (0.07-0.26) | 1.88 (1.83-1.93) | 170 (65-279) | 1 (0.38-1.63) | 724 (285-1,184) | 2.01 (0.79-3.28) | 2.43 (2.37-2.5) |

EOCRC, early-onset colorectal cancer; LOCRC, late-onset colorectal cancer; BMI, body mass index; ASMR, age-standardized mortality rate; EAPC, estimated annual percentage change.

# Table S2 DALYs, ASDR and EAPC in 204 countries in EOCRC and LOCRC attributable to HBMI in 2021

| Location | **EOCRC** | | | **LOCRC** | | |
| --- | --- | --- | --- | --- | --- | --- |
|  | DALYs | ASDR per 100k population | EAPC | DALYs | ASDR per 100k population | EAPC |
|  | NO. (95%UI) | NO. (95%UI) | NO. (95%UI) | NO. (95%UI) | NO. (95%UI) | NO. (95%UI) |
| China | 95,334  (38,733, 162,578) | 7.38  (3, 12.55) | 2.04  (1.79, 2.3) | 411,982  (169,014, 692,218) | 84.57  (34.63, 142.06) | 2.39  (2.31, 2.47) |
| United States of America | 33332  (15302, 50773) | 12.52  (5.74, 19.08) | 1.23  (1.13, 1.34) | 234964  (105,818, 362,954) | 185.97  (83.85, 286.61) | -0.73  (-0.83, -0.63) |
| Brazil | 18693  (7988, 29424) | 9.33  (3.99, 14.69) | 1.94  (1.81, 2.07) | 73727  (31,392, 117,926) | 132.32  (56.3, 211.74) | 1.85  (1.75, 1.96) |
| India | 18376  (6790, 30360) | 1.58  (0.59, 2.61) | 2.08  (1.99, 2.16) | 50738  (19,363, 83,838) | 18.84  (7.18, 31.21) | 2.99  (2.9, 3.08) |
| Russian Federation | 12378  (5084, 19737) | 9.03  (3.7, 14.39) | 0.09  (-0.1, 0.28) | 135067  (57,648, 213,076) | 251.77  (107.39, 396.78) | 0.82  (0.63, 1) |
| Indonesia | 11363  (4372, 19879) | 4.41  (1.7, 7.73) | 2.79  (2.49, 3.08) | 27372  (10,495, 48,861) | 47.65  (17.98, 85.69) | 3.59  (3.4, 3.77) |
| Mexico | 11209  (5054, 17681) | 10.06  (4.53, 15.87) | 3.09  (2.95, 3.23) | 32,964  (14,620, 52,498) | 116.81  (51.72, 186.17) | 2.61  (2.49, 2.73) |
| Turkey | 7614  (3074, 12831) | 9.9  (3.99, 16.71) | -0.92  (-1.3, -0.54) | 37,359  (16,000, 63,595) | 179.97  (76.94, 306.86) | 0.34  (0.01, 0.67) |
| Egypt | 7212  (3000, 12096) | 9.18  (3.82, 15.39) | 2.3  (2.16, 2.44) | 20,445  (8,864, 33,926) | 145.78  (63.26, 242.06) | 3.62  (3.33, 3.91) |
| Philippines | 6107  (2407, 10079) | 6.96  (2.75, 11.47) | 1.92  (1.82, 2.02) | 13,689  (5,250, 22,626) | 72.2  (27.57, 119.46) | 2.97  (2.8, 3.13) |
| Thailand | 6055  (2267, 10908) | 9.64  (3.59, 17.41) | 3.06  (2.67, 3.46) | 23,168  (8,753, 41,634) | 92.96  (35.09, 167.13) | 2.75  (2.59, 2.9) |
| Pakistan | 5089  (1923, 8920) | 3.1  (1.17, 5.43) | 2.76  (2.56, 2.97) | 10,947  (4,301, 18,723) | 40.45  (15.88, 69.14) | 3.14  (2.87, 3.4) |
| Iran (Islamic Republic of) | 4984  (2108, 7878) | 5.83  (2.46, 9.21) | 1.87  (1.68, 2.07) | 15,111  (6,496, 24,009) | 90.33  (38.75, 143.72) | 2.54  (2.32, 2.76) |
| Argentina | 4462  (1978, 7307) | 11.34  (5.02, 18.59) | 1.33  (1.11, 1.56) | 30,287  (13,222, 50,256) | 249.54  (109.05, 413.9) | 0.97  (0.75, 1.19) |
| United Kingdom | 4378  (1906, 6996) | 7.83  (3.4, 12.51) | 0.13  (-0.09, 0.35) | 51,600  (22,331, 83,176) | 180.68  (78.45, 290.14) | -1  (-1.06, -0.93) |
| Saudi Arabia | 4065  (1651, 7298) | 8.95  (3.64, 16.1) | 2.74  (2.5, 2.99) | 5,432  (2,329, 8,879) | 128.47  (54.54, 210.72) | 2.91  (2.5, 3.33) |
| South Africa | 4056  (1667, 6541) | 8.33  (3.43, 13.43) | 1.21  (0.85, 1.57) | 14,016  (5,935, 22,126) | 139.68  (59, 220.92) | 2.26  (1.98, 2.53) |
| Japan | 4039  (1580, 6439) | 3.54  (1.38, 5.68) | -0.3  (-0.42, -0.17) | 59,930  (22,840, 98,389) | 81.78  (31.4, 133.77) | 0.23  (0.16, 0.3) |
| Germany | 3840  (1636, 6399) | 5.71  (2.43, 9.52) | -1.09  (-1.29, -0.89) | 66,836  (27,331, 112,147) | 159.45  (65.48, 266.22) | -1.72  (-1.83, -1.6) |
| Ukraine | 3817  (1555, 6871) | 9.1  (3.71, 16.39) | -1.37  (-1.67, -1.08) | 37,713  (16,115, 64,856) | 219.18  (93.46, 377.02) | -0.12  (-0.25, 0) |
| Colombia | 3433  (1402, 5928) | 8.39  (3.43, 14.49) | 2.68  (2.49, 2.87) | 14,354  (6,020, 24,103) | 117.69  (49.38, 197.67) | 1.61  (1.46, 1.77) |
| Poland | 2771  (1135, 4583) | 7.42  (3.03, 12.27) | -0.26  (-0.46, -0.05) | 45223  (19338, 73500) | 283.66  (121.24, 461.42) | 0.72  (0.53, 0.9) |
| Canada | 2769  (1202, 4575) | 8.97  (3.88, 14.83) | 0.76  (0.53, 0.99) | 25696  (11282, 41849) | 161.47  (70.97, 262.34) | -0.63  (-0.77, -0.5) |
| France | 2730  (1173, 4703) | 5.11  (2.19, 8.78) | 0.38  (0.22, 0.54) | 44400  (18450, 74736) | 143.45  (59.86, 241.71) | -0.24  (-0.34, -0.15) |
| Taiwan (Province of China) | 2541  (1010, 4200) | 10.87  (4.34, 17.95) | 2.42  (2.27, 2.58) | 15686  (6350, 26346) | 165.97  (67.21, 278.96) | 2.23  (1.91, 2.55) |
| Spain | 2492  (1083, 4100) | 5.48  (2.37, 9.04) | -1.47  (-1.7, -1.25) | 43335  (18308, 71688) | 205.64  (87.02, 338.61) | 0.51  (0.32, 0.7) |
| Nigeria | 2373  (868, 4303) | 1.85  (0.68, 3.35) | 2.12  (2.03, 2.2) | 8618  (3368, 14550) | 45.63  (17.89, 76.86) | 2.47  (2.39, 2.54) |
| Italy | 2355  (971, 3874) | 4.29  (1.77, 7.05) | -0.93  (-1.09, -0.78) | 44581  (18687, 73369) | 139.93  (58.86, 229.41) | -0.34  (-0.52, -0.16) |
| Malaysia | 2301  (915, 3954) | 8.54  (3.4, 14.66) | 1.59  (1.33, 1.86) | 8316  (3362, 14245) | 131.44  (52.86, 225) | 1.38  (1.25, 1.52) |
| Venezuela (Bolivarian Republic of) | 2285  (927, 3970) | 9.79  (3.96, 17) | 2.01  (1.86, 2.16) | 9070  (3772, 15495) | 134.89  (56.07, 230.59) | 1.45  (1.34, 1.55) |
| Australia | 1999  (870, 3183) | 9.11  (3.96, 14.51) | 0.21  (0.03, 0.39) | 17251  (7371, 27586) | 175.74  (75.14, 280.51) | -1.03  (-1.13, -0.93) |
| Sudan | 1907  (654, 3770) | 6.71  (2.33, 13.25) | 1.76  (1.68, 1.85) | 3491  (1346, 6515) | 82.3  (31.67, 152.39) | 1.92  (1.83, 2.02) |
| Morocco | 1879  (687, 3645) | 5.96  (2.18, 11.56) | 2.08  (1.93, 2.24) | 7133  (2627, 13035) | 91.92  (33.78, 167.49) | 2.65  (2.54, 2.76) |
| Romania | 1788  (746, 3038) | 9.86  (4.11, 16.72) | 0.65  (0.42, 0.89) | 21829  (8965, 36926) | 272.32  (112.21, 459.66) | 2.2  (1.96, 2.44) |
| Iraq | 1749  (699, 3218) | 5.46  (2.18, 10.03) | 0.51  (0.38, 0.64) | 4568  (1899, 8057) | 87.81  (36.56, 154.16) | 1.22  (1.02, 1.43) |
| Afghanistan | 1723  (465, 3535) | 9.52  (2.55, 19.55) | 0.79  (0.65, 0.92) | 2291  (677, 4599) | 109.75  (33.56, 218.85) | 1.03  (0.95, 1.12) |
| Peru | 1704  (697, 3064) | 5.63  (2.3, 10.12) | 1.09  (0.89, 1.28) | 6759  (2825, 12062) | 93.23  (38.95, 166.3) | 1.26  (1.07, 1.45) |
| Viet Nam | 1681  (557, 3206) | 1.83  (0.61, 3.49) | 4.39  (4.1, 4.68) | 7431  (2457, 13967) | 31.58  (10.3, 59.61) | 4.81  (4.62, 5.01) |
| Myanmar | 1621  (584, 3114) | 3.54  (1.27, 6.79) | 0.27  (0.05, 0.5) | 4972  (1710, 9277) | 43.29  (14.75, 81.13) | 1.27  (1.18, 1.37) |
| Bangladesh | 1544  (537, 2994) | 1.17  (0.41, 2.27) | 3.36  (3.09, 3.64) | 3545  (1152, 6708) | 11.43  (3.69, 21.65) | 2.51  (2.36, 2.67) |
| Republic of Korea | 1509  (524, 2629) | 2.95  (1.02, 5.14) | -0.39  (-0.54, -0.24) | 13039  (4721, 22453) | 61.74  (22.32, 106.37) | 1.13  (0.88, 1.38) |
| Ethiopia | 1441  (486, 2572) | 2.24  (0.76, 4) | -0.86  (-1.09, -0.63) | 4238  (1428, 7386) | 46.29  (15.37, 81.03) | -0.44  (-0.67, -0.21) |
| Chile | 1410  (623, 2300) | 8.66  (3.83, 14.15) | 1.88  (1.68, 2.09) | 9909  (4331, 16175) | 173.69  (75.9, 283.47) | 1.36  (1.24, 1.48) |
| Uzbekistan | 1251  (488, 2180) | 4.38  (1.71, 7.62) | -0.3  (-0.68, 0.09) | 3774  (1498, 6496) | 61.07  (24.13, 105.12) | -0.16  (-0.58, 0.27) |
| United Republic of Tanzania | 1209  (433, 2284) | 3.46  (1.24, 6.5) | 1.91  (1.84, 1.99) | 3581  (1361, 6473) | 65.42  (24.8, 118.39) | 2.03  (1.98, 2.08) |
| Hungary | 1189  (518, 1948) | 12.37  (5.34, 20.28) | -1  (-1.31, -0.68) | 15686  (6980, 25551) | 377.55  (168.71, 614.92) | 0.31  (0.09, 0.54) |
| Ecuador | 1118  (462, 1878) | 7.83  (3.24, 13.14) | 2.78  (2.33, 3.23) | 4012  (1667, 6736) | 112.99  (46.86, 189.86) | 2.85  (2.55, 3.15) |
| Kazakhstan | 1063  (440, 1747) | 6.6  (2.73, 10.85) | -0.99  (-1.2, -0.78) | 5075  (2190, 8213) | 125.03  (53.94, 202.37) | -0.35  (-0.57, -0.12) |
| Algeria | 1038  (427, 1841) | 2.73  (1.12, 4.84) | 0.95  (0.83, 1.06) | 3653  (1501, 6367) | 49.2  (20.15, 85.93) | 1.71  (1.58, 1.84) |
| Kenya | 990  (377, 1722) | 3.07  (1.17, 5.32) | 2.96  (2.74, 3.19) | 2382  (931, 4053) | 46.51  (18.05, 79.29) | 3.72  (3.51, 3.92) |
| Libya | 970  (390, 1722) | 13.31  (5.35, 23.62) | 2.33  (2.19, 2.47) | 2475  (1011, 4386) | 219.27  (89.39, 388.29) | 2.17  (2.01, 2.34) |
| United Arab Emirates | 966  (370, 1765) | 6.44  (2.47, 11.75) | -0.49  (-0.75, -0.23) | 1236  (493, 2178) | 192.51  (78.96, 334.83) | 1.87  (1.4, 2.34) |
| Democratic Republic of the Congo | 935  (305, 1859) | 1.82  (0.6, 3.61) | 2.95  (2.63, 3.28) | 3411  (1194, 6379) | 42.55  (14.75, 80.23) | 2.56  (2.33, 2.78) |
| Cuba | 904  (374, 1575) | 9.1  (3.75, 15.91) | 1.2  (0.99, 1.4) | 7725  (3143, 13340) | 176.09  (71.65, 303.78) | 2.11  (2, 2.22) |
| Czechia | 861  (366, 1460) | 7.91  (3.33, 13.43) | -1.95  (-2.12, -1.78) | 12522  (5388, 20636) | 266.64  (114.62, 439.8) | -1.79  (-1.99, -1.6) |
| Guatemala | 855  (370, 1425) | 7.59  (3.29, 12.65) | 2.26  (1.99, 2.52) | 1778  (755, 2978) | 75.44  (32.03, 126.36) | 2.42  (2.13, 2.71) |
| Netherlands | 850  (349, 1447) | 6.12  (2.53, 10.42) | -0.02  (-0.17, 0.12) | 15628  (6435, 26170) | 201.27  (82.95, 336.68) | 0.4  (0.2, 0.6) |
| Bulgaria | 809  (333, 1380) | 12.43  (5.09, 21.25) | 0.35  (0.15, 0.55) | 9987  (4226, 16961) | 321.59  (136.19, 544.81) | 1.54  (1.37, 1.71) |
| Yemen | 802  (265, 1579) | 3.81  (1.25, 7.5) | 1.79  (1.67, 1.9) | 1708  (601, 3136) | 56.94  (20.11, 104.34) | 2.27  (2.15, 2.4) |
| Syrian Arab Republic | 795  (323, 1428) | 6.76  (2.73, 12.12) | 0.43  (0.13, 0.74) | 3026  (1212, 5295) | 101.19  (40.43, 176.05) | 1.42  (1.24, 1.6) |
| Jordan | 779  (324, 1397) | 7.81  (3.25, 13.99) | -0.37  (-0.56, -0.18) | 2349  (993, 4019) | 144.05  (60.77, 246.98) | 0.22  (-0.05, 0.48) |
| Portugal | 762  (313, 1280) | 7.59  (3.12, 12.73) | 0.01  (-0.31, 0.33) | 9370  (3958, 15585) | 178.68  (76.19, 296.68) | 0.35  (0.07, 0.62) |
| Uganda | 726  (251, 1342) | 3.31  (1.15, 6.11) | 1.84  (1.49, 2.2) | 1578  (554, 2893) | 48.08  (16.73, 88.2) | 1.45  (1.2, 1.69) |
| Cameroon | 721  (259, 1358) | 3.86  (1.39, 7.28) | 1.44  (1.33, 1.55) | 2056  (752, 3828) | 78.86  (28.81, 146.06) | 1.95  (1.86, 2.04) |
| Zimbabwe | 721  (271, 1357) | 7.29  (2.74, 13.66) | 4.91  (3.89, 5.94) | 1764  (704, 3119) | 117.94  (46.73, 208.52) | 3.95  (3.37, 4.55) |
| Belarus | 704  (277, 1231) | 8.09  (3.18, 14.13) | -0.59  (-0.87, -0.3) | 8770  (3664, 15156) | 242.29  (101.18, 419.19) | 0.48  (0.17, 0.8) |
| Serbia | 680  (292, 1182) | 8.22  (3.52, 14.3) | -0.52  (-0.8, -0.24) | 10373  (4582, 17090) | 285.08  (125.88, 469.24) | 0.69  (0.56, 0.83) |
| Zambia | 666  (202, 1713) | 5.75  (1.76, 14.71) | 3  (2.87, 3.12) | 1334  (446, 2932) | 87.36  (29.15, 187.97) | 3.24  (3.13, 3.34) |
| Bolivia (Plurinational State of) | 640  (253, 1222) | 6.92  (2.73, 13.2) | 0.62  (0.47, 0.76) | 2713  (1030, 4972) | 136.4  (51.67, 250.37) | 1.43  (1.4, 1.46) |
| Ghana | 614  (229, 1168) | 2.66  (1, 5.04) | 2.13  (2.06, 2.2) | 1689  (614, 3101) | 48.17  (17.34, 88.37) | 3.29  (3.23, 3.35) |
| Dominican Republic | 606  (246, 1069) | 6.75  (2.75, 11.9) | 2.94  (2.72, 3.15) | 1795  (703, 3208) | 82.41  (32.2, 147.42) | 2.74  (2.58, 2.9) |
| Democratic People's Republic of Korea | 599  (161, 1295) | 2.46  (0.66, 5.33) | 1.02  (0.84, 1.21) | 3209  (1040, 6473) | 44.46  (14.52, 89.78) | 2.14  (2.05, 2.24) |
| Tunisia | 539  (212, 996) | 4.92  (1.93, 9.1) | 1.6  (1.52, 1.67) | 2624  (1047, 4789) | 88.7  (35.36, 161.63) | 1.57  (1.51, 1.63) |
| Costa Rica | 528  (216, 877) | 12.96  (5.31, 21.53) | 3.56  (3.25, 3.88) | 2184  (912, 3604) | 178.92  (74.66, 295.35) | 2.9  (2.7, 3.1) |
| Slovakia | 499  (208, 859) | 9.05  (3.76, 15.63) | -0.89  (-1.03, -0.75) | 7289  (3171, 12221) | 344.13  (149.73, 578.3) | 0.01  (-0.09, 0.11) |
| Azerbaijan | 494  (198, 853) | 5.31  (2.13, 9.17) | -1.2  (-1.53, -0.87) | 2294  (986, 3901) | 93.04  (39.72, 158.63) | 0.32  (0.1, 0.53) |
| Haiti | 489  (171, 954) | 5.03  (1.77, 9.81) | 1.96  (1.84, 2.09) | 989  (336, 1884) | 61.98  (21.02, 118.14) | 2.05  (1.94, 2.15) |
| Angola | 473  (174, 932) | 2.66  (0.98, 5.22) | 2.62  (2.44, 2.8) | 1234  (440, 2356) | 45.84  (16.16, 87.74) | 2.39  (2.27, 2.52) |
| Greece | 468  (201, 755) | 4.9  (2.1, 7.91) | 0.6  (0.38, 0.81) | 8157  (3349, 13483) | 159.12  (65.81, 262.14) | 0.6  (0.43, 0.77) |
| El Salvador | 467  (193, 790) | 9.61  (3.97, 16.23) | 2.69  (2.48, 2.9) | 1406  (584, 2413) | 106.14  (44.22, 182.17) | 2.61  (2.42, 2.8) |
| Kuwait | 464  (214, 762) | 7.77  (3.57, 12.76) | 2.05  (1.43, 2.67) | 878  (405, 1452) | 146.71  (67.25, 243.16) | 2.9  (2.39, 3.41) |
| Belgium | 443  (184, 748) | 4.65  (1.93, 7.86) | -0.99  (-1.17, -0.81) | 6947  (2836, 11747) | 135.19  (55.39, 228.21) | -0.71  (-0.85, -0.58) |
| Cambodia | 410  (137, 796) | 3.2  (1.08, 6.21) | 1.76  (1.68, 1.83) | 1233  (399, 2316) | 42.15  (13.41, 79.46) | 1.78  (1.69, 1.88) |
| New Zealand | 396  (165, 643) | 9.43  (3.94, 15.35) | -0.52  (-0.79, -0.26) | 3920  (1673, 6428) | 209.37  (89.57, 342.44) | -1.32  (-1.4, -1.24) |
| Nepal | 395  (144, 728) | 1.76  (0.64, 3.24) | 2.63  (2.15, 3.11) | 806  (279, 1517) | 15.01  (5.17, 28.34) | 2.7  (2.3, 3.1) |
| Palestine | 389  (159, 656) | 11.31  (4.64, 19.03) | -0.11  (-0.19, -0.03) | 1217  (499, 2009) | 224.72  (91.95, 371.19) | 0.57  (0.42, 0.71) |
| Uruguay | 382  (161, 633) | 13.45  (5.65, 22.32) | 1.09  (1, 1.18) | 3717  (1597, 6178) | 310.89  (134.05, 514.8) | 0.74  (0.62, 0.85) |
| Sweden | 376  (152, 622) | 4.47  (1.8, 7.38) | -0.13  (-0.27, 0.02) | 6212  (2576, 10402) | 130.62  (54.31, 218.44) | -0.3  (-0.47, -0.13) |
| Puerto Rico | 371  (165, 618) | 13.88  (6.18, 23.15) | 0.84  (0.65, 1.02) | 2860  (1276, 4673) | 194.09  (87.1, 316.25) | 0.92  (0.66, 1.18) |
| Somalia | 360  (116, 729) | 3.13  (1.01, 6.35) | 1.11  (1.06, 1.17) | 557  (170, 1119) | 41.6  (12.6, 83.57) | 1.49  (1.43, 1.55) |
| Paraguay | 347  (140, 631) | 6.21  (2.51, 11.28) | 2.66  (2.43, 2.89) | 1537  (630, 2748) | 121.4  (49.71, 217.16) | 3.18  (3, 3.36) |
| Israel | 340  (144, 561) | 4.43  (1.88, 7.32) | -0.72  (-1.01, -0.44) | 3508  (1503, 5913) | 129.55  (55.59, 218.08) | -1.7  (-2.02, -1.37) |
| Croatia | 333  (143, 557) | 8.92  (3.81, 14.91) | 0.07  (-0.19, 0.33) | 6100  (2619, 9888) | 309.02  (132.71, 500.07) | 1.04  (0.82, 1.26) |
| Sri Lanka | 324  (112, 636) | 1.72  (0.6, 3.38) | 1.23  (1.04, 1.41) | 1568  (588, 3033) | 25.41  (9.49, 49.09) | 2.09  (1.88, 2.3) |
| Republic of Moldova | 308  (131, 499) | 8.46  (3.58, 13.71) | 0.05  (-0.22, 0.31) | 3704  (1583, 5959) | 277.15  (118.42, 446.27) | 1.55  (1.13, 1.98) |
| Coted'Ivoire | 307  (111, 597) | 1.73  (0.63, 3.36) | 1.4  (1.28, 1.53) | 716  (265, 1348) | 29.78  (11.07, 55.58) | 1.35  (1.25, 1.45) |
| Kyrgyzstan | 299  (122, 494) | 5.84  (2.39, 9.63) | -0.91  (-1.09, -0.74) | 956  (399, 1560) | 87.62  (36.47, 143.85) | -0.66  (-0.79, -0.52) |
| Lebanon | 297  (117, 504) | 6.11  (2.41, 10.34) | 0.13  (-0.07, 0.34) | 1948  (805, 3257) | 149.68  (62.11, 249.97) | 0.83  (0.62, 1.05) |
| Tajikistan | 287  (108, 555) | 3.93  (1.48, 7.55) | -1.83  (-2.25, -1.4) | 686  (280, 1206) | 51.31  (20.75, 89.77) | -0.98  (-1.18, -0.79) |
| Austria | 279  (113, 478) | 3.54  (1.43, 6.07) | -1.81  (-2, -1.62) | 4671  (1917, 7971) | 117.41  (48.31, 199.83) | -1.89  (-1.95, -1.82) |
| Madagascar | 276  (90, 555) | 1.59  (0.52, 3.22) | 1.55  (1.33, 1.77) | 832  (286, 1566) | 33.81  (11.65, 63.75) | 1.89  (1.73, 2.06) |
| Nicaragua | 272  (110, 465) | 5.2  (2.11, 8.87) | 1.71  (1.49, 1.92) | 811  (331, 1384) | 76.64  (31.27, 131.07) | 2.38  (2.12, 2.65) |
| Panama | 248  (105, 419) | 7.27  (3.09, 12.29) | 3.97  (3.74, 4.19) | 1472  (646, 2452) | 152.68  (67.01, 254.31) | 2.19  (2.1, 2.28) |
| Singapore | 242  (97, 397) | 3.85  (1.55, 6.34) | -0.68  (-1.02, -0.33) | 1671  (664, 2780) | 86.96  (34.39, 145.02) | 0.18  (-0.03, 0.39) |
| Ireland | 234  (96, 394) | 5.08  (2.09, 8.56) | -1.14  (-1.35, -0.93) | 2693  (1129, 4529) | 155.58  (65.22, 261.7) | -0.87  (-0.96, -0.77) |
| Lao People's Democratic Republic | 231  (74, 450) | 4.09  (1.3, 7.93) | 1.92  (1.74, 2.1) | 551  (182, 1074) | 51.76  (17.03, 100.92) | 2.09  (1.98, 2.2) |
| Georgia | 230  (97, 374) | 7.68  (3.24, 12.51) | 0.27  (0.03, 0.51) | 1988  (847, 3283) | 152.16  (64.7, 251.19) | 2.5  (2.05, 2.95) |
| Qatar | 229  (95, 411) | 5.73  (2.38, 10.25) | 0.37  (0.05, 0.69) | 293  (126, 508) | 178.72  (77.37, 308.29) | 0.73  (0.09, 1.36) |
| Honduras | 225  (80, 437) | 3.16  (1.13, 6.1) | 0.56  (0.26, 0.86) | 976  (382, 1737) | 70.65  (27.58, 125.78) | 2.67  (2.49, 2.85) |
| Jamaica | 222  (92, 386) | 9.47  (3.95, 16.51) | 3.06  (2.53, 3.58) | 1197  (511, 2018) | 176.68  (75.35, 297.51) | 2.69  (2.34, 3.05) |
| Norway | 218  (90, 349) | 4.62  (1.91, 7.41) | -1.35  (-1.51, -1.19) | 3379  (1429, 5508) | 151.99  (64.23, 247.46) | -0.66  (-0.76, -0.57) |
| Senegal | 205  (72, 385) | 2.22  (0.78, 4.16) | 1.32  (1.15, 1.48) | 707  (252, 1288) | 42.6  (15.26, 77.47) | 1.97  (1.8, 2.13) |
| Congo | 204  (72, 381) | 5  (1.77, 9.34) | 1.66  (1.46, 1.86) | 454  (168, 821) | 73.09  (26.87, 132.84) | 1.53  (1.42, 1.64) |
| Mali | 204  (69, 391) | 1.76  (0.6, 3.36) | 1.52  (1.32, 1.73) | 573  (193, 1075) | 29.48  (9.82, 55.43) | 0.96  (0.83, 1.09) |
| Denmark | 201  (82, 342) | 4.12  (1.69, 7.02) | -1.6  (-1.87, -1.32) | 4455  (1831, 7575) | 169.59  (69.6, 288.17) | -0.46  (-0.73, -0.19) |
| Bosnia and Herzegovina | 194  (82, 331) | 6.39  (2.69, 10.95) | 0.68  (0.41, 0.95) | 3069  (1286, 5202) | 219.65  (92.06, 372.16) | 2.09  (1.85, 2.34) |
| Switzerland | 192  (80, 320) | 2.42  (1, 4.02) | -1.72  (-1.88, -1.56) | 3635  (1462, 6114) | 90.66  (36.66, 152.26) | -0.87  (-1.02, -0.71) |
| Turkmenistan | 181  (70, 308) | 4.39  (1.7, 7.47) | -0.2  (-0.73, 0.33) | 546  (218, 925) | 58.65  (23.43, 99.65) | -0.29  (-0.78, 0.2) |
| Rwanda | 180  (58, 354) | 2.06  (0.67, 4.06) | -0.93  (-1.43, -0.42) | 495  (153, 973) | 35.84  (11, 70.64) | 0.08  (-0.27, 0.43) |
| Papua New Guinea | 177  (67, 336) | 2.34  (0.89, 4.45) | 0.7  (0.62, 0.78) | 300  (112, 541) | 24.02  (8.85, 43.47) | 0.41  (0.36, 0.47) |
| Malawi | 166  (56, 331) | 1.49  (0.51, 2.97) | 1.77  (1.59, 1.95) | 349  (117, 669) | 22.21  (7.43, 42.32) | 2.3  (2.12, 2.49) |
| Mongolia | 166  (65, 285) | 5.77  (2.26, 9.91) | 0.5  (0.3, 0.71) | 408  (161, 703) | 78.66  (30.87, 135.69) | 0.36  (0.22, 0.49) |
| Lithuania | 164  (68, 277) | 7.21  (2.98, 12.17) | -0.15  (-0.4, 0.11) | 2750  (1180, 4561) | 217.31  (93.29, 360.17) | 0.75  (0.55, 0.94) |
| Finland | 155  (64, 263) | 3.55  (1.46, 6.02) | -0.97  (-1.13, -0.81) | 3384  (1437, 5704) | 122.32  (52.24, 206.03) | -0.1  (-0.18, -0.03) |
| Trinidad and Tobago | 149  (63, 251) | 11.87  (5.05, 20.06) | 1.6  (1.4, 1.8) | 759  (318, 1276) | 173.15  (72.64, 291.32) | 1.02  (0.89, 1.15) |
| Benin | 142  (52, 269) | 1.95  (0.73, 3.68) | 1.19  (1.1, 1.27) | 404  (148, 749) | 36.7  (13.32, 67.85) | 1.57  (1.48, 1.66) |
| Armenia | 140  (57, 230) | 5.43  (2.22, 8.95) | -1.52  (-1.79, -1.25) | 1556  (666, 2498) | 159.49  (68.18, 256.46) | 0.47  (0.3, 0.64) |
| Burkina Faso | 138  (44, 261) | 1.14  (0.36, 2.16) | 1.37  (1.25, 1.5) | 356  (108, 685) | 17.68  (5.3, 34.03) | 2.06  (1.89, 2.22) |
| North Macedonia | 135  (57, 233) | 6.22  (2.6, 10.74) | -0.36  (-0.69, -0.04) | 1692  (727, 2913) | 228.4  (98.03, 392.55) | 1.14  (0.84, 1.44) |
| Bahrain | 117  (47, 207) | 6.55  (2.65, 11.6) | 0.66  (0.42, 0.91) | 267  (109, 467) | 145.97  (59.6, 253.01) | 0.21  (-0.04, 0.46) |
| Latvia | 116  (48, 196) | 7.08  (2.92, 11.93) | -1.16  (-1.36, -0.95) | 1881  (808, 3151) | 218.79  (94.07, 366.6) | 0.26  (0.09, 0.44) |
| Mauritius | 115  (48, 189) | 10.13  (4.22, 16.61) | 3.37  (3.07, 3.67) | 518  (214, 846) | 121.12  (50.1, 198.09) | 2.73  (2.54, 2.92) |
| Togo | 115  (39, 226) | 2.08  (0.7, 4.07) | 2.31  (2.18, 2.45) | 322  (114, 605) | 40.05  (14.03, 75.49) | 2.64  (2.57, 2.7) |
| Mozambique | 113  (40, 221) | 0.71  (0.26, 1.39) | 3.9  (3.69, 4.12) | 409  (137, 750) | 17.33  (5.67, 32.05) | 3.24  (3.05, 3.42) |
| Niger | 107  (35, 221) | 1.03  (0.34, 2.11) | 0.68  (0.55, 0.81) | 369  (123, 728) | 20.47  (6.7, 40.42) | 1.19  (1.12, 1.26) |
| Liberia | 106  (36, 216) | 2.77  (0.94, 5.65) | 1.93  (1.69, 2.17) | 226  (80, 440) | 51.64  (18.19, 100.17) | 1.82  (1.6, 2.04) |
| Slovenia | 105  (45, 182) | 5.52  (2.36, 9.55) | -1.88  (-2.08, -1.69) | 1983  (849, 3268) | 201.08  (86.37, 331.08) | -0.74  (-1.06, -0.42) |
| Gabon | 104  (39, 198) | 8.42  (3.16, 15.94) | 1.49  (1.35, 1.63) | 338  (132, 612) | 143.37  (56.04, 259.59) | 1.55  (1.45, 1.64) |
| Guinea | 100  (33, 193) | 1.39  (0.45, 2.68) | 1.64  (1.59, 1.68) | 303  (103, 568) | 24.86  (8.45, 46.48) | 1.68  (1.64, 1.71) |
| Central African Republic | 96  (31, 195) | 2.86  (0.94, 5.81) | 2.23  (2.17, 2.3) | 237  (79, 470) | 44.65  (14.79, 88.22) | 2.11  (2.06, 2.15) |
| South Sudan | 96  (28, 189) | 1.68  (0.49, 3.3) | 1.77  (1.4, 2.13) | 258  (79, 502) | 29.29  (8.77, 57.09) | 1.81  (1.59, 2.03) |
| Chad | 94  (32, 176) | 1.16  (0.4, 2.17) | 2.17  (2.08, 2.26) | 350  (126, 645) | 28.28  (10.08, 52.36) | 2.34  (2.25, 2.44) |
| Oman | 94  (36, 170) | 1.92  (0.75, 3.45) | 1.1  (0.71, 1.48) | 219  (90, 380) | 53.69  (21.64, 92.62) | 2.21  (1.93, 2.49) |
| Eswatini | 91  (31, 176) | 11.73  (4.05, 22.69) | 3.52  (2.69, 4.35) | 243  (92, 449) | 209.36  (79.72, 385.02) | 2.76  (2.19, 3.33) |
| Botswana | 88  (31, 177) | 4.28  (1.49, 8.6) | 2.31  (1.94, 2.67) | 270  (100, 506) | 90.44  (33.51, 167.59) | 2.45  (2.17, 2.73) |
| Lesotho | 88  (32, 176) | 7.21  (2.58, 14.41) | 5.99  (5.28, 6.7) | 331  (122, 633) | 142.12  (51.91, 272.01) | 4.5  (3.97, 5.04) |
| Burundi | 80  (22, 162) | 1.07  (0.3, 2.18) | -0.1  (-0.43, 0.22) | 236  (74, 464) | 22.24  (6.89, 43.65) | 0.14  (-0.09, 0.37) |
| Eritrea | 79  (24, 159) | 1.79  (0.56, 3.6) | 2.43  (2.37, 2.49) | 210  (68, 405) | 33.66  (10.83, 65.38) | 2.43  (2.38, 2.48) |
| Sierra Leone | 78  (27, 152) | 1.5  (0.53, 2.91) | 3.01  (2.83, 3.19) | 234  (80, 441) | 29.38  (9.99, 55.39) | 2.38  (2.25, 2.5) |
| Mauritania | 75  (28, 140) | 3.02  (1.12, 5.62) | 1.22  (1.11, 1.32) | 314  (118, 555) | 69.78  (26.21, 123.27) | 1.31  (1.16, 1.47) |
| Estonia | 72  (30, 122) | 6.14  (2.59, 10.46) | -1.91  (-2.16, -1.65) | 1279  (543, 2131) | 215.8  (91.56, 357.89) | -0.06  (-0.22, 0.11) |
| Albania | 62  (25, 110) | 2.97  (1.2, 5.28) | 1.08  (0.85, 1.31) | 812  (339, 1417) | 81.92  (34.22, 142.96) | 0.72  (0.51, 0.93) |
| Namibia | 62  (23, 118) | 3.58  (1.32, 6.82) | 1.66  (1.21, 2.11) | 184  (70, 323) | 62.04  (23.58, 108.38) | 2.42  (2.15, 2.69) |
| Bahamas | 61  (24, 102) | 17.33  (6.93, 29) | 1.75  (1.64, 1.87) | 209  (86, 345) | 226.74  (93, 375.56) | 1.76  (1.62, 1.89) |
| Fiji | 60  (24, 108) | 8.11  (3.3, 14.62) | 1.11  (0.78, 1.44) | 234  (96, 404) | 135.64  (55.7, 234.09) | 1.34  (1.16, 1.53) |
| Guyana | 57  (23, 102) | 9.63  (3.9, 17.3) | 2.88  (2.58, 3.17) | 192  (78, 334) | 130.77  (52.9, 227.29) | 1.94  (1.69, 2.19) |
| Brunei Darussalam | 52  (21, 88) | 11.34  (4.53, 19.31) | 1.52  (1.14, 1.9) | 119  (46, 204) | 140.46  (53.89, 240.57) | 1.21  (1.01, 1.41) |
| Equatorial Guinea | 49  (18, 101) | 5.48  (1.97, 11.24) | 2.82  (2.66, 2.97) | 102  (37, 198) | 95.91  (35.1, 185.37) | 3.04  (2.89, 3.19) |
| Solomon Islands | 42  (15, 81) | 8.69  (3.2, 16.7) | 1.54  (1.49, 1.6) | 69  (26, 126) | 87.9  (32.37, 160.22) | 1.18  (0.99, 1.36) |
| Montenegro | 41  (17, 70) | 7.49  (3.16, 12.62) | 0.32  (0.06, 0.58) | 541  (239, 895) | 245.64  (108.41, 407.29) | 1.42  (1.32, 1.52) |
| Cyprus | 40  (17, 73) | 3.02  (1.23, 5.44) | 0.45  (0.1, 0.81) | 522  (210, 923) | 117.33  (47.33, 207.92) | 0.52  (0.37, 0.66) |
| Suriname | 38  (16, 69) | 8.08  (3.26, 14.45) | 1.95  (1.76, 2.14) | 171  (67, 302) | 118.06  (46.01, 208.87) | 1.99  (1.78, 2.21) |
| Guinea-Bissau | 36  (13, 71) | 3.16  (1.1, 6.11) | 1.86  (1.77, 1.94) | 76  (28, 143) | 48.81  (17.9, 90.99) | 2.06  (2, 2.12) |
| Barbados | 32  (14, 53) | 12.5  (5.49, 20.6) | 1.17  (0.91, 1.43) | 323  (139, 543) | 277.46  (119.6, 467.1) | 2.21  (1.95, 2.47) |
| Belize | 26  (12, 41) | 7.69  (3.56, 12.08) | 2.57  (2.09, 3.05) | 79  (36, 125) | 120.76  (54.81, 192.73) | 2.19  (1.71, 2.68) |
| Luxembourg | 24  (10, 39) | 3.78  (1.58, 6.21) | -2.38  (-2.61, -2.16) | 355  (150, 583) | 150.22  (63.82, 247.05) | -1.11  (-1.3, -0.92) |
| Comoros | 23  (8, 43) | 3.98  (1.43, 7.52) | 1.8  (1.47, 2.14) | 67  (24, 126) | 63.53  (22.43, 118.65) | 2.49  (2.38, 2.59) |
| Djibouti | 22  (7, 46) | 2.14  (0.64, 4.46) | 2.3  (2.17, 2.42) | 53  (16, 105) | 37.22  (11.07, 73.52) | 2.44  (2.4, 2.49) |
| Guam | 20  (9, 33) | 16.34  (7.12, 26.42) | 1.99  (1.76, 2.21) | 66  (28, 109) | 136.75  (57.9, 226.57) | 0.17  (-0.17, 0.51) |
| Gambia | 18  (7, 34) | 1.32  (0.49, 2.5) | 1.54  (1.26, 1.81) | 48  (17, 88) | 23.04  (8.33, 42.42) | 1.45  (1.28, 1.62) |
| Malta | 18  (8, 31) | 4.61  (1.96, 7.82) | 0.59  (0.44, 0.74) | 292  (124, 488) | 136.77  (58.43, 227.86) | 0.11  (-0.02, 0.24) |
| Seychelles | 17  (7, 28) | 16.77  (6.95, 28.38) | 2.06  (1.82, 2.29) | 55  (23, 91) | 205.51  (84.1, 340.21) | 2.2  (1.91, 2.5) |
| Vanuatu | 17  (7, 32) | 7.99  (3.04, 14.57) | 1.08  (0.97, 1.18) | 36  (14, 63) | 89.88  (34.36, 159.23) | 1.05  (0.94, 1.16) |
| Bhutan | 16  (6, 32) | 2.58  (0.97, 4.97) | 0.46  (0.4, 0.53) | 44  (17, 84) | 33.48  (12.66, 63.86) | 0.95  (0.86, 1.04) |
| Iceland | 14  (6, 24) | 4.77  (1.99, 7.99) | -0.76  (-0.97, -0.54) | 160  (67, 269) | 123.75  (52.2, 208.47) | -0.62  (-0.75, -0.49) |
| Samoa | 14  (6, 25) | 10.17  (4.12, 17.76) | 1.06  (0.91, 1.21) | 48  (21, 82) | 146.65  (63.34, 250.79) | 0.66  (0.61, 0.72) |
| Saint Lucia | 13  (5, 21) | 7.42  (3.25, 12.37) | 1.22  (1.04, 1.4) | 54  (22, 92) | 100.29  (40.99, 169.32) | 0.68  (0.47, 0.89) |
| Timor-Leste | 13  (4, 26) | 1.58  (0.47, 3.05) | 2.74  (2.24, 3.24) | 34  (10, 67) | 17.98  (5.02, 35.38) | 2.79  (2.51, 3.06) |
| Micronesia (Federated States of) | 12  (5, 21) | 15.2  (5.88, 27.65) | 0.81  (0.78, 0.85) | 31  (12, 55) | 174.38  (68.2, 309.41) | 0.74  (0.7, 0.79) |
| Cabo Verde | 11  (5, 20) | 2.54  (1.01, 4.51) | 2.3  (1.87, 2.73) | 58  (22, 103) | 60.8  (22.85, 107.94) | 4.21  (3.83, 4.6) |
| Kiribati | 10  (4, 18) | 11.49  (4.56, 20.28) | 0.82  (0.71, 0.92) | 19  (8, 33) | 112.54  (44.83, 194.41) | 0.87  (0.8, 0.95) |
| Maldives | 10  (4, 20) | 1.8  (0.68, 3.36) | -0.88  (-0.95, -0.8) | 19  (7, 34) | 25.14  (9.57, 45.52) | -0.42  (-0.56, -0.29) |
| United States Virgin Islands | 10  (4, 18) | 16.22  (6.67, 28.91) | 1.12  (0.96, 1.29) | 67  (28, 114) | 163.28  (68.83, 279.3) | -0.93  (-1.16, -0.7) |
| American Samoa | 8  (4, 14) | 21.11  (9.53, 35.81) | 1.17  (1.07, 1.27) | 26  (12, 44) | 236.28  (107.16, 391.48) | 0.77  (0.67, 0.88) |
| Grenada | 8  (3, 13) | 9.16  (3.84, 15.87) | 1.65  (1.5, 1.79) | 44  (18, 74) | 169.31  (69.61, 286.52) | 2.29  (2.18, 2.4) |
| Saint Vincent and the Grenadines | 8  (3, 13) | 7.99  (3.24, 13.28) | 2.37  (2.21, 2.54) | 34  (14, 57) | 105.95  (43.33, 178.72) | 1.86  (1.69, 2.03) |
| Marshall Islands | 7  (3, 12) | 15.15  (5.95, 27.28) | 1.04  (0.89, 1.19) | 13  (5, 23) | 163.85  (67.87, 284.68) | 0.84  (0.78, 0.9) |
| Northern Mariana Islands | 7  (3, 12) | 15.54  (6.78, 26.22) | 0.35  (0.09, 0.61) | 29  (13, 48) | 232.9  (99.62, 380.58) | 0.92  (0.7, 1.15) |
| Bermuda | 6  (3, 11) | 11.11  (4.77, 18.54) | 0.32  (0.12, 0.53) | 75  (32, 123) | 244.49  (106.98, 402.02) | -0.22  (-0.38, -0.07) |
| Greenland | 6  (2, 11) | 14.4  (5.46, 25.56) | -1.54  (-1.62, -1.47) | 49  (20, 85) | 290.03  (119.95, 504.3) | -1.38  (-1.46, -1.29) |
| Andorra | 5  (2, 10) | 5.17  (1.88, 10.08) | -0.61  (-0.85, -0.38) | 52  (20, 97) | 151.91  (57.69, 281.64) | -0.52  (-0.77, -0.28) |
| Antigua and Barbuda | 5  (2, 8) | 5.93  (2.55, 9.61) | 1.57  (1.33, 1.82) | 39  (17, 63) | 164.44  (69.81, 266.29) | 1.96  (1.78, 2.14) |
| Dominica | 5  (2, 8) | 8.43  (3.55, 14.75) | 2.3  (2.14, 2.47) | 39  (17, 66) | 208.47  (89.02, 347.22) | 1.55  (1.48, 1.62) |
| Sao Tome and Principe | 5  (2, 9) | 3.09  (1.14, 5.99) | 1.79  (1.57, 2.02) | 18  (7, 31) | 75.86  (28.49, 133.28) | 2.66  (2.6, 2.72) |
| Tonga | 5  (2, 9) | 7.54  (3.19, 13.41) | 0.46  (0.37, 0.54) | 20  (9, 34) | 115.85  (51.65, 193.87) | 0.76  (0.66, 0.87) |
| Monaco | 4  (1, 7) | 12.81  (4.97, 24.54) | 1.07  (0.93, 1.2) | 61  (25, 107) | 282.11  (118.24, 497.21) | 0.74  (0.63, 0.85) |
| Saint Kitts and Nevis | 4  (2, 7) | 7.08  (3.02, 12.16) | -0.75  (-1.21, -0.3) | 31  (13, 50) | 193.94  (81.66, 319.23) | 1.96  (1.79, 2.13) |
| Nauru | 2  (1, 4) | 26.52  (9.86, 48.11) | 0.38  (0.33, 0.42) | 4  (1, 6) | 271.96  (104.34, 482.72) | 0.48  (0.43, 0.53) |
| Palau | 2  (1, 3) | 10.9  (4.53, 19.03) | 0.18  (0.05, 0.31) | 9  (4, 16) | 180.07  (73.23, 320.56) | 0.3  (0.23, 0.37) |
| Cook Islands | 1  (0, 2) | 6.47  (2.79, 11.5) | -0.24  (-0.54, 0.07) | 5  (2, 8) | 84.29  (37.53, 142.59) | -0.56  (-0.72, -0.39) |
| San Marino | 1  (0, 2) | 4.14  (1.39, 7.98) | -0.45  (-0.8, -0.09) | 20  (7, 38) | 122.91  (44.85, 231.37) | -0.75  (-1.08, -0.43) |
| Tuvalu | 1  (0, 2) | 13.03  (5.37, 22.93) | 1.01  (0.96, 1.05) | 3  (1, 6) | 143.67  (59.05, 247.21) | 0.91  (0.88, 0.93) |
| Niue | 0  (0, 0) | 12.89  (5.6, 22.66) | 0.66  (0.55, 0.76) | 1  (0, 1) | 163.21  (69.97, 277.71) | 1  (0.96, 1.04) |
| Tokelau | 0  (0, 0) | 12.07  (5.11, 21.17) | 0.8  (0.68, 0.92) | 0  (0, 1) | 134.18  (57.04, 232.1) | 0.67  (0.66, 0.68) |

DALYs, disability-adjusted life years; ASDR, age-standardized disability-adjusted life years rate; EAPC, estimated annual percentage change; EOCRC, early-onset colorectal cancer; LOCRC, late-onset colorectal cancer; HBMI, high body-mass index; UI, uncertainty interval

# Table S3 Deaths, ASMR and EAPC in 204 countries in EOCRC and LOCRC attributable to HBMI in 2021

| location | **EOCRC** | | | **LOCRC** | | |
| --- | --- | --- | --- | --- | --- | --- |
|  | Deaths | ASMR per 100k population | EAPC | Deaths | ASMR per 100k population | EAPC |
|  | NO. (95%UI) | NO. (95%UI) | NO. (95%UI) | NO. (95%UI) | NO. (95%UI) | NO. (95%UI) |
| China | 1902  (774, 3,245) | 0.14  (0.06, 0.25) | 1.95  (1.71, 2.2) | 17,516  (7,158, 29,364) | 3.81  (1.55, 6.39) | 2.46  (2.38, 2.53) |
| United States of America | 668  (307, 1,018) | 0.25  (0.11, 0.38) | 1.18  (1.08, 1.28) | 10,734  (4,769, 16,815) | 8.1  (3.61, 12.64) | -0.85  (-0.97, -0.73) |
| Brazil | 381  (163, 600) | 0.19  (0.08, 0.3) | 1.92  (1.78, 2.05) | 3,174  (1,341, 5,091) | 5.84  (2.46, 9.37) | 1.83  (1.72, 1.94) |
| India | 381  (142, 628) | 0.03  (0.01, 0.05) | 2.06  (1.98, 2.14) | 1,937  (737, 3,215) | 0.76  (0.29, 1.27) | 3.07  (2.99, 3.15) |
| Russian Federation | 255  (105, 407) | 0.18  (0.08, 0.29) | 0.02  (-0.17, 0.2) | 6,231  (2,647, 9,899) | 11.61  (4.93, 18.41) | 1.12  (0.95, 1.29) |
| Indonesia | 233  (90, 406) | 0.09  (0.03, 0.16) | 2.79  (2.49, 3.08) | 1,003  (377, 1,806) | 1.97  (0.72, 3.58) | 3.71  (3.55, 3.87) |
| Mexico | 226  (102, 356) | 0.2  (0.09, 0.32) | 3.07  (2.93, 3.21) | 1,356  (597, 2,164) | 5.02  (2.2, 8.01) | 2.3  (2.17, 2.44) |
| Turkey | 153  (62, 257) | 0.2  (0.08, 0.33) | -0.99  (-1.37, -0.61) | 1,642  (698, 2,809) | 8.3  (3.52, 14.25) | 0.55  (0.19, 0.91) |
| Egypt | 143  (59, 239) | 0.18  (0.08, 0.31) | 2.23  (2.1, 2.36) | 784  (340, 1,304) | 6.57  (2.85, 10.93) | 3.88  (3.55, 4.2) |
| Philippines | 123  (48, 202) | 0.14  (0.06, 0.23) | 1.95  (1.85, 2.05) | 518  (197, 857) | 2.97  (1.12, 4.93) | 2.98  (2.83, 3.14) |
| Thailand | 123  (46, 222) | 0.19  (0.07, 0.34) | 2.99  (2.62, 3.36) | 958  (358, 1,733) | 3.91  (1.46, 7.07) | 2.79  (2.64, 2.94) |
| Pakistan | 103  (39, 180) | 0.06  (0.02, 0.11) | 2.74  (2.53, 2.94) | 418  (164, 714) | 1.71  (0.67, 2.93) | 3.17  (2.92, 3.43) |
| Iran (Islamic Republic of) | 98  (42, 155) | 0.11  (0.05, 0.18) | 1.79  (1.59, 1.99) | 644  (276, 1,029) | 4.14  (1.77, 6.63) | 2.71  (2.49, 2.92) |
| Argentina | 91  (40, 148) | 0.23  (0.1, 0.38) | 1.26  (1.03, 1.48) | 1,442  (623, 2,408) | 11.56  (5, 19.3) | 0.94  (0.71, 1.17) |
| United Kingdom | 88  (38, 140) | 0.16  (0.07, 0.25) | -0.01  (-0.24, 0.21) | 2,795  (1,205, 4,546) | 8.96  (3.88, 14.52) | -0.79  (-0.87, -0.72) |
| Japan | 82  (32, 131) | 0.07  (0.03, 0.11) | -0.4  (-0.53, -0.27) | 3,417  (1,286, 5,641) | 3.73  (1.42, 6.11) | 0.36  (0.29, 0.42) |
| Saudi Arabia | 82  (33, 147) | 0.18  (0.07, 0.33) | 2.69  (2.44, 2.95) | 189  (80, 310) | 5.6  (2.34, 9.23) | 2.85  (2.44, 3.27) |
| South Africa | 82  (34, 133) | 0.17  (0.07, 0.27) | 1.3  (0.96, 1.65) | 594  (250, 942) | 6.47  (2.72, 10.29) | 2.13  (1.85, 2.41) |
| Ukraine | 80  (32, 143) | 0.19  (0.08, 0.34) | -1.39  (-1.69, -1.1) | 1619  (691, 2,764) | 9.22  (3.93, 15.76) | -0.09  (-0.21, 0.04) |
| Germany | 78  (33, 130) | 0.11  (0.05, 0.19) | -1.21  (-1.42, -0.99) | 3,553  (1,434, 6,016) | 7.6  (3.08, 12.79) | -1.77  (-1.89, -1.65) |
| Colombia | 68  (28, 118) | 0.17  (0.07, 0.29) | 2.6  (2.41, 2.79) | 637  (268, 1,076) | 5.26  (2.21, 8.87) | 1.54  (1.39, 1.68) |
| Poland | 58  (24, 96) | 0.15  (0.06, 0.26) | -0.3  (-0.51, -0.08) | 2,288  (973, 3,748) | 13.81  (5.87, 22.62) | 0.82  (0.63, 1) |
| Canada | 55  (24, 90) | 0.18  (0.08, 0.29) | 0.64  (0.41, 0.87) | 1,311  (571, 2,149) | 7.69  (3.36, 12.59) | -0.5  (-0.63, -0.36) |
| France | 54  (23, 94) | 0.1  (0.04, 0.17) | 0.2  (0.03, 0.37) | 2,520  (1,038, 4,233) | 7.03  (2.91, 11.8) | -0.3  (-0.39, -0.2) |
| Spain | 51  (22, 83) | 0.11  (0.05, 0.18) | -1.52  (-1.76, -1.28) | 2,321  (972, 3,881) | 9.72  (4.08, 16.15) | 0.45  (0.27, 0.64) |
| Taiwan (Province of China) | 51  (20, 85) | 0.22  (0.09, 0.36) | 2.42  (2.28, 2.57) | 739  (299, 1,233) | 7.74  (3.13, 12.91) | 2.44  (2.08, 2.8) |
| Italy | 48  (20, 79) | 0.09  (0.04, 0.14) | -1.04  (-1.2, -0.89) | 2,495  (1,035, 4,130) | 6.85  (2.85, 11.29) | -0.18  (-0.33, -0.03) |
| Nigeria | 48  (18, 87) | 0.04  (0.01, 0.07) | 2.1  (2.01, 2.19) | 367  (144, 617) | 2.22  (0.88, 3.74) | 2.62  (2.54, 2.7) |
| Malaysia | 47  (19, 80) | 0.17  (0.07, 0.3) | 1.59  (1.32, 1.86) | 339  (136, 578) | 5.75  (2.28, 9.81) | 1.66  (1.54, 1.79) |
| Venezuela (Bolivarian Republic of) | 46  (19, 80) | 0.19  (0.08, 0.34) | 1.9  (1.75, 2.05) | 384  (160, 658) | 5.98  (2.48, 10.25) | 1.46  (1.36, 1.56) |
| Australia | 39  (17, 63) | 0.18  (0.08, 0.28) | 0.05  (-0.14, 0.24) | 877  (371, 1,413) | 8.28  (3.51, 13.3) | -0.8  (-0.9, -0.7) |
| Morocco | 39  (14, 75) | 0.12  (0.04, 0.24) | 2.09  (1.95, 2.24) | 287  (105, 522) | 3.99  (1.46, 7.23) | 2.76  (2.65, 2.87) |
| Romania | 38  (16, 64) | 0.2  (0.08, 0.34) | 0.62  (0.38, 0.87) | 1,011  (411, 1,721) | 11.95  (4.87, 20.3) | 2.33  (2.1, 2.56) |
| Sudan | 38  (13, 74) | 0.14  (0.05, 0.27) | 1.69  (1.6, 1.77) | 137  (53, 252) | 3.56  (1.36, 6.52) | 2.05  (1.95, 2.15) |
| Iraq | 36  (14, 65) | 0.11  (0.04, 0.2) | 0.46  (0.34, 0.59) | 178  (74, 311) | 3.9  (1.62, 6.78) | 1.48  (1.24, 1.71) |
| Afghanistan | 35  (10, 72) | 0.2  (0.05, 0.4) | 0.73  (0.6, 0.86) | 81  (25, 161) | 4.38  (1.4, 8.61) | 1.19  (1.09, 1.3) |
| Viet Nam | 35  (12, 67) | 0.04  (0.01, 0.07) | 4.43  (4.14, 4.72) | 276  (89, 524) | 1.26  (0.4, 2.4) | 4.67  (4.51, 4.83) |
| Peru | 34  (14, 61) | 0.11  (0.05, 0.2) | 1.03  (0.83, 1.22) | 306  (127, 545) | 4.26  (1.77, 7.58) | 1.4  (1.21, 1.58) |
| Myanmar | 33  (12, 63) | 0.07  (0.03, 0.14) | 0.27  (0.05, 0.5) | 185  (63, 348) | 1.72  (0.57, 3.26) | 1.45  (1.37, 1.53) |
| Bangladesh | 31  (11, 60) | 0.02  (0.01, 0.05) | 3.34  (3.06, 3.61) | 133  (42, 254) | 0.45  (0.14, 0.86) | 2.42  (2.29, 2.55) |
| Republic of Korea | 30  (11, 53) | 0.06  (0.02, 0.1) | -0.47  (-0.63, -0.32) | 626  (222, 1,086) | 3.02  (1.07, 5.25) | 1.35  (1.16, 1.53) |
| Chile | 29  (13, 47) | 0.17  (0.08, 0.29) | 1.84  (1.63, 2.06) | 472  (204, 778) | 8.22  (3.54, 13.53) | 1.35  (1.24, 1.47) |
| Ethiopia | 29  (10, 51) | 0.05  (0.02, 0.08) | -0.93  (-1.16, -0.7) | 171  (56, 300) | 2.04  (0.65, 3.6) | -0.08  (-0.31, 0.15) |
| Hungary | 25  (11, 41) | 0.25  (0.11, 0.41) | -1.02  (-1.36, -0.68) | 729  (322, 1,189) | 16.48  (7.32, 26.89) | 0.12  (-0.11, 0.35) |
| Uzbekistan | 25  (10, 44) | 0.09  (0.03, 0.15) | -0.34  (-0.73, 0.05) | 140  (55, 240) | 2.49  (0.98, 4.29) | -0.05  (-0.46, 0.36) |
| United Republic of Tanzania | 24  (9, 46) | 0.07  (0.03, 0.13) | 1.86  (1.79, 1.93) | 145  (55, 262) | 2.95  (1.11, 5.37) | 2.28  (2.23, 2.33) |
| Ecuador | 22  (9, 37) | 0.16  (0.06, 0.26) | 2.74  (2.31, 3.18) | 180  (74, 306) | 5.23  (2.13, 8.89) | 2.93  (2.61, 3.25) |
| Kazakhstan | 22  (9, 36) | 0.14  (0.06, 0.22) | -1.03  (-1.24, -0.82) | 205  (88, 331) | 5.47  (2.36, 8.85) | -0.13  (-0.39, 0.14) |
| Algeria | 21  (9, 37) | 0.05  (0.02, 0.1) | 0.9  (0.79, 1.02) | 162  (66, 284) | 2.51  (1.02, 4.4) | 2.01  (1.83, 2.18) |
| Kenya | 20  (8, 35) | 0.06  (0.02, 0.11) | 2.95  (2.73, 3.18) | 90  (35, 153) | 1.95  (0.75, 3.35) | 3.96  (3.74, 4.19) |
| Libya | 20  (8, 35) | 0.27  (0.11, 0.48) | 2.3  (2.15, 2.44) | 99  (40, 176) | 9.86  (3.99, 17.42) | 2.33  (2.16, 2.51) |
| Cuba | 19  (8, 32) | 0.18  (0.08, 0.32) | 1.18  (0.98, 1.39) | 357  (144, 618) | 7.99  (3.21, 13.81) | 2.2  (2.11, 2.29) |
| Democratic Republic of the Congo | 19  (6, 38) | 0.04  (0.01, 0.08) | 2.95  (2.63, 3.27) | 130  (45, 244) | 1.87  (0.64, 3.58) | 2.64  (2.41, 2.86) |
| United Arab Emirates | 19  (7, 35) | 0.12  (0.05, 0.23) | -0.68  (-0.99, -0.37) | 43  (17, 76) | 9.88  (4.03, 17.22) | 2.85  (2.29, 3.41) |
| Czechia | 18  (8, 30) | 0.16  (0.07, 0.27) | -2.09  (-2.26, -1.91) | 623  (267, 1,029) | 12.54  (5.38, 20.71) | -1.69  (-1.88, -1.49) |
| Bulgaria | 17  (7, 29) | 0.26  (0.1, 0.44) | 0.31  (0.11, 0.51) | 472  (198, 804) | 14.49  (6.1, 24.65) | 1.65  (1.45, 1.85) |
| Guatemala | 17  (7, 28) | 0.15  (0.07, 0.26) | 2.26  (2, 2.53) | 78  (33, 131) | 3.49  (1.47, 5.84) | 2.07  (1.76, 2.39) |
| Netherlands | 17  (7, 28) | 0.12  (0.05, 0.2) | -0.13  (-0.27, 0.02) | 766  (313, 1,286) | 9.31  (3.81, 15.61) | 0.32  (0.14, 0.5) |
| Jordan | 16  (7, 28) | 0.16  (0.07, 0.28) | -0.47  (-0.66, -0.28) | 93  (39, 159) | 6.62  (2.78, 11.41) | 0.56  (0.32, 0.8) |
| Portugal | 16  (6, 26) | 0.15  (0.06, 0.25) | -0.03  (-0.36, 0.29) | 507  (211, 848) | 8.49  (3.56, 14.15) | 0.24  (0.01, 0.48) |
| Syrian Arab Republic | 16  (7, 29) | 0.13  (0.05, 0.24) | 0.37  (0.07, 0.67) | 122  (48, 211) | 4.7  (1.86, 8.1) | 1.67  (1.5, 1.83) |
| Yemen | 16  (5, 32) | 0.08  (0.03, 0.16) | 1.72  (1.61, 1.83) | 67  (24, 123) | 2.48  (0.88, 4.54) | 2.48  (2.35, 2.62) |
| Belarus | 15  (6, 25) | 0.17  (0.07, 0.29) | -0.66  (-0.95, -0.37) | 381  (158, 656) | 10.5  (4.35, 18.15) | 0.61  (0.29, 0.92) |
| Cameroon | 15  (5, 27) | 0.08  (0.03, 0.15) | 1.4  (1.29, 1.51) | 84  (31, 155) | 3.72  (1.36, 6.85) | 2.16  (2.07, 2.24) |
| Uganda | 15  (5, 27) | 0.07  (0.02, 0.12) | 1.81  (1.45, 2.16) | 59  (21, 108) | 2  (0.68, 3.67) | 1.54  (1.32, 1.77) |
| Zimbabwe | 15  (6, 28) | 0.15  (0.06, 0.28) | 4.87  (3.85, 5.91) | 68  (27, 120) | 5.09  (1.99, 9) | 3.86  (3.33, 4.39) |
| Serbia | 14  (6, 25) | 0.17  (0.07, 0.3) | -0.55  (-0.85, -0.26) | 486  (214, 809) | 12.89  (5.67, 21.45) | 0.59  (0.48, 0.69) |
| Bolivia (Plurinational State of) | 13  (5, 25) | 0.14  (0.06, 0.27) | 0.6  (0.46, 0.75) | 116  (44, 214) | 6.26  (2.36, 11.56) | 1.68  (1.64, 1.72) |
| Zambia | 13  (4, 34) | 0.12  (0.04, 0.3) | 3  (2.87, 3.13) | 49  (16, 105) | 3.61  (1.2, 7.56) | 3.33  (3.23, 3.42) |
| Democratic People's Republic of Korea | 12  (3, 26) | 0.05  (0.01, 0.11) | 1  (0.81, 1.18) | 151  (50, 305) | 2.2  (0.74, 4.48) | 2.32  (2.22, 2.42) |
| Dominican Republic | 12  (5, 21) | 0.14  (0.06, 0.24) | 2.9  (2.69, 3.11) | 77  (29, 139) | 3.59  (1.37, 6.52) | 2.75  (2.55, 2.96) |
| Ghana | 12  (5, 24) | 0.05  (0.02, 0.1) | 2.11  (2.04, 2.18) | 69  (25, 126) | 2.27  (0.8, 4.17) | 3.6  (3.54, 3.66) |
| Tunisia | 11  (4, 20) | 0.1  (0.04, 0.18) | 1.52  (1.45, 1.6) | 113  (45, 205) | 4.06  (1.61, 7.34) | 1.63  (1.56, 1.7) |
| Angola | 10  (4, 19) | 0.05  (0.02, 0.11) | 2.57  (2.39, 2.74) | 45  (16, 85) | 1.92  (0.67, 3.68) | 2.55  (2.41, 2.68) |
| Azerbaijan | 10  (4, 17) | 0.11  (0.04, 0.19) | -1.16  (-1.48, -0.84) | 86  (37, 146) | 3.88  (1.65, 6.64) | 0.6  (0.36, 0.84) |
| Costa Rica | 10  (4, 17) | 0.26  (0.11, 0.43) | 3.48  (3.17, 3.79) | 96  (39, 158) | 7.89  (3.26, 13.1) | 2.82  (2.63, 3.01) |
| Greece | 10  (4, 16) | 0.1  (0.04, 0.16) | 0.59  (0.39, 0.79) | 458  (186, 763) | 7.67  (3.14, 12.74) | 0.45  (0.23, 0.67) |
| Haiti | 10  (3, 19) | 0.1  (0.04, 0.2) | 1.95  (1.83, 2.08) | 38  (13, 72) | 2.64  (0.89, 5.05) | 2.09  (1.99, 2.19) |
| Slovakia | 10  (4, 18) | 0.19  (0.08, 0.32) | -0.97  (-1.12, -0.82) | 332  (143, 556) | 15.51  (6.69, 26.04) | 0.15  (0.06, 0.25) |
| Belgium | 9  (4, 15) | 0.09  (0.04, 0.16) | -1.1  (-1.28, -0.92) | 379  (153, 643) | 6.61  (2.68, 11.2) | -0.75  (-0.86, -0.65) |
| El Salvador | 9  (4, 16) | 0.19  (0.08, 0.32) | 2.64  (2.44, 2.85) | 63  (26, 108) | 4.6  (1.9, 7.9) | 2.62  (2.42, 2.81) |
| Kuwait | 9  (4, 15) | 0.15  (0.07, 0.25) | 1.93  (1.32, 2.54) | 36  (16, 60) | 7.07  (3.2, 11.74) | 3.18  (2.7, 3.66) |
| Cambodia | 8  (3, 16) | 0.07  (0.02, 0.13) | 1.76  (1.69, 1.84) | 46  (14, 86) | 1.7  (0.52, 3.22) | 1.95  (1.84, 2.06) |
| Nepal | 8  (3, 15) | 0.04  (0.01, 0.07) | 2.63  (2.14, 3.13) | 30  (10, 56) | 0.58  (0.19, 1.1) | 2.82  (2.44, 3.21) |
| New Zealand | 8  (3, 13) | 0.19  (0.08, 0.3) | -0.63  (-0.9, -0.36) | 199  (83, 329) | 10.18  (4.26, 16.78) | -0.91  (-0.98, -0.83) |
| Palestine | 8  (3, 13) | 0.23  (0.09, 0.38) | -0.2  (-0.27, -0.12) | 50  (20, 82) | 10.63  (4.34, 17.58) | 0.7  (0.53, 0.87) |
| Sweden | 8  (3, 13) | 0.09  (0.04, 0.15) | -0.22  (-0.37, -0.08) | 351  (145, 588) | 6.6  (2.73, 11.03) | -0.17  (-0.34, -0.01) |
| Uruguay | 8  (3, 13) | 0.27  (0.11, 0.45) | 0.98  (0.89, 1.07) | 194  (83, 325) | 14.87  (6.36, 24.83) | 0.8  (0.68, 0.91) |
| Croatia | 7  (3, 12) | 0.18  (0.08, 0.31) | 0.01  (-0.25, 0.28) | 310  (133, 505) | 14.75  (6.33, 24.03) | 1.09  (0.9, 1.29) |
| Israel | 7  (3, 11) | 0.09  (0.04, 0.15) | -0.81  (-1.09, -0.52) | 193  (82, 325) | 6.67  (2.83, 11.24) | -1.63  (-1.96, -1.3) |
| Paraguay | 7  (3, 13) | 0.13  (0.05, 0.23) | 2.65  (2.42, 2.88) | 67  (27, 121) | 5.55  (2.25, 9.96) | 3.27  (3.1, 3.45) |
| Puerto Rico | 7  (3, 12) | 0.27  (0.12, 0.45) | 0.77  (0.59, 0.95) | 136  (60, 224) | 8.23  (3.64, 13.5) | 0.65  (0.39, 0.92) |
| Somalia | 7  (2, 15) | 0.07  (0.02, 0.13) | 1.09  (1.05, 1.14) | 19  (6, 39) | 1.65  (0.49, 3.31) | 1.63  (1.58, 1.69) |
| Sri Lanka | 7  (2, 13) | 0.03  (0.01, 0.07) | 1.25  (1.06, 1.44) | 65  (25, 126) | 1.11  (0.41, 2.14) | 2.14  (1.96, 2.33) |
| Austria | 6  (2, 10) | 0.07  (0.03, 0.12) | -1.94  (-2.14, -1.74) | 250  (101, 429) | 5.71  (2.33, 9.77) | -1.86  (-1.92, -1.8) |
| Coted'Ivoire | 6  (2, 12) | 0.04  (0.01, 0.07) | 1.35  (1.23, 1.48) | 28  (10, 51) | 1.33  (0.5, 2.46) | 1.45  (1.35, 1.55) |
| Kyrgyzstan | 6  (2, 10) | 0.12  (0.05, 0.19) | -0.97  (-1.14, -0.79) | 37  (15, 61) | 3.73  (1.54, 6.18) | -0.46  (-0.63, -0.29) |
| Lebanon | 6  (2, 10) | 0.12  (0.05, 0.21) | 0.06  (-0.13, 0.26) | 100  (41, 169) | 7.32  (2.99, 12.3) | 1.05  (0.83, 1.27) |
| Madagascar | 6  (2, 11) | 0.03  (0.01, 0.07) | 1.53  (1.31, 1.76) | 31  (11, 58) | 1.51  (0.52, 2.85) | 2.04  (1.88, 2.2) |
| Republic of Moldova | 6  (3, 10) | 0.18  (0.07, 0.29) | 0.02  (-0.25, 0.29) | 155  (66, 251) | 11.61  (4.93, 18.78) | 1.5  (1.05, 1.95) |
| Tajikistan | 6  (2, 11) | 0.08  (0.03, 0.15) | -1.85  (-2.26, -1.44) | 26  (10, 45) | 2.17  (0.87, 3.79) | -0.84  (-1.06, -0.62) |
| Georgia | 5  (2, 8) | 0.16  (0.07, 0.25) | 0.33  (0.08, 0.57) | 86  (37, 143) | 6.47  (2.75, 10.66) | 2.77  (2.27, 3.27) |
| Honduras | 5  (2, 9) | 0.07  (0.02, 0.13) | 0.64  (0.36, 0.93) | 40  (16, 71) | 3.11  (1.21, 5.55) | 2.77  (2.58, 2.97) |
| Ireland | 5  (2, 8) | 0.1  (0.04, 0.17) | -1.3  (-1.51, -1.09) | 134  (56, 227) | 7.45  (3.09, 12.59) | -0.72  (-0.82, -0.63) |
| Lao People's Democratic Republic | 5  (1, 9) | 0.08  (0.03, 0.16) | 1.89  (1.72, 2.07) | 20  (7, 40) | 2.13  (0.69, 4.15) | 2.16  (2.08, 2.24) |
| Nicaragua | 5  (2, 9) | 0.1  (0.04, 0.18) | 1.67  (1.46, 1.89) | 34  (14, 58) | 3.38  (1.37, 5.83) | 2.37  (2.08, 2.66) |
| Panama | 5  (2, 8) | 0.14  (0.06, 0.24) | 3.88  (3.64, 4.11) | 69  (30, 115) | 7.1  (3.11, 11.81) | 2.08  (2, 2.17) |
| Singapore | 5  (2, 8) | 0.08  (0.03, 0.13) | -0.8  (-1.14, -0.46) | 74  (28, 125) | 3.94  (1.52, 6.68) | 0.44  (0.24, 0.64) |
| Bosnia and Herzegovina | 4  (2, 7) | 0.13  (0.06, 0.23) | 0.68  (0.4, 0.96) | 141  (59, 239) | 9.95  (4.15, 16.84) | 2.25  (2.03, 2.47) |
| Congo | 4  (1, 8) | 0.1  (0.04, 0.19) | 1.57  (1.37, 1.77) | 16  (6, 30) | 3  (1.09, 5.51) | 1.73  (1.63, 1.83) |
| Denmark | 4  (2, 7) | 0.08  (0.03, 0.14) | -1.69  (-1.97, -1.42) | 238  (96, 406) | 8.43  (3.41, 14.36) | -0.26  (-0.54, 0.03) |
| Jamaica | 4  (2, 8) | 0.19  (0.08, 0.33) | 3.03  (2.52, 3.55) | 53  (22, 90) | 7.75  (3.28, 13.06) | 2.58  (2.26, 2.9) |
| Mali | 4  (1, 8) | 0.04  (0.01, 0.07) | 1.5  (1.29, 1.71) | 22  (7, 42) | 1.27  (0.41, 2.4) | 1.09  (0.95, 1.22) |
| Norway | 4  (2, 7) | 0.09  (0.04, 0.15) | -1.46  (-1.62, -1.3) | 183  (77, 299) | 7.6  (3.19, 12.43) | -0.51  (-0.61, -0.41) |
| Papua New Guinea | 4  (1, 7) | 0.05  (0.02, 0.09) | 0.67  (0.59, 0.75) | 10  (4, 18) | 0.91  (0.33, 1.66) | 0.44  (0.39, 0.5) |
| Qatar | 4  (2, 8) | 0.11  (0.05, 0.2) | 0.28  (-0.05, 0.6) | 11  (5, 18) | 9.07  (3.91, 15.6) | 0.83  (0.15, 1.51) |
| Rwanda | 4  (1, 7) | 0.04  (0.01, 0.08) | -0.96  (-1.47, -0.44) | 19  (6, 38) | 1.59  (0.48, 3.15) | 0.46  (0.15, 0.78) |
| Senegal | 4  (1, 8) | 0.05  (0.02, 0.09) | 1.32  (1.16, 1.49) | 30  (11, 54) | 1.97  (0.71, 3.59) | 2.15  (1.99, 2.31) |
| Switzerland | 4  (2, 6) | 0.05  (0.02, 0.08) | -1.76  (-1.92, -1.59) | 199  (79, 335) | 4.47  (1.78, 7.51) | -0.72  (-0.87, -0.58) |
| Turkmenistan | 4  (1, 6) | 0.09  (0.03, 0.15) | -0.25  (-0.78, 0.29) | 20  (8, 35) | 2.4  (0.96, 4.09) | -0.24  (-0.73, 0.26) |
| Armenia | 3  (1, 5) | 0.11  (0.05, 0.18) | -1.49  (-1.76, -1.22) | 70  (30, 113) | 7.26  (3.08, 11.71) | 0.86  (0.68, 1.05) |
| Benin | 3  (1, 5) | 0.04  (0.02, 0.08) | 1.14  (1.06, 1.23) | 16  (6, 30) | 1.65  (0.59, 3.03) | 1.88  (1.79, 1.98) |
| Burkina Faso | 3  (1, 5) | 0.02  (0.01, 0.04) | 1.38  (1.25, 1.5) | 14  (4, 27) | 0.77  (0.22, 1.48) | 2.14  (1.99, 2.28) |
| Finland | 3  (1, 5) | 0.07  (0.03, 0.12) | -1.06  (-1.22, -0.9) | 181  (76, 307) | 5.87  (2.48, 9.93) | -0.09  (-0.19, 0.01) |
| Lithuania | 3  (1, 6) | 0.15  (0.06, 0.25) | -0.21  (-0.46, 0.04) | 141  (60, 234) | 10.26  (4.4, 17.09) | 0.99  (0.81, 1.17) |
| Malawi | 3  (1, 7) | 0.03  (0.01, 0.06) | 1.77  (1.59, 1.95) | 14  (5, 26) | 0.97  (0.32, 1.84) | 2.31  (2.15, 2.47) |
| Mongolia | 3  (1, 6) | 0.12  (0.05, 0.2) | 0.46  (0.25, 0.67) | 15  (6, 27) | 3.36  (1.31, 5.8) | 0.53  (0.4, 0.67) |
| North Macedonia | 3  (1, 5) | 0.13  (0.05, 0.22) | -0.39  (-0.72, -0.06) | 76  (33, 130) | 11.12  (4.77, 19.01) | 1.4  (1.05, 1.74) |
| Trinidad and Tobago | 3  (1, 5) | 0.24  (0.1, 0.4) | 1.53  (1.34, 1.73) | 32  (13, 54) | 7.46  (3.08, 12.56) | 0.88  (0.76, 1.01) |
| Bahrain | 2  (1, 4) | 0.13  (0.05, 0.23) | 0.52  (0.28, 0.76) | 10  (4, 18) | 7.33  (2.98, 12.6) | 0.62  (0.32, 0.91) |
| Botswana | 2  (1, 4) | 0.09  (0.03, 0.18) | 2.26  (1.9, 2.62) | 11  (4, 21) | 4.4  (1.64, 8.06) | 2.66  (2.38, 2.95) |
| Burundi | 2  (0, 3) | 0.02  (0.01, 0.04) | -0.15  (-0.47, 0.18) | 9  (3, 18) | 0.98  (0.3, 1.92) | 0.41  (0.2, 0.62) |
| Central African Republic | 2  (1, 4) | 0.06  (0.02, 0.12) | 2.2  (2.14, 2.27) | 8  (3, 16) | 1.78  (0.58, 3.52) | 2.16  (2.13, 2.2) |
| Chad | 2  (1, 4) | 0.02  (0.01, 0.05) | 2.14  (2.06, 2.23) | 14  (5, 26) | 1.26  (0.44, 2.35) | 2.47  (2.38, 2.57) |
| Eritrea | 2  (1, 3) | 0.04  (0.01, 0.07) | 2.37  (2.32, 2.43) | 8  (2, 15) | 1.42  (0.45, 2.77) | 2.66  (2.61, 2.71) |
| Eswatini | 2  (1, 4) | 0.24  (0.08, 0.47) | 3.5  (2.68, 4.34) | 10  (4, 18) | 9.7  (3.74, 17.72) | 2.67  (2.17, 3.17) |
| Gabon | 2  (1, 4) | 0.17  (0.07, 0.33) | 1.46  (1.33, 1.6) | 13  (5, 24) | 6.29  (2.45, 11.44) | 1.75  (1.66, 1.84) |
| Guinea | 2  (1, 4) | 0.03  (0.01, 0.06) | 1.59  (1.55, 1.64) | 12  (4, 23) | 1.07  (0.36, 1.99) | 1.79  (1.76, 1.83) |
| Latvia | 2  (1, 4) | 0.15  (0.06, 0.25) | -1.19  (-1.39, -0.98) | 95  (41, 160) | 10.13  (4.34, 17.04) | 0.5  (0.31, 0.69) |
| Lesotho | 2  (1, 4) | 0.15  (0.05, 0.3) | 5.98  (5.27, 6.7) | 13  (5, 25) | 6.3  (2.28, 12.16) | 4.37  (3.87, 4.88) |
| Liberia | 2  (1, 4) | 0.06  (0.02, 0.12) | 1.86  (1.63, 2.1) | 9  (3, 17) | 2.28  (0.8, 4.43) | 2.04  (1.82, 2.26) |
| Mauritania | 2  (1, 3) | 0.06  (0.02, 0.12) | 1.2  (1.09, 1.3) | 14  (5, 25) | 3.48  (1.3, 6.13) | 1.61  (1.45, 1.77) |
| Mauritius | 2  (1, 4) | 0.2  (0.09, 0.33) | 3.31  (3.01, 3.61) | 21  (9, 35) | 5.16  (2.11, 8.46) | 2.79  (2.61, 2.97) |
| Mozambique | 2  (1, 5) | 0.01  (0.01, 0.03) | 3.88  (3.67, 4.1) | 16  (5, 30) | 0.78  (0.25, 1.46) | 3.21  (3.03, 3.39) |
| Niger | 2  (1, 5) | 0.02  (0.01, 0.04) | 0.68  (0.54, 0.81) | 14  (5, 28) | 0.91  (0.29, 1.81) | 1.37  (1.28, 1.45) |
| Oman | 2  (1, 3) | 0.04  (0.02, 0.07) | 1.01  (0.62, 1.4) | 8  (3, 15) | 2.43  (0.96, 4.21) | 2.58  (2.33, 2.83) |
| Sierra Leone | 2  (1, 3) | 0.03  (0.01, 0.06) | 2.96  (2.79, 3.14) | 10  (3, 18) | 1.34  (0.45, 2.53) | 2.44  (2.32, 2.57) |
| Slovenia | 2  (1, 4) | 0.11  (0.05, 0.19) | -2.01  (-2.21, -1.81) | 107  (46, 178) | 10.04  (4.27, 16.61) | -0.44  (-0.74, -0.15) |
| South Sudan | 2  (1, 4) | 0.03  (0.01, 0.07) | 1.75  (1.39, 2.12) | 9  (3, 18) | 1.24  (0.36, 2.42) | 1.89  (1.69, 2.08) |
| Togo | 2  (1, 5) | 0.04  (0.01, 0.08) | 2.28  (2.15, 2.41) | 13  (4, 24) | 1.88  (0.65, 3.55) | 2.84  (2.76, 2.91) |
| Albania | 1  (1, 2) | 0.06  (0.02, 0.11) | 1.11  (0.88, 1.33) | 41  (17, 72) | 4.24  (1.77, 7.4) | 0.86  (0.65, 1.07) |
| Bahamas | 1  (0, 2) | 0.34  (0.14, 0.58) | 1.71  (1.6, 1.83) | 9  (3, 14) | 10.1  (4.1, 16.87) | 1.96  (1.81, 2.11) |
| Barbados | 1  (0, 1) | 0.25  (0.11, 0.41) | 1.17  (0.9, 1.43) | 15  (6, 26) | 13.11  (5.54, 22.15) | 2.28  (1.99, 2.56) |
| Belize | 1  (0, 1) | 0.15  (0.07, 0.24) | 2.53  (2.06, 3.01) | 3  (1, 5) | 5.39  (2.41, 8.64) | 2.2  (1.69, 2.71) |
| Brunei Darussalam | 1  (0, 2) | 0.23  (0.09, 0.39) | 1.55  (1.16, 1.94) | 4  (2, 7) | 5.56  (2.08, 9.59) | 1.3  (1.09, 1.52) |
| Cyprus | 1  (0, 1) | 0.06  (0.02, 0.11) | 0.33  (0, 0.67) | 26  (10, 46) | 6.05  (2.42, 10.73) | 0.44  (0.3, 0.59) |
| Equatorial Guinea | 1  (0, 2) | 0.11  (0.04, 0.23) | 2.72  (2.55, 2.88) | 4  (1, 8) | 4.31  (1.57, 8.25) | 3.54  (3.4, 3.69) |
| Estonia | 1  (1, 3) | 0.13  (0.05, 0.21) | -1.97  (-2.23, -1.71) | 67  (29, 113) | 10.35  (4.39, 17.33) | 0.26  (0.11, 0.41) |
| Fiji | 1  (0, 2) | 0.16  (0.07, 0.3) | 1.08  (0.75, 1.41) | 9  (4, 16) | 6.21  (2.54, 10.72) | 1.5  (1.33, 1.66) |
| Guinea-Bissau | 1  (0, 1) | 0.07  (0.02, 0.13) | 1.84  (1.75, 1.93) | 3  (1, 5) | 2.13  (0.77, 3.96) | 2.28  (2.22, 2.34) |
| Guyana | 1  (0, 2) | 0.2  (0.08, 0.35) | 2.85  (2.55, 3.14) | 8  (3, 13) | 5.65  (2.28, 9.84) | 1.96  (1.71, 2.22) |
| Montenegro | 1  (0, 1) | 0.15  (0.06, 0.26) | 0.3  (0.04, 0.56) | 25  (11, 42) | 11.93  (5.26, 19.89) | 1.69  (1.58, 1.81) |
| Namibia | 1  (0, 2) | 0.07  (0.03, 0.14) | 1.66  (1.21, 2.11) | 7  (3, 13) | 2.78  (1.06, 4.86) | 2.55  (2.3, 2.8) |
| Solomon Islands | 1  (0, 2) | 0.18  (0.07, 0.34) | 1.52  (1.46, 1.57) | 2  (1, 4) | 3.52  (1.28, 6.46) | 1.21  (1.05, 1.37) |
| Suriname | 1  (0, 1) | 0.16  (0.07, 0.29) | 1.93  (1.73, 2.12) | 7  (3, 13) | 5.1  (1.94, 9.11) | 2.07  (1.85, 2.28) |
| American Samoa | 0  (0, 0) | 0.42  (0.19, 0.72) | 1.13  (1.03, 1.23) | 1  (0, 2) | 10.37  (4.65, 17.21) | 0.89  (0.78, 0.99) |
| Andorra | 0  (0, 0) | 0.1  (0.04, 0.2) | -0.72  (-0.97, -0.47) | 3  (1, 5) | 7.18  (2.74, 13.26) | -0.48  (-0.75, -0.21) |
| Antigua and Barbuda | 0  (0, 0) | 0.12  (0.05, 0.19) | 1.55  (1.29, 1.81) | 2  (1, 3) | 7.67  (3.2, 12.52) | 2.12  (1.93, 2.31) |
| Bermuda | 0  (0, 0) | 0.22  (0.09, 0.36) | 0.18  (-0.03, 0.39) | 4  (2, 6) | 11.8  (5.05, 19.6) | -0.13  (-0.29, 0.03) |
| Bhutan | 0  (0, 1) | 0.05  (0.02, 0.1) | 0.43  (0.36, 0.5) | 2  (1, 3) | 1.37  (0.51, 2.61) | 1.23  (1.13, 1.32) |
| Cabo Verde | 0  (0, 0) | 0.05  (0.02, 0.09) | 2.31  (1.88, 2.73) | 3  (1, 5) | 3.16  (1.17, 5.64) | 4.62  (4.23, 5.01) |
| Comoros | 0  (0, 1) | 0.08  (0.03, 0.15) | 1.79  (1.47, 2.1) | 3  (1, 5) | 2.87  (1.01, 5.34) | 2.67  (2.57, 2.77) |
| Cook Islands | 0  (0, 0) | 0.13  (0.06, 0.23) | -0.29  (-0.6, 0.01) | 0  (0, 0) | 3.77  (1.65, 6.45) | -0.48  (-0.63, -0.32) |
| Djibouti | 0  (0, 1) | 0.04  (0.01, 0.09) | 2.26  (2.15, 2.38) | 2  (1, 4) | 1.63  (0.48, 3.24) | 2.6  (2.55, 2.64) |
| Dominica | 0  (0, 0) | 0.17  (0.07, 0.3) | 2.23  (2.07, 2.38) | 2  (1, 3) | 9.99  (4.22, 16.69) | 1.59  (1.51, 1.67) |
| Gambia | 0  (0, 1) | 0.03  (0.01, 0.05) | 1.52  (1.24, 1.79) | 2  (1, 4) | 0.99  (0.35, 1.83) | 1.58  (1.44, 1.72) |
| Greenland | 0  (0, 0) | 0.3  (0.11, 0.53) | -1.52  (-1.59, -1.45) | 2  (1, 3) | 13.16  (5.39, 22.87) | -1.39  (-1.48, -1.29) |
| Grenada | 0  (0, 0) | 0.18  (0.08, 0.32) | 1.66  (1.5, 1.81) | 2  (1, 3) | 7.6  (3.05, 12.94) | 2.54  (2.4, 2.69) |
| Guam | 0  (0, 1) | 0.33  (0.14, 0.53) | 2.03  (1.8, 2.26) | 3  (1, 4) | 5.32  (2.22, 8.88) | -0.26  (-0.63, 0.12) |
| Iceland | 0  (0, 0) | 0.09  (0.04, 0.16) | -0.92  (-1.15, -0.7) | 8  (3, 14) | 6.02  (2.5, 10.21) | -0.43  (-0.57, -0.28) |
| Kiribati | 0  (0, 0) | 0.23  (0.09, 0.41) | 0.79  (0.69, 0.89) | 1  (0, 1) | 4.7  (1.86, 8.16) | 1.09  (1.01, 1.17) |
| Luxembourg | 0  (0, 1) | 0.08  (0.03, 0.12) | -2.43  (-2.66, -2.21) | 19  (8, 31) | 7.56  (3.19, 12.43) | -0.99  (-1.18, -0.81) |
| Maldives | 0  (0, 0) | 0.04  (0.01, 0.07) | -1.02  (-1.09, -0.94) | 1  (0, 1) | 1.03  (0.38, 1.87) | -0.04  (-0.18, 0.09) |
| Malta | 0  (0, 1) | 0.09  (0.04, 0.16) | 0.48  (0.32, 0.64) | 15  (6, 26) | 6.56  (2.77, 11.02) | 0.17  (0.04, 0.3) |
| Marshall Islands | 0  (0, 0) | 0.31  (0.12, 0.55) | 1.03  (0.87, 1.18) | 0  (0, 1) | 6.97  (2.86, 12.08) | 0.91  (0.86, 0.97) |
| Micronesia (Federated States of) | 0  (0, 0) | 0.31  (0.12, 0.56) | 0.79  (0.75, 0.83) | 1  (0, 2) | 7.31  (2.83, 13.04) | 0.88  (0.84, 0.91) |
| Monaco | 0  (0, 0) | 0.25  (0.1, 0.48) | 0.97  (0.85, 1.1) | 3  (1, 6) | 13.72  (5.69, 24.44) | 0.82  (0.72, 0.93) |
| Nauru | 0  (0, 0) | 0.53  (0.2, 0.97) | 0.36  (0.32, 0.4) | 0  (0, 0) | 11.02  (4.19, 19.62) | 0.62  (0.58, 0.66) |
| Niue | 0  (0, 0) | 0.26  (0.11, 0.45) | 0.62  (0.53, 0.72) | 0  (0, 0) | 7.24  (3.06, 12.29) | 1.17  (1.13, 1.22) |
| Northern Mariana Islands | 0  (0, 0) | 0.31  (0.14, 0.52) | 0.33  (0.06, 0.6) | 1  (0, 2) | 10.32  (4.38, 16.94) | 0.99  (0.79, 1.19) |
| Palau | 0  (0, 0) | 0.22  (0.09, 0.38) | 0.1  (-0.03, 0.22) | 0  (0, 1) | 8.71  (3.5, 15.63) | 0.63  (0.55, 0.71) |
| Saint Kitts and Nevis | 0  (0, 0) | 0.15  (0.06, 0.25) | -0.71  (-1.14, -0.27) | 1  (1, 2) | 9.04  (3.74, 14.92) | 2.11  (1.92, 2.29) |
| Saint Lucia | 0  (0, 0) | 0.15  (0.07, 0.25) | 1.17  (0.99, 1.36) | 2  (1, 4) | 4.61  (1.85, 7.83) | 0.61  (0.37, 0.85) |
| Saint Vincent and the Grenadines | 0  (0, 0) | 0.16  (0.07, 0.27) | 2.38  (2.21, 2.56) | 1  (1, 3) | 4.85  (1.96, 8.24) | 1.92  (1.75, 2.09) |
| Samoa | 0  (0, 1) | 0.21  (0.08, 0.36) | 1.02  (0.87, 1.17) | 2  (1, 3) | 6.19  (2.62, 10.62) | 0.72  (0.67, 0.77) |
| San Marino | 0  (0, 0) | 0.08  (0.03, 0.16) | -0.51  (-0.86, -0.15) | 1  (0, 2) | 5.94  (2.2, 11.13) | -0.82  (-1.17, -0.48) |
| Sao Tome and Principe | 0  (0, 0) | 0.06  (0.02, 0.12) | 1.77  (1.55, 1.99) | 1  (0, 1) | 3.66  (1.37, 6.45) | 2.87  (2.8, 2.94) |
| Seychelles | 0  (0, 1) | 0.34  (0.14, 0.57) | 2.04  (1.8, 2.27) | 2  (1, 4) | 9.03  (3.65, 15.15) | 2.5  (2.21, 2.79) |
| Timor-Leste | 0  (0, 1) | 0.03  (0.01, 0.06) | 2.75  (2.26, 3.24) | 1  (0, 3) | 0.72  (0.19, 1.43) | 2.66  (2.38, 2.93) |
| Tokelau | 0  (0, 0) | 0.24  (0.1, 0.42) | 0.74  (0.63, 0.84) | 0  (0, 0) | 5.88  (2.47, 10.2) | 0.83  (0.81, 0.85) |
| Tonga | 0  (0, 0) | 0.15  (0.06, 0.27) | 0.4  (0.31, 0.49) | 1  (0, 1) | 5.11  (2.24, 8.58) | 1.04  (0.92, 1.16) |
| Tuvalu | 0  (0, 0) | 0.26  (0.11, 0.46) | 0.98  (0.93, 1.02) | 0  (0, 0) | 6.09  (2.48, 10.53) | 1.1  (1.08, 1.12) |
| United States Virgin Islands | 0  (0, 0) | 0.31  (0.13, 0.55) | 0.99  (0.82, 1.15) | 3  (1, 6) | 7.88  (3.27, 13.52) | -0.9  (-1.14, -0.66) |
| Vanuatu | 0  (0, 1) | 0.16  (0.06, 0.3) | 1.04  (0.94, 1.14) | 1  (0, 2) | 3.75  (1.43, 6.7) | 1.07  (0.98, 1.15) |

ASMR, age-standardized mortality rate; EAPC, estimated annual percentage change; EOCRC, early-onset colorectal cancer; LOCRC, late-onset colorectal cancer; HBMI, high body-mass index; UI, uncertainty interval

# Table S4 the predicted DALYs and ASDR of EOCRC and LOCRC attributable to high BMI from 2021 to 2040 based on Nordpred model

| Year | EOCRC | | | | | | LOCRC | | | | | |
| --- | --- | --- | --- | --- | --- | --- | --- | --- | --- | --- | --- | --- |
|  | DALYs | | | ASDR | | | DALYs | | | ASDR | | |
|  | Both | Male | Female | Both | Male | Female | Both | Male | Female | Both | Male | Female |
| 1992 | 167,864 | 87,972 | 79,892 | 8.23 | 8.46 | 8.00 | 908,348 | 436,227 | 472,122 | 103.00 | 107.49 | 98.90 |
| 1993 | 176,269 | 92,565 | 83,704 | 8.40 | 8.65 | 8.14 | 940,434 | 453,314 | 487,120 | 104.40 | 109.08 | 100.05 |
| 1994 | 185,056 | 97,328 | 87,728 | 8.54 | 8.82 | 8.26 | 965,599 | 467,664 | 497,935 | 105.02 | 110.10 | 100.30 |
| 1995 | 192,257 | 101,146 | 91,111 | 8.62 | 8.91 | 8.32 | 983,375 | 479,407 | 503,967 | 104.91 | 110.64 | 99.61 |
| 1996 | 198,023 | 104,322 | 93,701 | 8.62 | 8.92 | 8.30 | 997,281 | 489,040 | 508,242 | 104.29 | 110.57 | 98.49 |
| 1997 | 202,966 | 107,073 | 95,893 | 8.62 | 8.95 | 8.29 | 1,016,718 | 500,373 | 516,345 | 103.98 | 110.61 | 97.88 |
| 1998 | 207,641 | 109,595 | 98,046 | 8.61 | 8.95 | 8.26 | 1,041,482 | 514,599 | 526,883 | 104.13 | 111.19 | 97.67 |
| 1999 | 214,834 | 113,255 | 101,579 | 8.73 | 9.07 | 8.38 | 1,076,338 | 533,317 | 543,020 | 105.02 | 112.44 | 98.29 |
| 2000 | 221,930 | 118,089 | 103,841 | 8.80 | 9.23 | 8.35 | 1,104,313 | 551,040 | 553,274 | 105.29 | 113.44 | 97.91 |
| 2001 | 227,718 | 121,877 | 105,841 | 8.86 | 9.35 | 8.35 | 1,133,849 | 568,804 | 565,045 | 105.43 | 114.17 | 97.55 |
| 2002 | 233,353 | 125,232 | 108,121 | 8.90 | 9.44 | 8.36 | 1,165,376 | 588,365 | 577,011 | 105.62 | 115.03 | 97.15 |
| 2003 | 238,999 | 128,574 | 110,424 | 8.96 | 9.52 | 8.38 | 1,198,457 | 607,028 | 591,429 | 105.86 | 115.63 | 97.11 |
| 2004 | 243,223 | 131,438 | 111,786 | 8.97 | 9.58 | 8.34 | 1,221,078 | 622,176 | 598,902 | 105.00 | 115.32 | 95.78 |
| 2005 | 247,198 | 133,777 | 113,421 | 8.97 | 9.60 | 8.32 | 1,254,201 | 642,168 | 612,033 | 104.97 | 115.82 | 95.30 |
| 2006 | 249,522 | 135,507 | 114,015 | 8.90 | 9.56 | 8.22 | 1,274,790 | 656,076 | 618,714 | 103.73 | 114.96 | 93.73 |
| 2007 | 255,742 | 139,687 | 116,054 | 8.94 | 9.68 | 8.19 | 1,307,201 | 676,225 | 630,976 | 103.39 | 115.06 | 92.99 |
| 2008 | 265,447 | 145,444 | 120,004 | 9.08 | 9.87 | 8.28 | 1,350,877 | 702,534 | 648,343 | 103.89 | 116.15 | 92.96 |
| 2009 | 274,272 | 151,075 | 123,197 | 9.18 | 10.04 | 8.31 | 1,380,891 | 721,931 | 658,960 | 103.22 | 115.97 | 91.89 |
| 2010 | 282,401 | 156,844 | 125,557 | 9.26 | 10.22 | 8.29 | 1,423,453 | 747,374 | 676,079 | 103.42 | 116.69 | 91.67 |
| 2011 | 288,480 | 160,657 | 127,823 | 9.29 | 10.28 | 8.29 | 1,465,195 | 771,166 | 694,028 | 103.38 | 116.89 | 91.42 |
| 2012 | 295,181 | 164,946 | 130,234 | 9.36 | 10.39 | 8.31 | 1,509,681 | 798,044 | 711,637 | 103.36 | 117.33 | 91.00 |
| 2013 | 302,824 | 169,447 | 133,377 | 9.48 | 10.55 | 8.41 | 1,551,466 | 822,732 | 728,734 | 103.04 | 117.28 | 90.44 |
| 2014 | 309,598 | 173,490 | 136,107 | 9.60 | 10.69 | 8.49 | 1,597,151 | 847,056 | 750,095 | 102.86 | 117.08 | 90.30 |
| 2015 | 317,675 | 178,609 | 139,066 | 9.76 | 10.90 | 8.59 | 1,659,225 | 881,332 | 777,892 | 103.68 | 118.18 | 90.89 |
| 2016 | 327,300 | 184,435 | 142,865 | 9.96 | 11.14 | 8.75 | 1,721,399 | 915,281 | 806,118 | 104.35 | 119.03 | 91.40 |
| 2017 | 335,154 | 189,288 | 145,867 | 10.11 | 11.34 | 8.86 | 1,772,566 | 942,429 | 830,137 | 104.25 | 118.88 | 91.36 |
| 2018 | 343,506 | 194,015 | 149,491 | 10.27 | 11.52 | 9.00 | 1,835,457 | 977,187 | 858,269 | 104.77 | 119.57 | 91.71 |
| 2019 | 351,011 | 198,767 | 152,245 | 10.40 | 11.70 | 9.09 | 1,897,229 | 1,011,341 | 885,889 | 105.12 | 120.07 | 91.93 |
| 2020 | 352,722 | 199,785 | 152,937 | 10.37 | 11.66 | 9.05 | 1,941,936 | 1,036,923 | 905,012 | 104.70 | 119.85 | 91.37 |
| 2021 | 359,539 | 203,646 | 155,893 | 10.49 | 11.80 | 9.16 | 2,005,125 | 1,065,245 | 939,880 | 105.45 | 120.22 | 92.51 |
| 2022 | 380,560 | 216,387 | 164,173 | 10.80 | 12.23 | 9.36 | 2,084,164 | 1,111,558 | 972,606 | 105.80 | 120.91 | 92.46 |
| 2023 | 390,140 | 222,266 | 167,874 | 10.96 | 12.44 | 9.47 | 2,147,583 | 1,145,841 | 1,001,742 | 106.10 | 121.30 | 92.68 |
| 2024 | 400,049 | 228,332 | 171,717 | 11.12 | 12.65 | 9.57 | 2,213,339 | 1,181,295 | 1,032,044 | 106.41 | 121.69 | 92.90 |
| 2025 | 409,174 | 233,996 | 175,178 | 11.24 | 12.82 | 9.66 | 2,279,233 | 1,216,579 | 1,062,654 | 106.68 | 122.02 | 93.09 |
| 2026 | 418,496 | 239,792 | 178,704 | 11.37 | 13.00 | 9.74 | 2,345,518 | 1,252,031 | 1,093,487 | 106.94 | 122.35 | 93.28 |
| 2027 | 427,956 | 245,692 | 182,263 | 11.50 | 13.18 | 9.82 | 2,411,385 | 1,287,212 | 1,124,174 | 107.20 | 122.67 | 93.47 |
| 2028 | 437,822 | 251,844 | 185,977 | 11.63 | 13.35 | 9.90 | 2,477,660 | 1,322,546 | 1,155,114 | 107.46 | 123.00 | 93.66 |
| 2029 | 448,153 | 258,279 | 189,873 | 11.76 | 13.53 | 9.98 | 2,545,303 | 1,358,543 | 1,186,761 | 107.72 | 123.33 | 93.85 |
| 2030 | 456,841 | 263,651 | 193,190 | 11.85 | 13.65 | 10.03 | 2,611,975 | 1,393,750 | 1,218,225 | 107.91 | 123.56 | 93.99 |
| 2031 | 465,155 | 268,848 | 196,307 | 11.93 | 13.77 | 10.08 | 2,679,216 | 1,429,183 | 1,250,033 | 108.10 | 123.79 | 94.13 |
| 2032 | 472,668 | 273,633 | 199,035 | 12.02 | 13.89 | 10.13 | 2,746,692 | 1,464,654 | 1,282,038 | 108.29 | 124.02 | 94.26 |
| 2033 | 479,238 | 277,913 | 201,325 | 12.10 | 14.00 | 10.18 | 2,815,400 | 1,500,701 | 1,314,699 | 108.48 | 124.25 | 94.40 |
| 2034 | 485,114 | 281,824 | 203,290 | 12.19 | 14.12 | 10.23 | 2,885,973 | 1,537,673 | 1,348,301 | 108.66 | 124.49 | 94.54 |
| 2035 | 488,294 | 283,662 | 204,632 | 12.22 | 14.16 | 10.27 | 2,954,968 | 1,573,529 | 1,381,439 | 108.76 | 124.60 | 94.61 |
| 2036 | 491,048 | 285,302 | 205,746 | 12.26 | 14.19 | 10.30 | 3,024,073 | 1,609,374 | 1,414,699 | 108.86 | 124.71 | 94.68 |
| 2037 | 493,545 | 286,856 | 206,689 | 12.29 | 14.22 | 10.33 | 3,092,457 | 1,644,753 | 1,447,704 | 108.95 | 124.82 | 94.75 |
| 2038 | 495,465 | 288,160 | 207,305 | 12.33 | 14.26 | 10.37 | 3,161,394 | 1,680,304 | 1,481,090 | 109.05 | 124.93 | 94.83 |
| 2039 | 496,761 | 289,182 | 207,580 | 12.36 | 14.29 | 10.40 | 3,231,789 | 1,716,516 | 1,515,273 | 109.14 | 125.04 | 94.90 |
| 2040 | 497,919 | 290,168 | 207,751 | 12.40 | 14.32 | 10.43 | 3,302,310 | 1,752,741 | 1,549,569 | 109.24 | 125.15 | 94.97 |

DALYs, disability-adjusted life years; ASDR, age-standardized disability-adjusted life years rate; EOCRC, early-onset colorectal cancer; LOCRC, late-onset colorectal cancer; BMI, body mass index

# Table S5 the predicted Deaths and ASMR of EOCRC and LOCRC attributable to HBMI from 2021 to 2040 based on Nordpred model

| Year | EOCRC | | | | | | LOCRC | | | | | |
| --- | --- | --- | --- | --- | --- | --- | --- | --- | --- | --- | --- | --- |
|  | Deaths | | | ASMR | | | Deaths | | | ASMR | | |
|  | Both | Male | Female | Both | Male | Female | Both | Male | Female | Both | Male | Female |
| 1992 | 3,392 | 1,774 | 1,619 | 0.17 | 0.17 | 0.16 | 40,704 | 18,522 | 22,182 | 4.96 | 5.11 | 4.81 |
| 1993 | 3,564 | 1,867 | 1,697 | 0.17 | 0.18 | 0.17 | 42,150 | 19,236 | 22,914 | 5.01 | 5.16 | 4.86 |
| 1994 | 3,747 | 1,966 | 1,781 | 0.17 | 0.18 | 0.17 | 43,331 | 19,861 | 23,470 | 5.04 | 5.20 | 4.87 |
| 1995 | 3,900 | 2,047 | 1,853 | 0.18 | 0.18 | 0.17 | 44,293 | 20,429 | 23,865 | 5.03 | 5.23 | 4.85 |
| 1996 | 4,024 | 2,115 | 1,909 | 0.18 | 0.18 | 0.17 | 45,089 | 20,909 | 24,180 | 5.01 | 5.23 | 4.80 |
| 1997 | 4,126 | 2,171 | 1,955 | 0.18 | 0.18 | 0.17 | 46,086 | 21,456 | 24,631 | 5.00 | 5.24 | 4.78 |
| 1998 | 4,223 | 2,224 | 1,999 | 0.18 | 0.18 | 0.17 | 47,347 | 22,135 | 25,211 | 5.01 | 5.27 | 4.77 |
| 1999 | 4,369 | 2,298 | 2,071 | 0.18 | 0.18 | 0.17 | 48,959 | 22,977 | 25,982 | 5.05 | 5.33 | 4.79 |
| 2000 | 4,513 | 2,396 | 2,117 | 0.18 | 0.19 | 0.17 | 50,288 | 23,767 | 26,522 | 5.06 | 5.37 | 4.77 |
| 2001 | 4,631 | 2,473 | 2,158 | 0.18 | 0.19 | 0.17 | 51,639 | 24,550 | 27,090 | 5.06 | 5.40 | 4.75 |
| 2002 | 4,746 | 2,541 | 2,204 | 0.18 | 0.19 | 0.17 | 53,101 | 25,411 | 27,690 | 5.06 | 5.44 | 4.73 |
| 2003 | 4,859 | 2,608 | 2,251 | 0.18 | 0.19 | 0.17 | 54,656 | 26,267 | 28,389 | 5.07 | 5.47 | 4.73 |
| 2004 | 4,942 | 2,664 | 2,277 | 0.18 | 0.19 | 0.17 | 55,697 | 26,941 | 28,756 | 5.03 | 5.45 | 4.66 |
| 2005 | 5,020 | 2,710 | 2,310 | 0.18 | 0.20 | 0.17 | 57,157 | 27,801 | 29,357 | 5.02 | 5.47 | 4.63 |
| 2006 | 5,064 | 2,744 | 2,320 | 0.18 | 0.19 | 0.17 | 58,090 | 28,401 | 29,689 | 4.95 | 5.42 | 4.55 |
| 2007 | 5,188 | 2,827 | 2,361 | 0.18 | 0.20 | 0.17 | 59,572 | 29,279 | 30,293 | 4.93 | 5.42 | 4.51 |
| 2008 | 5,386 | 2,945 | 2,441 | 0.18 | 0.20 | 0.17 | 61,625 | 30,454 | 31,171 | 4.95 | 5.47 | 4.51 |
| 2009 | 5,566 | 3,060 | 2,506 | 0.19 | 0.20 | 0.17 | 63,065 | 31,358 | 31,706 | 4.92 | 5.47 | 4.45 |
| 2010 | 5,734 | 3,179 | 2,555 | 0.19 | 0.21 | 0.17 | 65,039 | 32,514 | 32,525 | 4.92 | 5.50 | 4.43 |
| 2011 | 5,862 | 3,261 | 2,601 | 0.19 | 0.21 | 0.17 | 66,998 | 33,574 | 33,424 | 4.92 | 5.51 | 4.42 |
| 2012 | 6,002 | 3,353 | 2,649 | 0.19 | 0.21 | 0.17 | 69,045 | 34,773 | 34,272 | 4.91 | 5.52 | 4.39 |
| 2013 | 6,154 | 3,442 | 2,712 | 0.19 | 0.21 | 0.17 | 70,989 | 35,880 | 35,109 | 4.89 | 5.52 | 4.36 |
| 2014 | 6,281 | 3,515 | 2,766 | 0.19 | 0.22 | 0.17 | 73,106 | 36,981 | 36,125 | 4.88 | 5.52 | 4.35 |
| 2015 | 6,439 | 3,615 | 2,824 | 0.20 | 0.22 | 0.17 | 75,966 | 38,520 | 37,446 | 4.92 | 5.57 | 4.38 |
| 2016 | 6,629 | 3,730 | 2,899 | 0.20 | 0.22 | 0.18 | 78,853 | 40,058 | 38,795 | 4.95 | 5.61 | 4.40 |
| 2017 | 6,782 | 3,821 | 2,960 | 0.20 | 0.23 | 0.18 | 81,246 | 41,301 | 39,945 | 4.94 | 5.60 | 4.39 |
| 2018 | 6,945 | 3,911 | 3,034 | 0.21 | 0.23 | 0.18 | 84,164 | 42,869 | 41,295 | 4.96 | 5.63 | 4.40 |
| 2019 | 7,092 | 4,005 | 3,088 | 0.21 | 0.23 | 0.18 | 87,078 | 44,419 | 42,659 | 4.97 | 5.65 | 4.41 |
| 2020 | 7,123 | 4,023 | 3,101 | 0.21 | 0.23 | 0.18 | 89,243 | 45,646 | 43,598 | 4.95 | 5.64 | 4.38 |
| 2021 | 7,255 | 4,096 | 3,159 | 0.21 | 0.24 | 0.19 | 92,013 | 46,880 | 45,133 | 4.98 | 5.65 | 4.42 |
| 2022 | 7,568 | 4,282 | 3,286 | 0.21 | 0.24 | 0.19 | 96,020 | 49,200 | 46,820 | 4.99 | 5.68 | 4.42 |
| 2023 | 7,722 | 4,374 | 3,347 | 0.22 | 0.24 | 0.19 | 99,071 | 50,816 | 48,255 | 5.00 | 5.69 | 4.42 |
| 2024 | 7,882 | 4,471 | 3,411 | 0.22 | 0.25 | 0.19 | 102,347 | 52,544 | 49,802 | 5.01 | 5.70 | 4.43 |
| 2025 | 8,028 | 4,558 | 3,469 | 0.22 | 0.25 | 0.19 | 105,730 | 54,298 | 51,431 | 5.02 | 5.71 | 4.43 |
| 2026 | 8,177 | 4,649 | 3,528 | 0.22 | 0.25 | 0.19 | 109,147 | 56,067 | 53,080 | 5.03 | 5.73 | 4.44 |
| 2027 | 8,330 | 4,742 | 3,588 | 0.22 | 0.25 | 0.19 | 112,532 | 57,814 | 54,718 | 5.04 | 5.74 | 4.45 |
| 2028 | 8,492 | 4,840 | 3,652 | 0.22 | 0.26 | 0.19 | 115,999 | 59,598 | 56,401 | 5.05 | 5.75 | 4.45 |
| 2029 | 8,665 | 4,944 | 3,721 | 0.23 | 0.26 | 0.19 | 119,645 | 61,470 | 58,176 | 5.06 | 5.76 | 4.46 |
| 2030 | 8,811 | 5,031 | 3,780 | 0.23 | 0.26 | 0.19 | 123,352 | 63,340 | 60,012 | 5.06 | 5.76 | 4.47 |
| 2031 | 8,949 | 5,113 | 3,836 | 0.23 | 0.26 | 0.20 | 127,110 | 65,228 | 61,883 | 5.07 | 5.77 | 4.48 |
| 2032 | 9,068 | 5,186 | 3,882 | 0.23 | 0.26 | 0.20 | 130,866 | 67,104 | 63,762 | 5.08 | 5.78 | 4.48 |
| 2033 | 9,168 | 5,248 | 3,920 | 0.23 | 0.26 | 0.20 | 134,720 | 69,023 | 65,697 | 5.09 | 5.79 | 4.49 |
| 2034 | 9,252 | 5,302 | 3,950 | 0.23 | 0.26 | 0.20 | 138,749 | 71,024 | 67,725 | 5.09 | 5.79 | 4.50 |
| 2035 | 9,289 | 5,317 | 3,971 | 0.23 | 0.26 | 0.20 | 142,784 | 72,998 | 69,786 | 5.10 | 5.79 | 4.50 |
| 2036 | 9,316 | 5,328 | 3,987 | 0.23 | 0.26 | 0.20 | 146,845 | 74,975 | 71,870 | 5.10 | 5.80 | 4.51 |
| 2037 | 9,336 | 5,337 | 3,999 | 0.23 | 0.26 | 0.20 | 150,865 | 76,921 | 73,944 | 5.11 | 5.80 | 4.52 |
| 2038 | 9,343 | 5,340 | 4,003 | 0.23 | 0.26 | 0.20 | 154,954 | 78,890 | 76,063 | 5.11 | 5.80 | 4.52 |
| 2039 | 9,336 | 5,336 | 3,999 | 0.23 | 0.26 | 0.20 | 159,194 | 80,927 | 78,267 | 5.12 | 5.80 | 4.53 |
| 2040 | 9,325 | 5,332 | 3,993 | 0.23 | 0.26 | 0.20 | 163,487 | 82,984 | 80,503 | 5.12 | 5.80 | 4.53 |

EOCRC, early-onset colorectal cancer; LOCRC, late-onset colorectal cancer; BMI, body mass index; ASMR, age-standardized mortality rate

# Table S6 the predicted DALYs and ASDR of EOCRC and LOCRC attributable to high BMI from 2021 to 2040 based on BAPC model

| Year | **EOCRC** | | | | | | **LOCRC** | | | | | |
| --- | --- | --- | --- | --- | --- | --- | --- | --- | --- | --- | --- | --- |
|  | DALYs | | | ASDR | | | DALYs | | | ASDR | | |
|  | Both | Male | Female | Both | Male | Female | Both | Male | Female | Both | Male | Female |
| 1992 | 155,667 | 80,882 | 74,785 | 8.07 | 8.22 | 7.91 | 859,332 | 408,388 | 450,944 | 101.80 | 105.52 | 98.43 |
| 1993 | 161,512 | 84,284 | 77,228 | 8.16 | 8.35 | 7.96 | 882,775 | 421,047 | 461,728 | 102.26 | 106.16 | 98.69 |
| 1994 | 167,999 | 88,021 | 79,979 | 8.24 | 8.47 | 8.01 | 908,555 | 436,253 | 472,302 | 103.02 | 107.46 | 98.94 |
| 1995 | 176,251 | 92,539 | 83,712 | 8.40 | 8.65 | 8.14 | 940,243 | 453,262 | 486,981 | 104.37 | 109.06 | 100.02 |
| 1996 | 184,868 | 97,250 | 87,619 | 8.54 | 8.81 | 8.25 | 965,366 | 467,600 | 497,766 | 105.00 | 110.09 | 100.26 |
| 1997 | 192,129 | 101,115 | 91,015 | 8.61 | 8.90 | 8.31 | 983,239 | 479,345 | 503,893 | 104.89 | 110.63 | 99.58 |
| 1998 | 198,058 | 104,325 | 93,734 | 8.62 | 8.92 | 8.30 | 997,388 | 489,066 | 508,322 | 104.29 | 110.58 | 98.49 |
| 1999 | 203,053 | 107,088 | 95,966 | 8.63 | 8.95 | 8.29 | 1,016,916 | 500,450 | 516,466 | 103.98 | 110.63 | 97.89 |
| 2000 | 207,888 | 109,705 | 98,183 | 8.62 | 8.96 | 8.27 | 1,041,746 | 514,698 | 527,048 | 104.14 | 111.21 | 97.70 |
| 2001 | 214,811 | 113,320 | 101,491 | 8.73 | 9.08 | 8.37 | 1,076,209 | 533,319 | 542,891 | 105.00 | 112.44 | 98.27 |
| 2002 | 221,856 | 118,041 | 103,815 | 8.80 | 9.23 | 8.35 | 1,104,302 | 551,038 | 553,264 | 105.29 | 113.46 | 97.90 |
| 2003 | 227,711 | 121,842 | 105,869 | 8.86 | 9.35 | 8.35 | 1,133,858 | 568,789 | 565,069 | 105.43 | 114.19 | 97.56 |
| 2004 | 233,357 | 125,238 | 108,119 | 8.91 | 9.44 | 8.36 | 1,165,366 | 588,319 | 577,047 | 105.63 | 115.05 | 97.16 |
| 2005 | 238,880 | 128,529 | 110,351 | 8.95 | 9.52 | 8.38 | 1,198,170 | 606,952 | 591,218 | 105.84 | 115.64 | 97.08 |
| 2006 | 243,087 | 131,359 | 111,728 | 8.96 | 9.57 | 8.34 | 1,221,242 | 622,216 | 599,025 | 105.03 | 115.37 | 95.80 |
| 2007 | 247,144 | 133,760 | 113,384 | 8.97 | 9.60 | 8.32 | 1,254,014 | 642,098 | 611,916 | 104.97 | 115.84 | 95.29 |
| 2008 | 249,782 | 135,644 | 114,138 | 8.90 | 9.57 | 8.22 | 1,275,051 | 656,168 | 618,883 | 103.77 | 115.02 | 93.77 |
| 2009 | 256,024 | 139,779 | 116,244 | 8.95 | 9.68 | 8.21 | 1,307,397 | 676,322 | 631,075 | 103.42 | 115.12 | 93.00 |
| 2010 | 265,384 | 145,448 | 119,935 | 9.08 | 9.87 | 8.28 | 1,350,577 | 702,464 | 648,113 | 103.88 | 116.18 | 92.93 |
| 2011 | 274,083 | 151,013 | 123,069 | 9.18 | 10.04 | 8.30 | 1,381,107 | 721,995 | 659,113 | 103.25 | 116.02 | 91.91 |
| 2012 | 282,239 | 156,721 | 125,518 | 9.26 | 10.21 | 8.29 | 1,423,469 | 747,343 | 676,125 | 103.44 | 116.71 | 91.68 |
| 2013 | 288,513 | 160,662 | 127,850 | 9.29 | 10.28 | 8.29 | 1,465,053 | 771,146 | 693,906 | 103.38 | 116.90 | 91.40 |
| 2014 | 295,339 | 165,033 | 130,306 | 9.36 | 10.40 | 8.31 | 1,509,539 | 797,990 | 711,549 | 103.36 | 117.33 | 90.99 |
| 2015 | 302,882 | 169,504 | 133,378 | 9.48 | 10.55 | 8.41 | 1,551,643 | 822,772 | 728,871 | 103.05 | 117.29 | 90.45 |
| 2016 | 309,661 | 173,507 | 136,154 | 9.60 | 10.69 | 8.49 | 1,597,503 | 847,201 | 750,301 | 102.89 | 117.10 | 90.33 |
| 2017 | 317,749 | 178,635 | 139,115 | 9.76 | 10.90 | 8.60 | 1,659,143 | 881,308 | 777,835 | 103.67 | 118.16 | 90.88 |
| 2018 | 327,194 | 184,413 | 142,782 | 9.95 | 11.14 | 8.74 | 1,721,163 | 915,184 | 805,979 | 104.32 | 119.00 | 91.39 |
| 2019 | 335,129 | 189,251 | 145,878 | 10.11 | 11.33 | 8.86 | 1,772,632 | 942,462 | 830,171 | 104.25 | 118.86 | 91.36 |
| 2020 | 343,399 | 193,963 | 149,437 | 10.27 | 11.52 | 9.00 | 1,835,387 | 977,176 | 858,211 | 104.75 | 119.54 | 91.71 |
| 2021 | 350,815 | 198,690 | 152,125 | 10.40 | 11.69 | 9.08 | 1,897,093 | 1,011,306 | 885,787 | 105.11 | 120.04 | 91.92 |
| 2022 | 352,976 | 199,891 | 153,085 | 10.38 | 11.67 | 9.06 | 1,942,250 | 1,036,958 | 905,292 | 104.71 | 119.83 | 91.39 |
| 2023 | 359,552 | 203,657 | 155,894 | 10.49 | 11.80 | 9.16 | 2,005,037 | 1,065,268 | 939,769 | 105.45 | 120.20 | 92.49 |
| 2024 | 375,420 | 213,368 | 162,052 | 10.65 | 12.05 | 9.24 | 2,095,229 | 1,122,251 | 972,977 | 106.33 | 121.93 | 92.48 |
| 2025 | 382,987 | 217,890 | 165,097 | 10.76 | 12.18 | 9.31 | 2,159,665 | 1,157,180 | 1,002,484 | 106.67 | 122.35 | 92.73 |
| 2026 | 391,014 | 222,746 | 168,268 | 10.86 | 12.32 | 9.38 | 2,226,017 | 1,192,843 | 1,033,174 | 107.00 | 122.73 | 92.97 |
| 2027 | 399,173 | 227,707 | 171,465 | 10.97 | 12.46 | 9.44 | 2,294,654 | 1,229,535 | 1,065,118 | 107.37 | 123.13 | 93.25 |
| 2028 | 407,455 | 232,752 | 174,703 | 11.07 | 12.59 | 9.51 | 2,365,628 | 1,267,448 | 1,098,180 | 107.82 | 123.61 | 93.59 |
| 2029 | 415,925 | 237,972 | 177,953 | 11.17 | 12.72 | 9.57 | 2,437,045 | 1,306,221 | 1,130,825 | 108.30 | 124.16 | 93.89 |
| 2030 | 424,945 | 243,571 | 181,374 | 11.28 | 12.86 | 9.63 | 2,509,293 | 1,345,250 | 1,164,043 | 108.80 | 124.69 | 94.19 |
| 2031 | 434,432 | 249,508 | 184,924 | 11.39 | 12.99 | 9.69 | 2,582,908 | 1,384,761 | 1,198,148 | 109.30 | 125.19 | 94.48 |
| 2032 | 443,872 | 255,486 | 188,386 | 11.50 | 13.12 | 9.74 | 2,659,031 | 1,425,348 | 1,233,683 | 109.85 | 125.70 | 94.81 |
| 2033 | 452,962 | 261,306 | 191,656 | 11.61 | 13.24 | 9.79 | 2,738,662 | 1,467,688 | 1,270,974 | 110.51 | 126.28 | 95.20 |
| 2034 | 461,281 | 266,685 | 194,597 | 11.71 | 13.36 | 9.84 | 2,820,411 | 1,511,759 | 1,308,652 | 111.22 | 126.94 | 95.56 |
| 2035 | 468,725 | 271,481 | 197,244 | 11.82 | 13.46 | 9.90 | 2,904,785 | 1,557,129 | 1,347,656 | 111.97 | 127.59 | 95.91 |
| 2036 | 475,439 | 275,720 | 199,719 | 11.93 | 13.54 | 9.95 | 2,992,209 | 1,603,906 | 1,388,303 | 112.75 | 128.20 | 96.25 |
| 2037 | 481,461 | 279,406 | 202,055 | 12.04 | 13.61 | 10.01 | 3,083,326 | 1,652,365 | 1,430,961 | 113.61 | 128.83 | 96.63 |
| 2038 | 486,937 | 282,632 | 204,304 | 12.14 | 13.66 | 10.08 | 3,178,672 | 1,702,935 | 1,475,738 | 114.59 | 129.52 | 97.07 |
| 2039 | 491,997 | 285,515 | 206,482 | 12.24 | 13.68 | 10.14 | 3,276,353 | 1,755,361 | 1,520,993 | 115.65 | 130.27 | 97.48 |
| 2040 | 496,593 | 288,109 | 208,484 | 12.34 | 13.70 | 10.20 | 3,377,296 | 1,809,522 | 1,567,774 | 116.78 | 131.01 | 97.87 |

DALYs, disability-adjusted life years; ASDR, age-standardized disability-adjusted life years rate; EOCRC, early-onset colorectal cancer; LOCRC, late-onset colorectal cancer; BMI, body mass index; BAPC, Bayesian age-period-cohort

# Table S7 the predicted Deaths and ASMR of EOCRC and LOCRC attributable to HBMI from 2021 to 2040 based on BAPC model

| Year | EOCRC | | | | | | LOCRC | | | | | |
| --- | --- | --- | --- | --- | --- | --- | --- | --- | --- | --- | --- | --- |
|  | Deaths | | | ASMR | | | Deaths | | | ASMR | | |
|  | Both | Male | Female | Both | Male | Female | Both | Male | Female | Both | Male | Female |
| 1992 | 3,162 | 1,641 | 1,521 | 0.17 | 0.17 | 0.16 | 38,389 | 17,313 | 21,076 | 4.90 | 5.01 | 4.79 |
| 1993 | 3,277 | 1,706 | 1,571 | 0.17 | 0.17 | 0.16 | 39,554 | 17,902 | 21,652 | 4.93 | 5.05 | 4.80 |
| 1994 | 3,411 | 1,781 | 1,630 | 0.17 | 0.17 | 0.17 | 40,769 | 18,538 | 22,231 | 4.96 | 5.10 | 4.82 |
| 1995 | 3,559 | 1,861 | 1,697 | 0.17 | 0.18 | 0.17 | 42,045 | 19,196 | 22,849 | 5.00 | 5.15 | 4.85 |
| 1996 | 3,720 | 1,949 | 1,770 | 0.17 | 0.18 | 0.17 | 43,197 | 19,812 | 23,386 | 5.02 | 5.19 | 4.85 |
| 1997 | 3,873 | 2,032 | 1,840 | 0.18 | 0.18 | 0.17 | 44,214 | 20,387 | 23,827 | 5.03 | 5.22 | 4.84 |
| 1998 | 4,019 | 2,112 | 1,907 | 0.18 | 0.18 | 0.17 | 45,150 | 20,925 | 24,225 | 5.02 | 5.24 | 4.81 |
| 1999 | 4,138 | 2,178 | 1,960 | 0.18 | 0.18 | 0.17 | 46,220 | 21,515 | 24,705 | 5.01 | 5.26 | 4.79 |
| 2000 | 4,258 | 2,246 | 2,012 | 0.18 | 0.18 | 0.17 | 47,476 | 22,198 | 25,278 | 5.03 | 5.29 | 4.78 |
| 2001 | 4,377 | 2,312 | 2,064 | 0.18 | 0.19 | 0.17 | 48,925 | 22,983 | 25,941 | 5.05 | 5.34 | 4.79 |
| 2002 | 4,511 | 2,394 | 2,117 | 0.18 | 0.19 | 0.17 | 50,275 | 23,761 | 26,514 | 5.06 | 5.38 | 4.77 |
| 2003 | 4,623 | 2,464 | 2,159 | 0.18 | 0.19 | 0.17 | 51,645 | 24,547 | 27,097 | 5.06 | 5.41 | 4.75 |
| 2004 | 4,732 | 2,531 | 2,201 | 0.18 | 0.19 | 0.17 | 53,066 | 25,379 | 27,688 | 5.06 | 5.44 | 4.73 |
| 2005 | 4,836 | 2,596 | 2,241 | 0.18 | 0.19 | 0.17 | 54,499 | 26,204 | 28,296 | 5.06 | 5.47 | 4.71 |
| 2006 | 4,926 | 2,654 | 2,272 | 0.18 | 0.19 | 0.17 | 55,719 | 26,946 | 28,774 | 5.04 | 5.47 | 4.66 |
| 2007 | 5,017 | 2,711 | 2,306 | 0.18 | 0.19 | 0.17 | 57,063 | 27,751 | 29,312 | 5.02 | 5.47 | 4.62 |
| 2008 | 5,103 | 2,769 | 2,334 | 0.18 | 0.20 | 0.17 | 58,240 | 28,474 | 29,766 | 4.97 | 5.45 | 4.56 |
| 2009 | 5,226 | 2,848 | 2,378 | 0.18 | 0.20 | 0.17 | 59,718 | 29,359 | 30,359 | 4.95 | 5.45 | 4.52 |
| 2010 | 5,386 | 2,948 | 2,438 | 0.18 | 0.20 | 0.17 | 61,513 | 30,414 | 31,099 | 4.95 | 5.48 | 4.50 |
| 2011 | 5,553 | 3,054 | 2,499 | 0.19 | 0.20 | 0.17 | 63,142 | 31,391 | 31,750 | 4.93 | 5.48 | 4.46 |
| 2012 | 5,720 | 3,164 | 2,556 | 0.19 | 0.21 | 0.17 | 65,016 | 32,486 | 32,531 | 4.92 | 5.50 | 4.44 |
| 2013 | 5,873 | 3,262 | 2,610 | 0.19 | 0.21 | 0.17 | 66,937 | 33,560 | 33,377 | 4.91 | 5.51 | 4.41 |
| 2014 | 6,027 | 3,363 | 2,664 | 0.19 | 0.21 | 0.17 | 68,987 | 34,736 | 34,251 | 4.91 | 5.52 | 4.39 |
| 2015 | 6,172 | 3,452 | 2,720 | 0.19 | 0.21 | 0.17 | 71,074 | 35,901 | 35,172 | 4.90 | 5.53 | 4.37 |
| 2016 | 6,301 | 3,529 | 2,773 | 0.19 | 0.22 | 0.17 | 73,308 | 37,088 | 36,220 | 4.90 | 5.53 | 4.37 |
| 2017 | 6,447 | 3,619 | 2,828 | 0.20 | 0.22 | 0.17 | 75,943 | 38,510 | 37,434 | 4.92 | 5.57 | 4.38 |
| 2018 | 6,609 | 3,719 | 2,890 | 0.20 | 0.22 | 0.18 | 78,708 | 39,982 | 38,726 | 4.94 | 5.60 | 4.39 |
| 2019 | 6,758 | 3,806 | 2,952 | 0.20 | 0.23 | 0.18 | 81,278 | 41,330 | 39,948 | 4.94 | 5.60 | 4.39 |
| 2020 | 6,907 | 3,891 | 3,016 | 0.21 | 0.23 | 0.18 | 84,120 | 42,857 | 41,263 | 4.96 | 5.62 | 4.40 |
| 2021 | 7,053 | 3,981 | 3,072 | 0.21 | 0.23 | 0.18 | 86,990 | 44,382 | 42,608 | 4.97 | 5.63 | 4.40 |
| 2022 | 7,160 | 4,043 | 3,117 | 0.21 | 0.24 | 0.18 | 89,398 | 45,673 | 43,725 | 4.96 | 5.64 | 4.39 |
| 2023 | 7,291 | 4,118 | 3,173 | 0.21 | 0.24 | 0.19 | 91,986 | 46,898 | 45,087 | 4.97 | 5.64 | 4.41 |
| 2024 | 7,614 | 4,302 | 3,313 | 0.22 | 0.24 | 0.19 | 95,978 | 49,196 | 46,782 | 4.99 | 5.66 | 4.41 |
| 2025 | 7,793 | 4,408 | 3,385 | 0.22 | 0.25 | 0.19 | 99,014 | 50,792 | 48,222 | 4.99 | 5.67 | 4.42 |
| 2026 | 7,981 | 4,520 | 3,461 | 0.22 | 0.25 | 0.19 | 102,287 | 52,496 | 49,791 | 5.00 | 5.68 | 4.42 |
| 2027 | 8,178 | 4,639 | 3,539 | 0.22 | 0.25 | 0.19 | 105,726 | 54,281 | 51,445 | 5.02 | 5.70 | 4.43 |
| 2028 | 8,383 | 4,763 | 3,620 | 0.23 | 0.26 | 0.20 | 109,257 | 56,114 | 53,143 | 5.03 | 5.71 | 4.44 |
| 2029 | 8,594 | 4,892 | 3,701 | 0.23 | 0.26 | 0.20 | 112,787 | 57,961 | 54,826 | 5.05 | 5.73 | 4.45 |
| 2030 | 8,816 | 5,029 | 3,787 | 0.23 | 0.26 | 0.20 | 116,428 | 59,859 | 56,569 | 5.06 | 5.75 | 4.46 |
| 2031 | 9,052 | 5,173 | 3,878 | 0.24 | 0.27 | 0.20 | 120,273 | 61,848 | 58,425 | 5.08 | 5.76 | 4.47 |
| 2032 | 9,290 | 5,320 | 3,970 | 0.24 | 0.27 | 0.20 | 124,305 | 63,924 | 60,381 | 5.10 | 5.78 | 4.48 |
| 2033 | 9,522 | 5,463 | 4,059 | 0.24 | 0.28 | 0.20 | 128,496 | 66,078 | 62,418 | 5.12 | 5.80 | 4.49 |
| 2034 | 9,737 | 5,597 | 4,140 | 0.25 | 0.28 | 0.21 | 132,760 | 68,287 | 64,473 | 5.15 | 5.83 | 4.50 |
| 2035 | 9,931 | 5,718 | 4,213 | 0.25 | 0.28 | 0.21 | 137,182 | 70,570 | 66,612 | 5.18 | 5.85 | 4.51 |
| 2036 | 10,109 | 5,829 | 4,281 | 0.25 | 0.28 | 0.21 | 141,847 | 72,963 | 68,884 | 5.21 | 5.88 | 4.53 |
| 2037 | 10,275 | 5,931 | 4,345 | 0.25 | 0.29 | 0.21 | 146,741 | 75,461 | 71,280 | 5.24 | 5.90 | 4.54 |
| 2038 | 10,431 | 6,026 | 4,405 | 0.26 | 0.29 | 0.21 | 151,825 | 78,056 | 73,769 | 5.28 | 5.93 | 4.55 |
| 2039 | 10,582 | 6,118 | 4,464 | 0.26 | 0.29 | 0.21 | 156,983 | 80,715 | 76,268 | 5.32 | 5.96 | 4.57 |
| 2040 | 10,721 | 6,203 | 4,518 | 0.26 | 0.29 | 0.22 | 162,306 | 83,459 | 78,847 | 5.36 | 5.99 | 4.58 |

EOCRC, early-onset colorectal cancer; LOCRC, late-onset colorectal cancer; BMI, body mass index; BAPC, Bayesian age-period-cohort; ASMR, age-standardized mortality rate
