# Supplementary material for: Global, regional, and national burden of early-onset and late-onset colorectal cancer attributable to high body-mass index from 1990 to 2021: a trend analysis and forecasts up to 2040 based on the global burden of disease study 2021
Source: BMC Gastroenterol. 2025 Nov 19;25:816. doi: 10.1186/s12876-025-04432-7 (PMC12629028; doi:10.1186/s12876-025-04432-7)
Supplement: Supplementary file 2 — Supplementary Material 2 [file 12876_2025_4432_MOESM2_ESM.docx]

**Supplementary Figures**

**Global, regional, and national burden of early-onset and late-onset colorectal cancer attributable to high body-mass index from 1990 to 2021: A trend analysis and forecasts up to 2040 based on the Global Burden of Disease Study 2021**

Bin Yue ^1^ *, Zhongqiao Lu ^2^, Desan Zong ^1^,Yingxia Hu ^1^, Zhongde Yang ^1^

^1^ Department of Gastroenterology, The People’s Hospital of Wenshan Prefecture; Affiliated Wenshan Hospital, KunMing University of Science and Technology, Wenshan 663000, Yunnan Province, China

^2^ Department of Cardiac and Vascular diseases, The People’s Hospital of Wenshan Prefecture; Affiliated Wenshan Hospital, KunMing University of Science and Technology, Wenshan 663000, Yunnan Province, China

*** Corresponding:**

Bin Yue

Department of Gastroenterology, The People’s Hospital of Wenshan Prefecture; Affiliated Wenshan Hospital, KunMing University of Science and Technology, Wenshan 663000, Yunnan Province, China.

Address: No. 31, Tenglong North Road, Wenshan, 663000, Yunnan, China.

Email: helloybin@163.com

Tel: +86 15393954526

Contents

[Figure S1. the trends of ASMR in EOCRC attributable HBMI in SDI regions 3](#_Toc190636844)

[Figure S2. the trends of ASMR in LOCRC attributable HBMI in SDI regions 4](#_Toc190636845)

[Figure S3. EAPC of ASMR in 21 GBD regions between 1990 and 2021 in EOCRC attributable HBMI 5](#_Toc190636846)

[Figure S4. EAPC of ASMR in 21 GBD regions between 1990 and 2021 in LOCRC attributable HBMI 6](#_Toc190636847)

[Figure S5. the correlation between ASMR and SDI in 21 GBD regions between 1990 and 2021 in EOCRC attributable HBMI 7](#_Toc190636848)

[Figure S6. the correlation between ASMR and SDI in 21 GBD regions between 1990 and 2021 in LOCRC attributable HBMI 8](#_Toc190636849)

[Figure S7. Spatial distribution of the ASMR between 2019 and 2021 in EOCRC attributable to HBMI 9](#_Toc190636850)

[Figure S8. Spatial distribution of the EAPC between 2019 and 2021 in EOCRC attributable to HBMI 10](#_Toc190636851)

[Figure S9. Spatial distribution of the ASMR between 2019 and 2021 in LOCRC attributable to HBMI 11](#_Toc190636852)

[Figure S10. Spatial distribution of the EAPC between 2019 and 2021 in LOCRC attributable to HBMI 12](#_Toc190636853)


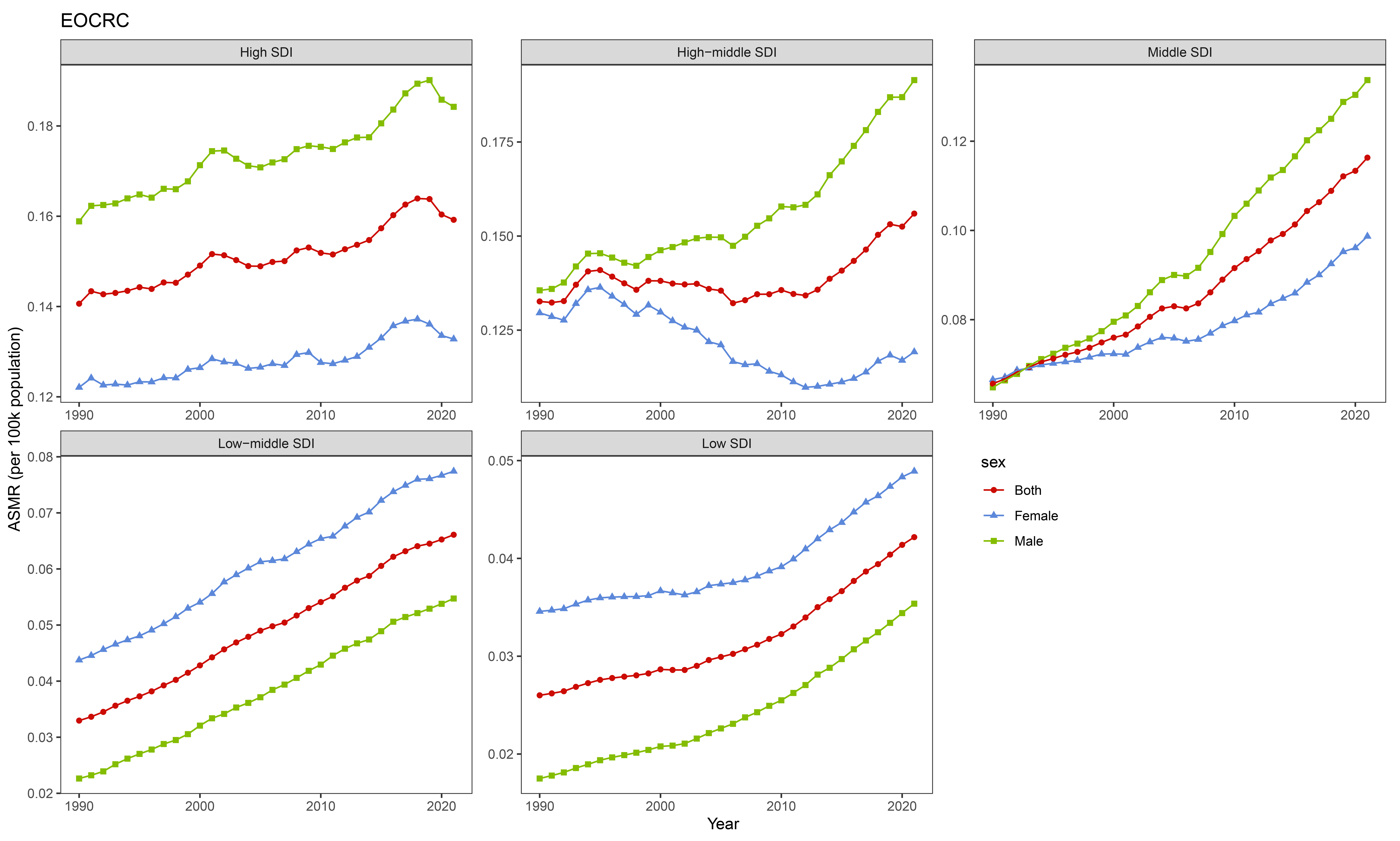


# Figure S1. the trends of ASMR in EOCRC attributable HBMI in SDI regions. ASMR, age-standardized mortality rate; EOCRC, early-onset colorectal cancer; BMI, body mass index; SDI, sociodemographic index


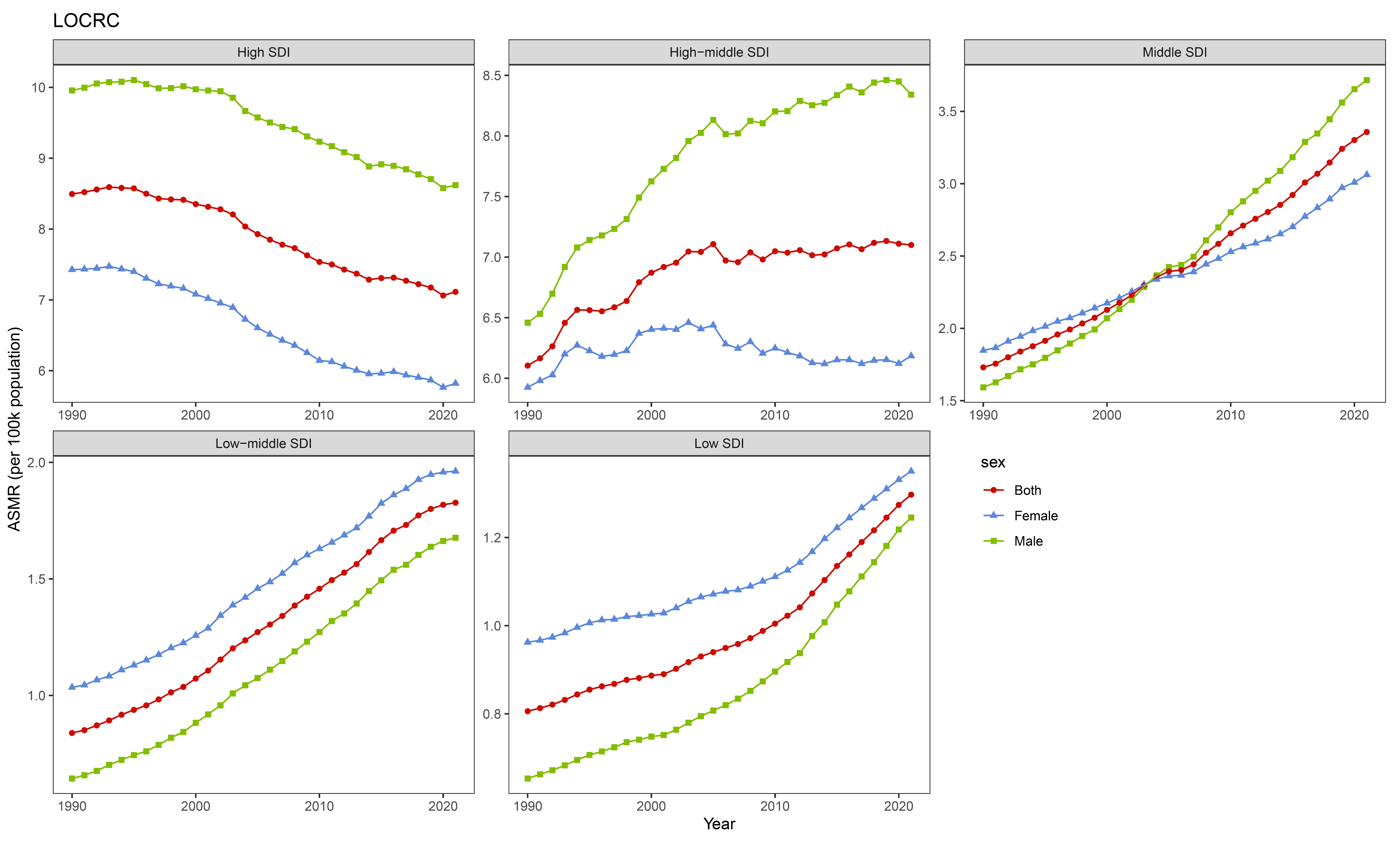


# Figure S2. the trends of ASMR in LOCRC attributable HBMI in SDI regions. ASMR, age-standardized mortality rate; LOCRC, late-onset colorectal cancer; BMI, body mass index; SDI, sociodemographic index


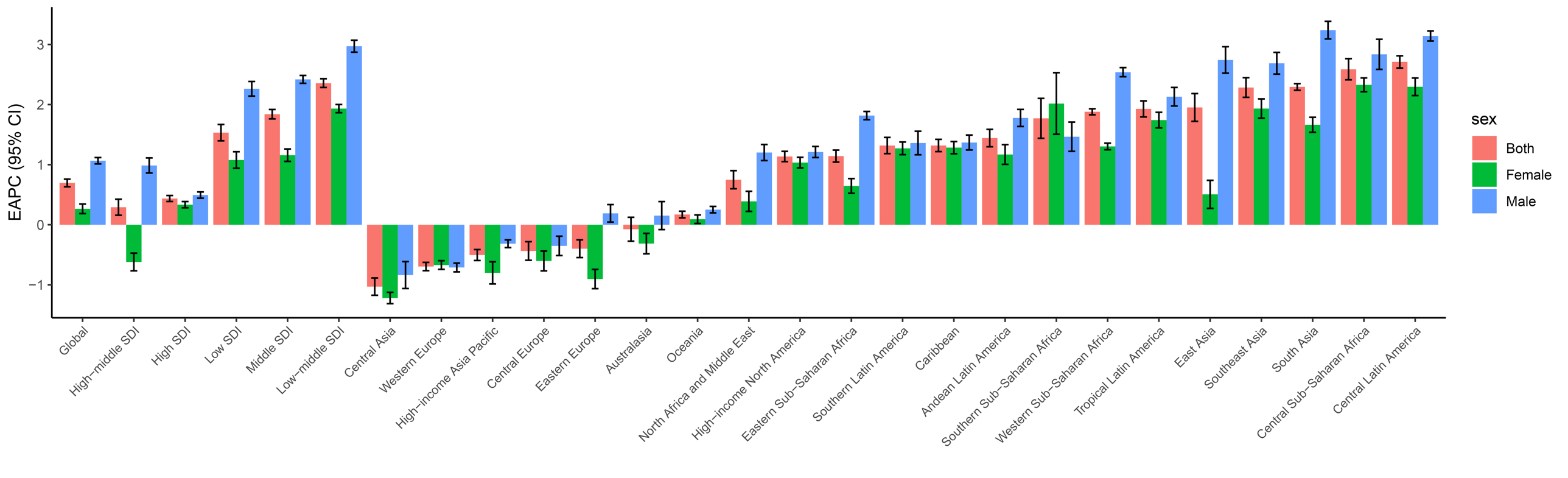


# Figure S3. EAPC of ASMR in 21 GBD regions between 1990 and 2021 in EOCRC attributable HBMI. EAPC, estimated annual percentage change; ASMR, age-standardized mortality rate; GBD, global burden disease; EOCRC, early-onset colorectal cancer; BMI, body mass index


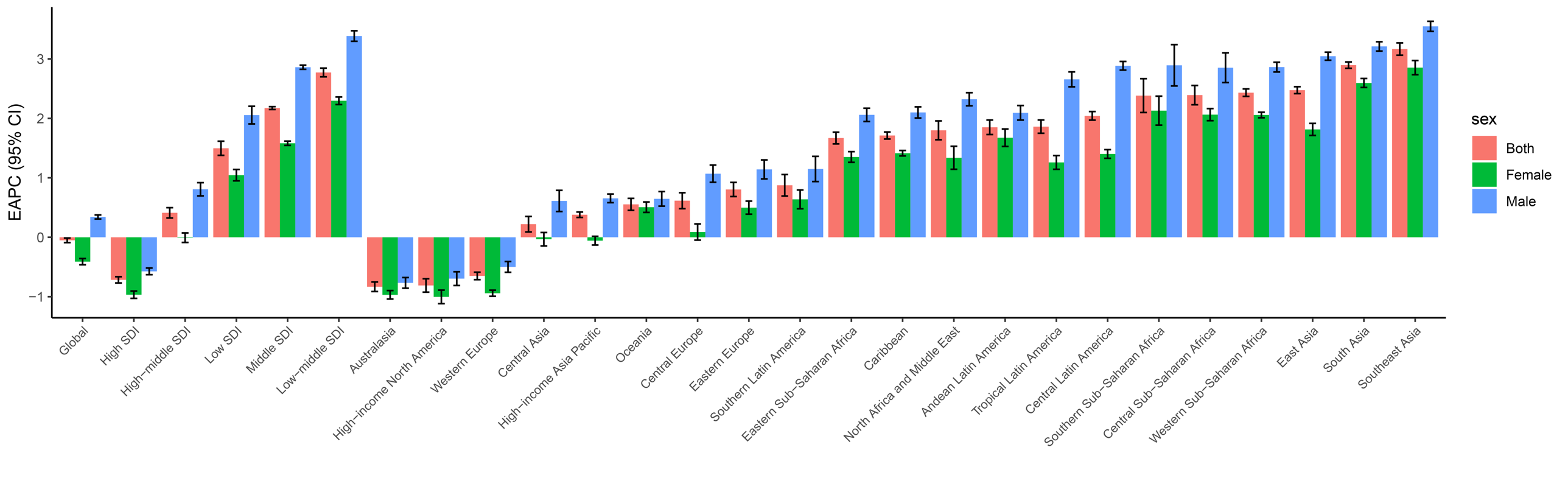


Figure S4. EAPC of ASMR in 21 GBD regions between 1990 and 2021 in LOCRC attributable HBMI. EAPC, estimated annual percentage change; ASMR, age-standardized mortality rate; GBD, global burden disease; LOCRC, late-onset colorectal cancer; BMI, body mass index


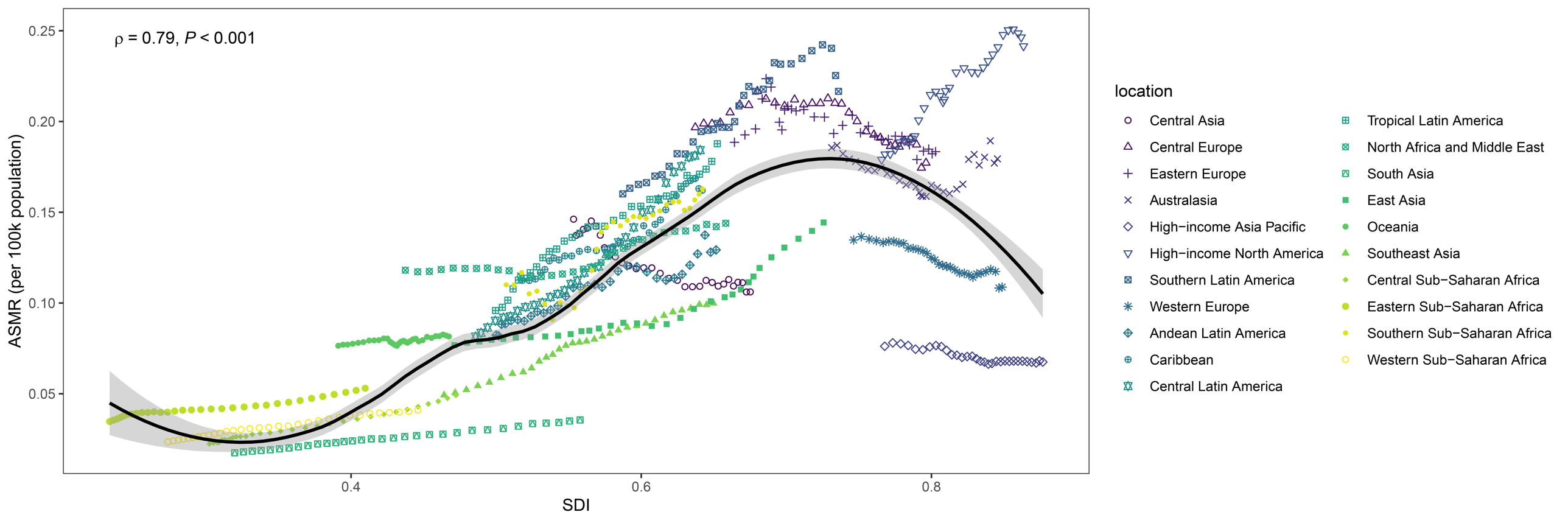


# Figure S5. the correlation between ASMR and SDI in 21 GBD regions between 1990 and 2021 in EOCRC attributable HBMI. ASMR, age-standardized mortality rate; SDI, sociodemographic index; GBD, global burden disease; EOCRC, early-onset colorectal cancer; BMI, body mass index


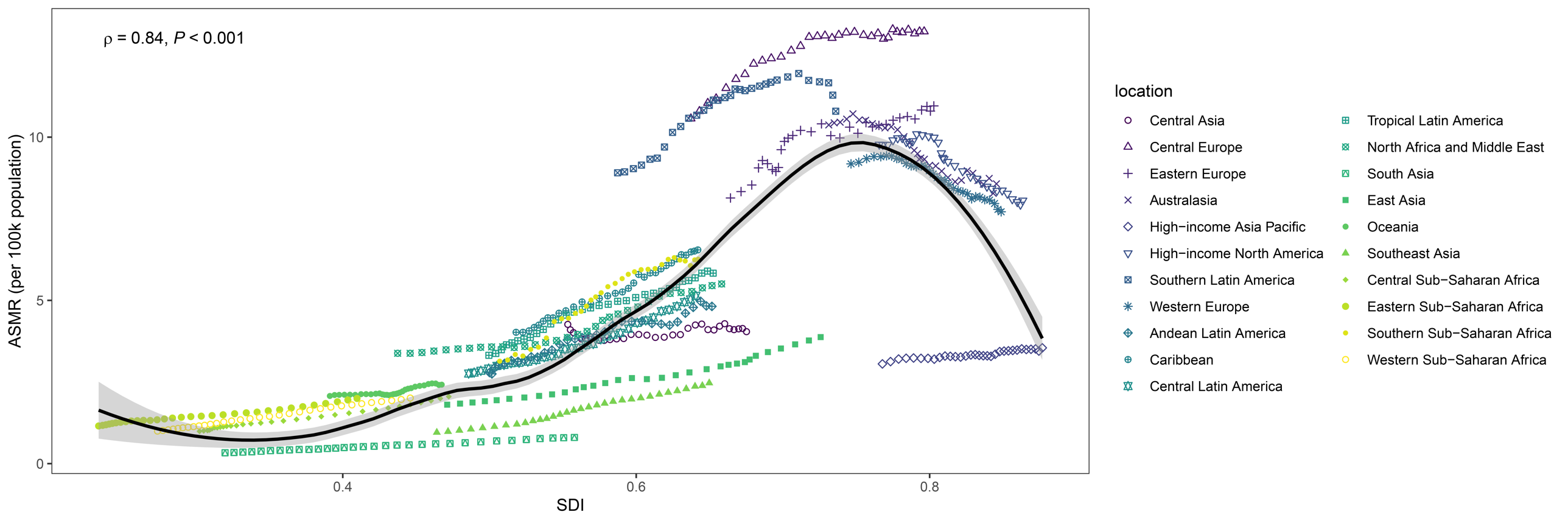


# Figure S6. the correlation between ASMR and SDI in 21 GBD regions between 1990 and 2021 in LOCRC attributable HBMI. ASMR, age-standardized mortality rate; SDI, sociodemographic index; GBD, global burden disease; LOCRC, late-onset colorectal cancer; BMI, body mass index


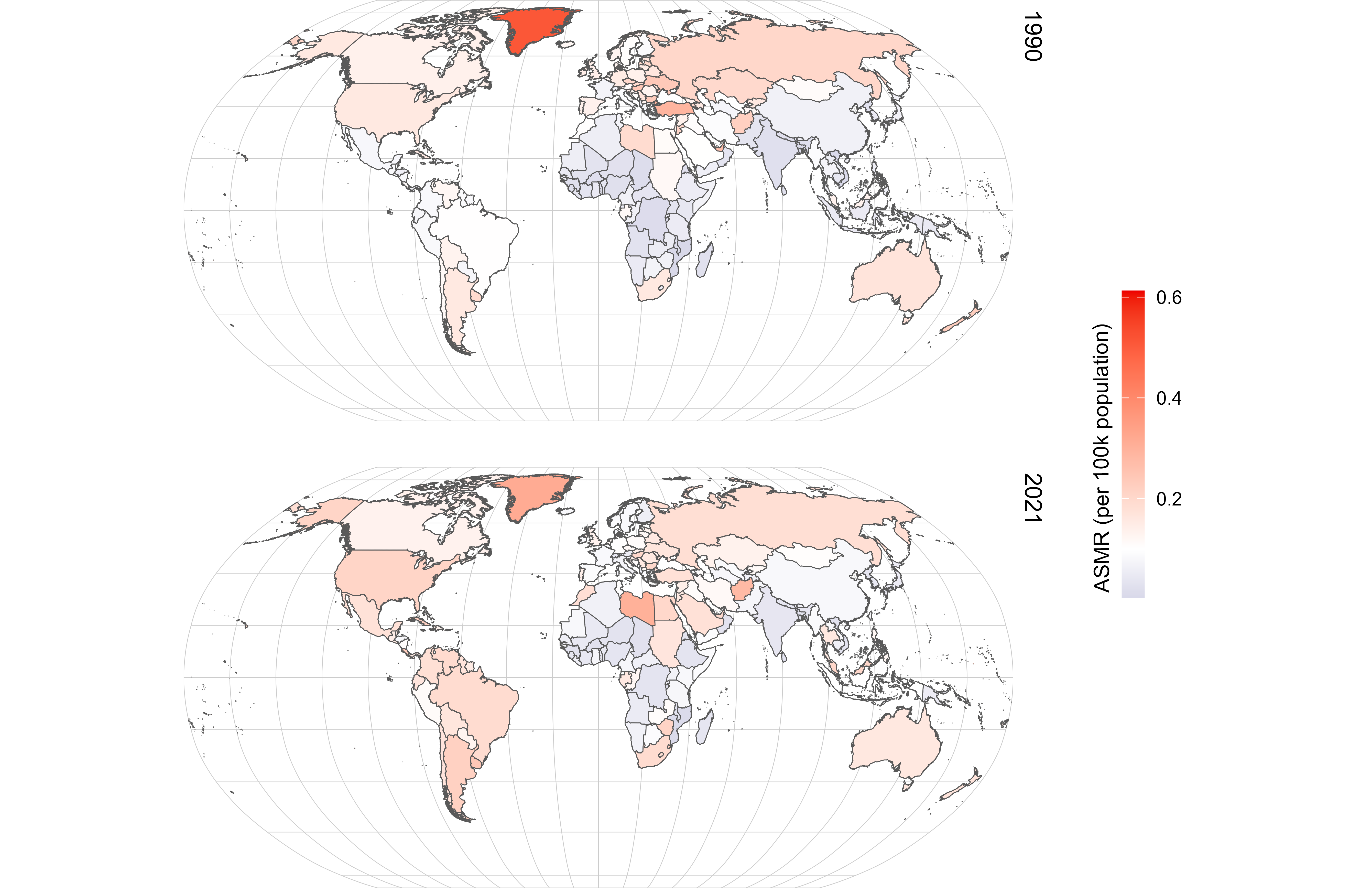


# Figure S7. Spatial distribution of the ASMR between 2019 and 2021 in EOCRC attributable to HBMI. ASMR, age-standardized mortality rate; EOCRC, early-onset colorectal cancer; BMI, body mass index


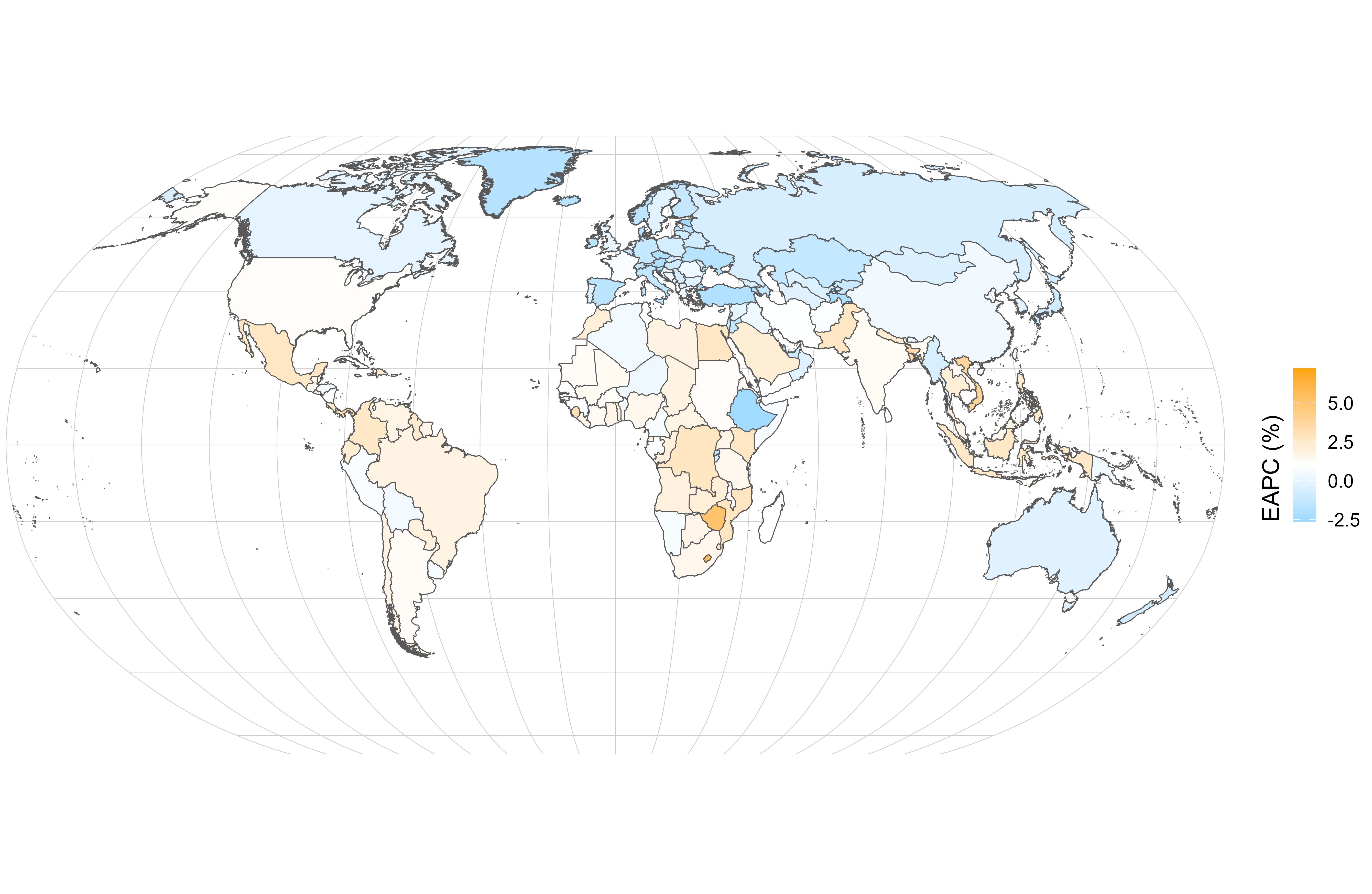


# Figure S8. Spatial distribution of the EAPC between 2019 and 2021 in EOCRC attributable to HBMI. EAPC, estimated annual percentage change; EOCRC, early-onset colorectal cancer; BMI, body mass index


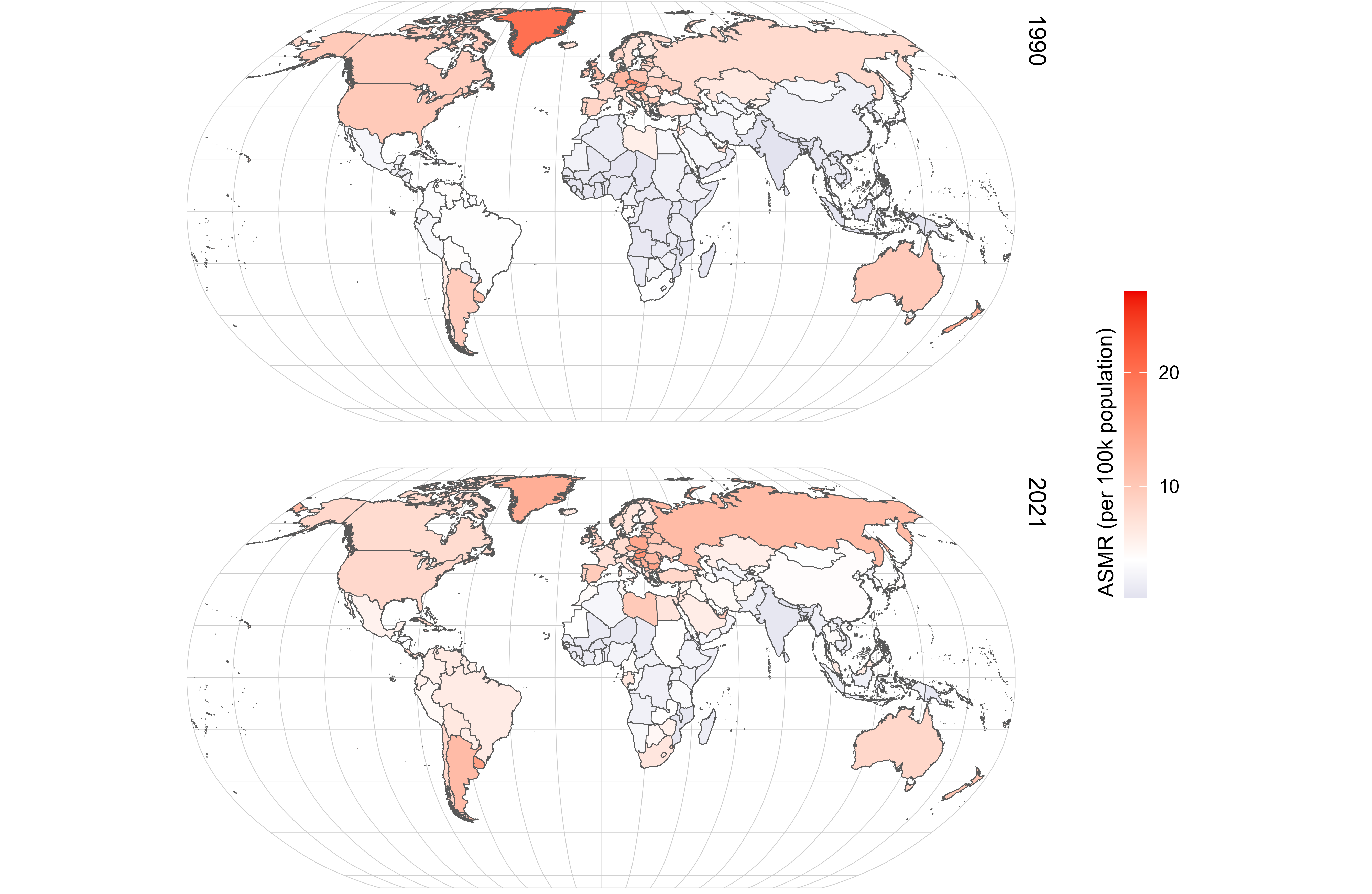


# Figure S9. Spatial distribution of the ASMR between 2019 and 2021 in LOCRC attributable to HBMI. ASMR, age-standardized mortality rate; LOCRC, late-onset colorectal cancer; BMI, body mass index


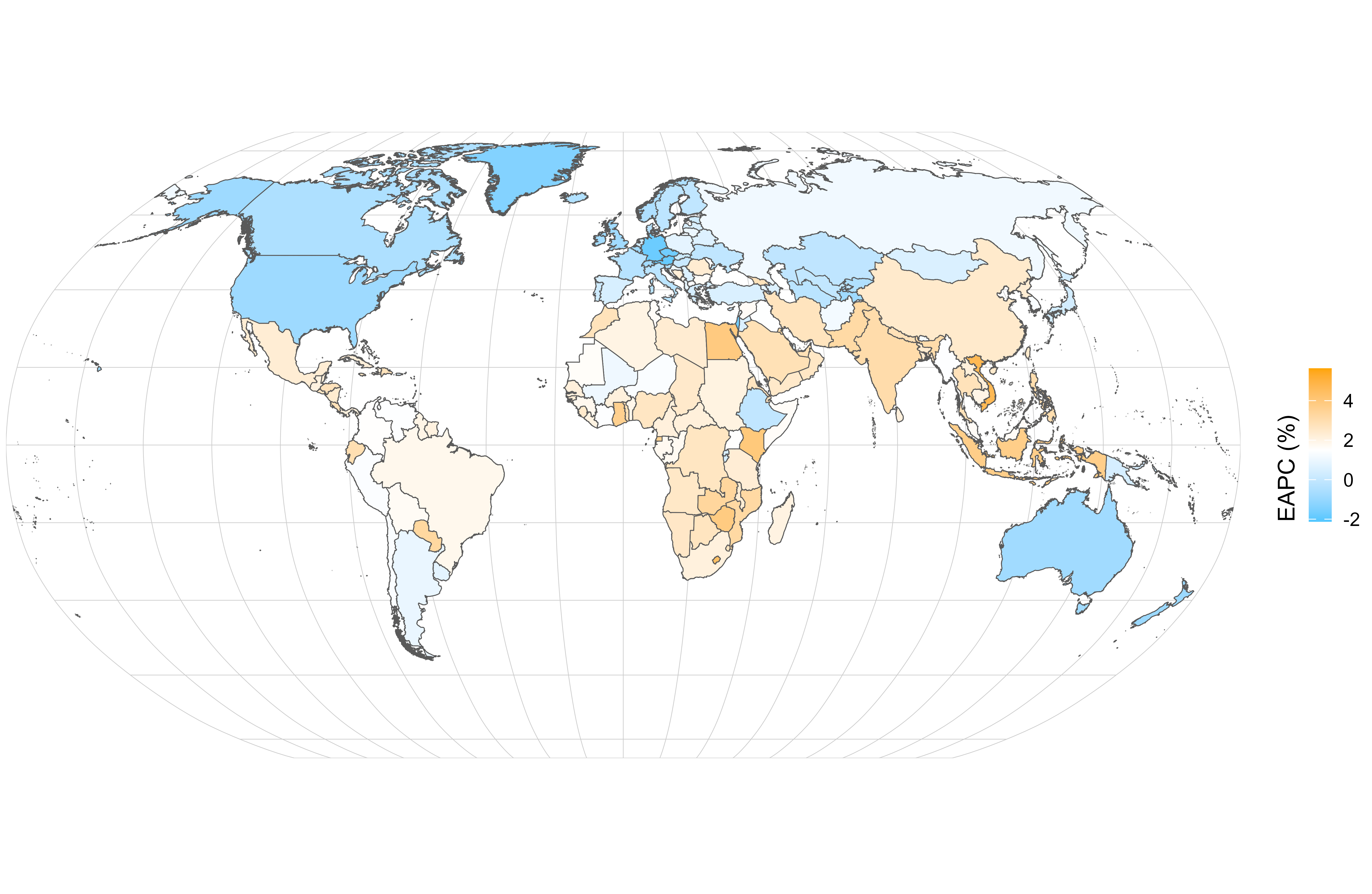


# Figure S10. Spatial distribution of the EAPC between 2019 and 2021 in LOCRC attributable to HBMI. EAPC, estimated annual percentage change; LOCRC, late-onset colorectal cancer; BMI, body mass index
